# Distributed algorithms from arboreal ants for the shortest path problem

**DOI:** 10.1073/pnas.2207959120

**Published:** 2023-01-30

**Authors:** Shivam Garg, Kirankumar Shiragur, Deborah M. Gordon, Moses Charikar

**Affiliations:** ^a^Department of Computer Science, Stanford University, Stanford, CA 94305; ^b^Department of Management Science and Engineering, Stanford University, Stanford, CA 94305; ^c^Department of Biology, Stanford University, Stanford, CA 94305

**Keywords:** natural algorithms, shortest path problem, ant colonies, distributed algorithms, graph algorithms

## Abstract

Colonies of the arboreal turtle ant create networks of trails that link nests and food sources on the graph formed by branches and vines in the canopy of the tropical forest. Ants put down a volatile pheromone on the edges as they traverse them. At each vertex, the next edge to traverse is chosen using a decision rule based on the current pheromone level. There is a bidirectional flow of ants around the network. In a previous field study, it was observed that the trail networks approximately minimize the number of vertices, thus solving a variant of the popular shortest path problem without any central control and with minimal computational resources. We propose a biologically plausible model, based on a variant of the reinforced random walk on a graph, which explains this observation and suggests surprising algorithms for the shortest path problem and its variants. Through simulations and analysis, we show that when the rate of flow of ants does not change, the dynamics converges to the path with the minimum number of vertices, as observed in the field. The dynamics converges to the shortest path when the rate of flow increases with time, so the colony can solve the shortest path problem merely by increasing the flow rate. We also show that to guarantee convergence to the shortest path, bidirectional flow and a decision rule dividing the flow in proportion to the pheromone level are necessary, but convergence to approximately short paths is possible with other decision rules.

## Significance

Evolution has led to natural algorithms that regulate collective behavior in many biological systems. Here, we investigate natural algorithms that solve the shortest path problem, a basic optimization problem on graphs. A recent field study showed that arboreal ants solve a variant of the shortest path problem while creating and maintaining trail networks. They do this without any central control and with minimal computational resources. We propose a model for how trail networks change over time that explains this phenomenon and leads to surprising algorithms for the shortest path problem and its variants.

Biological systems, such as ant trail networks, are fascinating examples of distributed algorithms in nature (1–4), often finding globally optimum solutions using simple local interactions among individuals, devoid of central control. The study of natural algorithms has led to synergistic exchange between biology and computer science (5, 6). The algorithmic lens has enhanced our understanding of biological phenomena such as how birds flock (7), how slime molds solve the shortest path problem (8–10), and how computation takes place in the brain (11, 12). Moreover, the process of evolution itself has been studied using an algorithmic lens (13–16). Also, inspiration from nature has led to algorithmic ideas such as ant-inspired algorithms for distributed density estimation (17), artificial neural networks in machine learning (18), and algorithms for similarity search inspired by the fruit fly brain (19), among others (20–23).

**Fig. 1.**
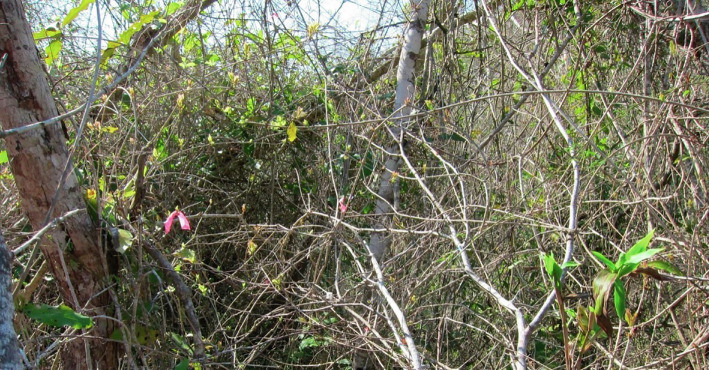
Tangled branches in which turtle ants forage.

Here, we investigate how the trail networks of the arboreal turtle ant (Cephalotes goniodontus) can solve variants of the shortest path problem, a basic optimization problem on graphs (24–26). Textbook algorithms for this problem find optimum solutions using knowledge of the entire network (27–29). Turtle ants nest and forage in the tree canopy of the tropical forest; their trail network is constrained to lie on a natural graph formed by tangled branches and vines (Fig. 1), and no ant has any global information about the network. Observations of turtle ants in the field show that a colony’s trail network approximately minimizes the number of vertices (30). We develop a model that gives a biologically plausible explanation for this observation and outlines other intriguing phenomena as described in the next section.

## A. Summary of Model and Results.

Turtle ant colonies form trails on a graph whose vertices correspond to junctions in the vegetation and edges correspond to branches connecting these junctions. A colony’s network of trails connects many nests and food sources. The trail network minimizes the number of vertices (30) (compared to simulated random networks), approximately solving a variant of the Steiner tree problem (31–33), which is a generalization of the shortest path problem for multiple terminal vertices. Here, we focus on a section of this network, considering two terminal vertices, such as a nest and a food source, and we seek to explain how a colony can find the path with the minimum number of vertices between the two terminals. We model trails as a bidirectional flow of ants between the two terminal vertices; bidirectional flow is characteristic of the trails of this species (34). The flow in our model is similar to models of flow in traffic networks (35–37). Ants lay trail pheromone on edges as they traverse them, and the next edge to traverse is chosen based on the level of the pheromone. The pheromone decays with time. Some fraction of flow leaks as it passes through each vertex, modeling the loss of ants due to exploration. Chandrasekhar et al. (30) hypothesized loss of ants at the vertices to be the reason why ants prefer paths with fewer vertices.

Our model leads to four main results:

1. We first consider the linear decision rule, which, at each vertex, divides the flow among the next set of edges in proportion to their pheromone level. We show through simulations and analysis that when the incoming rate of flow remains unchanged, the dynamics converges to the path with the minimum leakage. This is also the path with the minimum number of vertices when all vertices have equal leakage. This result describes a biologically plausible process that explains how colonies can find paths with the minimum number of vertices.
2. We show that when the rate of flow increases with time, in the absence of leakage, the dynamics converges to the shortest path. The flow rate on ant trails can change over time (38–42), for example, in turtle ants, the flow rate can increase in response to new food sources (39). In other ant species, it has been shown that ant trails converge to the shortest path in certain simple graphs (43). Our result shows a surprising link between these two phenomena: Ant colonies can use their ability to increase the flow rate to find the shortest path.
3. We establish the utility of bidirectional flow by showing that it is necessary for convergence to the shortest or the minimum leakage path. In contrast, most flow-based problems considered in computer science and operations research have unidirectional flow (37, 44–46).
4. We investigate the effect of increasing flow and leakage with decision rules other than the linear rule. For a general family of decision rules, we show that the linear rule is its unique member with guaranteed convergence to the shortest and the minimum leakage path. However, for various nonlinear rules, we show that the dynamics still often converges to a path with a smaller length and less leakage, compared to the path found in the absence of increasing flow and leakage respectively. Thus, the utility of increasing flow and leakage is not limited to the linear decision rule.

Our model builds on a previous model by Chandrasekhar et al. (47), adding components such as leakage and variation in the flow rate. These components were not present in the model of Chandrasekhar et al. (47) and are crucial for the phenomena we discuss above. Chandrasekhar et al. (47) investigated how ants find alternative paths, not necessarily the ones with the minimum number of vertices, to route around ruptured links in a network. Here, we ask how ants can find the path with the minimum number of vertices and how the flow rate impacts the path found. We demonstrate that leakage at vertices can lead to convergence to the path with the minimum number of vertices, and increase in the flow rate over time can lead to convergence to the shortest path.

Our work is different from traditional ant-colony optimization (48–50), in which the algorithms considered are not required to be biologically plausible. Models of ant colony optimization (ACO), inspired by ant behavior, solve combinatorial optimization problems, such as the traveling salesman problem (51) and the shortest path problem (52, 53). In ACO, individual agents, simulating ants, construct candidate solutions using heuristics and then use limited communication, simulating trail pheromone, to lead other agents toward better solutions. The simulated ants have significantly more computational power than is biologically plausible for real ants. Unlike real ants, the simulated ants have the ability to remember, retrace, and reinforce entire paths and can use the quality of the global solution to determine the amount of “pheromone” to be laid.

Our model resembles the reinforced random walks introduced by Diaconis et al. (54–56), which have found applications in biology (57–59). Here, a single agent traverses a graph by choosing edges with probability proportional to their edge weight, with edge weight here analogous to the level of trail pheromone. This edge weight increases additively each time the edge is traversed. However, there are a few key differences between the model we study and the setup for reinforced random walks: 1) Our model involves many agents, modeled by a flow, and their behavior is affected by their collective action in putting down pheromone, while the model for reinforced random walks considers a single agent whose behavior is influenced by its past random choices. 2) Pheromone decays geometrically over time in our model, but edge weights do not decrease in reinforced random walks. 3) Our model has leakage at each vertex, which is not present in the setup for reinforced random walks. Nevertheless, in a similar spirit to random walk-like processes studied before, the model we investigate is a Markov process of particular relevance to biology.

## 1. The Model

**Fig. 2.**
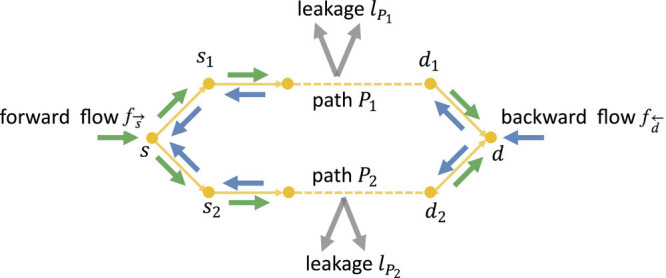
Forward and backward flow in a graph with parallel paths.

We consider bidirectional flow on a directed graph *G* = (*V*, *E*) with source vertex and destination vertex *s* and *d*, respectively. For each vertex *v*, let $f_{\vec{v}}\left(t\right)$ and $f_{\overset{\leftarrow }{v}}\left(t\right)$ denote the forward and backward flow on *v* present at time *t*, respectively. The forward flow moves along the direction of the edges, and the backward flow moves in the opposite direction (Fig. 2). Let *p*_*uv*_ (*t*) denote the pheromone level on edge (*u*, *v*) at time *t*. The pheromone level and flow together constitute the state of the system at any time *t*, which is updated as follows:

1. Flow movement: At each time step, the forward flow on a vertex moves along its outgoing edges, dividing itself based on a decision rule that depends on the pheromone levels on these edges. The simplest such rule divides the flow proportional to the pheromone level on the outgoing edges. Formally, let $f_{\vec{\mathrm{uv}}}\left(t\right)$ denote the forward flow moving along edge (*u*, *v*) at time *t*. Then, according to this rule, which we call the linear decision rule,
  [1] $$\begin{array}{c} f_{\vec{\mathrm{uv}}}\left(t\right)=f_{\vec{u}}\left(t\right)\frac{p_{\mathrm{uv}}\left(t\right)}{\sum_{z:(u,z)\in E}p_{\mathrm{uz}}\left(t\right)}. \end{array}$$
  The total forward flow on vertex *v* is the sum of flow along its incoming edges, multiplied by a leakage factor:
  [2] $$\begin{array}{c} f_{\vec{v}}\left(t+1\right)=\left(1-l_{v}\right)\sum_{z:(z,v)\in E}f_{\vec{\mathrm{zv}}}\left(t\right). \end{array}$$
  The leakage parameter *l*_*v*_ ∈ [0, 1] models the loss of ants due to exploration at each vertex. Movement for backward flow takes place in exactly the same manner, with the direction of the flow reversed. Formally, let $f_{\overset{\leftarrow }{\mathrm{uv}}}\left(t\right)$ denote the backward flow moving along the edge (*u*, *v*) at time *t*. Then,
  [3] $$\begin{array}{c} f_{\overset{\leftarrow }{\mathrm{uv}}}\left(t\right)=\;f_{\overset{\leftarrow }{v}}\left(t\right)\frac{p_{\mathrm{uv}}\left(t\right)}{\sum_{z:(z,v)\in E}p_{\mathrm{zv}}\left(t\right)}. \end{array}$$
  The total backward flow at *u* is the sum of backward flow along its outgoing edges, multiplied by the leakage parameter.
  [4] $$\begin{array}{c} f_{\overset{\leftarrow }{u}}\left(t+1\right)=\left(1-l_{u}\right)\sum_{z:(u,z)\in E}f_{\overset{\leftarrow }{\mathrm{uz}}}\left(t\right). \end{array}$$
  At each time *t*, new forward and backward flow, $f_{\vec{s}}\left(t\right)$ and $f_{\overset{\leftarrow }{d}}\left(t\right)$, appears on the source vertex and destination vertex *s* and *d*, respectively.
2. Pheromone update: At each time step, the pheromone level on an edge increases by the amount of flow on it and decays by a multiplicative factor of *δ* similar to (47):
  [5] $$\begin{array}{c} p_{\mathrm{uv}}\left(t+1\right)=\delta \left(p_{\mathrm{uv}}\left(t\right)+f_{\vec{\mathrm{uv}}}\left(t\right)+f_{\overset{\leftarrow }{\mathrm{uv}}}\left(t\right)\right). \end{array}$$
  Note that unlike flow, the pheromone level present on an edge does not have any direction and is influenced by flow from both the directions.

Definition 1. For any path *P* from source *s* to destination *d*, we define its leakage *l*_*P*_ as the fraction of flow that leaks out while moving through *P*, that is, *l*_*P*_ = 1 − *Π*_*v* ∈ *P* \ {*s*, *d*}_(1 − *l*_*v*_).^*^

### A. Discussion of Modeling Assumptions.

Our goal is to provide a simple and minimal biologically plausible model that explains how ants can find the path with the minimum number of vertices. We discuss our modeling assumptions below.

While we describe our model for unweighted graphs, it is general enough to capture the case with integral/rational edge lengths. For instance, an edge with length *k* can be represented using *k* unit length edges connected in series, with leakage at the vertices connecting these edges set to zero.

Our model uses a directed graph. Previous work shows that individual ants are unlikely to turn around on the trail, so that when an ant leaves a terminal, such as a nest or a food source, its distance from that terminal increases over time (39). To account for this, Chandrasekhar et al. (30) assigned direction to each edge relative to a terminal vertex, where the outbound direction goes away from the terminal vertex and the inbound direction goes toward it. Similarly, we use directed edges in our model.

In our model, the backward flow at the destination vertex is not dependent on the forward flow reaching it at the previous time step. This is because an ant does not necessarily make a round trip from one terminal to the other and back. The flow between the two terminal vertices in our model represents only a section of the larger network, which includes many nests and food sources. Ants reaching a terminal vertex can go on to other parts of the network instead of turning back.

## 2. Results

In this section, we discuss the convergence properties for the model defined above. We defer all the proofs and simulation details to *SI Appendix*.

### A. Convergence to the Minimum Leakage Path and the Shortest Path.

#### A.1. Constant flow with time.

**Fig. 3.**
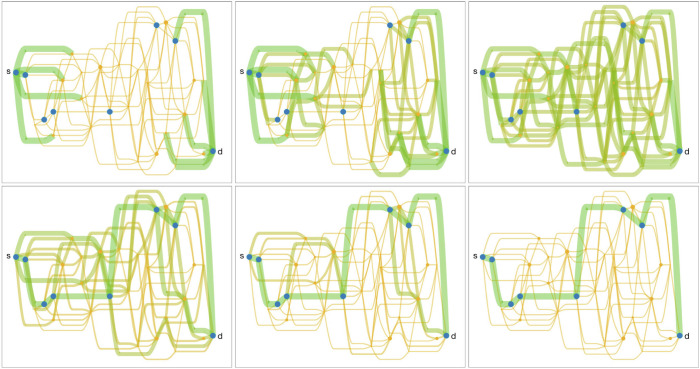
Flow dynamics governed by the linear decision rule converges to the path with the minimum leakage (shown by blue vertices) when the incoming flow does not change with time. Larger dots represent vertices with smaller leakage, and thickness of the green edges corresponds to the flow level.

Through simulations and analysis, we show that when the incoming forward and backward flow does not change with time, with the linear decision rule, the dynamics converges to the path with the minimum leakage (Fig. 3).

We run simulations for three families of directed graphs:

1. *G* (*n*, *p*): The *G* (*n*, *p*) model is a widely used random graph model which consists of graphs on *n* vertices where each pair of vertices has an edge with probability *p*.
2. *G* (*n*, *p*) with a locality constraint: The standard *G* (*n*, *p*) model allows edges between any two vertices (with probability *p*). However, the graphs formed by branches and vines in the natural vegetation have a local physical structure in which the edges are more likely between nearby vertices. To capture this, we consider the *G* (*n*, *p*) model with an additional locality constraint that an edge exists between vertex *i* and *j* only if |*i* − *j*| ≤ *k* for some parameter *k*. Here, the vertices are labeled from 1 to *n* with the source and the destination vertex labeled 1 and *n*, respectively.
3. *n* × *n* grid graph.

We generate a large number of instances with different values for *n*, *p*, *k*, and other parameters and observe convergence to the minimum leakage path in all the simulated instances. More details about the simulations can be found in *SI Appendix*, Appendix C.

We complement our simulations on general graph models with a provable convergence result for graphs with two parallel paths, a case that has been experimentally investigated in the past (43, 60).

Theorem 1. *Consider a graph G consisting of two parallel paths P_1_ and P_2_ from s to d. Let the flow and the pheromone levels be updated according to the model in Section 1, and let P_1_ be the path with the minimum leakage. If i) the incoming flow values* $f_{\vec{s}}\left(t\right)$ *and* $f_{\overset{\leftarrow }{d}}\left(t\right)$ *are nonzero and unchanging with time, and ii) the initial pheromone level p_uv_ (0) is positive for all edges (u, v) ∈ P_1_, then the flow dynamics governed by the linear decision rule converges to a state where all the flow goes through P_1_.*

At a high level, the proof involves showing that relatively more pheromone accumulates on the path with less leakage as time progresses. Although the update rules governing our model are simple, mathematically understanding its dynamics is surprisingly nontrivial. Even for the seemingly simple case of two parallel paths, the progression of pheromone levels can be highly nonmonotone, and the proof needs a careful construction of an appropriate potential function. We give a sketch of the proof in Section 3.

To connect this result to the observation by Chandrasekhar et al. (30) that ants form trails with approximately the minimum number of vertices, we need to connect leakage to the number of vertices. Note that the path with minimum leakage is also the path with the minimum number of vertices when all the vertices have equal leakage. Moreover, we can show that this connection between leakage and the number of vertices degrades gracefully, and as long as the variation in leakage between different vertices is not too large, the path with the minimum leakage has approximately the minimum number of vertices. One way to formalize this is to assume that for any pair of vertices *u* and *v*, log(1 − *l*_*u*_) and log(1 − *l*_*v*_) are within a (1 + *ϵ*) factor of each other. Then, we can show that the path with the minimum leakage has number of vertices at most (1 + *ϵ*) times the path with the minimum number of vertices (*SI Appendix*, Appendix A.3 for the proof). Thus, our result on convergence to the minimum leakage path suggests a plausible way in which ants can converge to the path with approximately the minimum number of vertices. In the case when there is a large variation in leakage between different vertices, the path with the minimum leakage may not have approximately the minimum number of vertices. However, even in this case, convergence to the minimum leakage path is consistent with the hypothesis of Chandrasekhar et al. (30) that turtle ants prefer paths that minimize their chances of getting lost.

#### A.2. Increasing flow with time.

For the previous result, we assumed that the incoming flow does not change with time. Now, we consider the effect of change in flow. We show that if the incoming forward and backward flow increases with time, in the absence of leakage, the dynamics governed by the linear decision rule converges to the shortest path.

We run the simulations for the same families of graphs considered for the last result. The differences in these sets of simulations are the absence of leakage, and the incoming flow increases by a fixed factor in each step. We generate a large number of graph instances with different values of the parameters and observe convergence to the shortest path in all the simulated instances.

We also show provable convergence to the shortest path for graphs consisting of two parallel paths.

Theorem 2. *Consider a graph G consisting of two parallel paths P_1_ and P_2_ from s to d. Let the flow and the pheromone levels be updated according to the model in Section 1 and let P_1_ be the shorter path. If i) the initial pheromone level p_uv_ (0) is positive for all edges (u, v) ∈ P_1_, ii) leakage l_P_1__ = l_P_2__ = 0, and iii) the incoming flow increases as follows:*

1. *Multiplicative increase:* $f_{\vec{s}}\left(t\right)=\alpha^{t}f_{\vec{s}}\left(0\right)$ *and* $f_{\vec{d}}\left(t\right)=\alpha^{t}f_{\vec{d}}\left(0\right)$*, for any *α* > 1, or*
2. *Additive increase:* $f_{\vec{s}}\left(t\right)=f_{\vec{s}}\left(0\right)+\alpha t$ *and* $f_{\vec{d}}\left(t\right)=f_{\vec{d}}\left(0\right)+\alpha t$*, for any α > 0,*

*then the flow dynamics governed by the linear decision rule converges to a state where all the flow goes through P_1_.*

For ease of analysis, we consider only the cases when the flow increases by a fixed additive or multiplicative factor. Our analysis suggests that the outcome may be similar when the rate of increase is not fixed; further work is needed to demonstrate this.

For an intuitive explanation of this result, consider a graph with two parallel paths as shown in Fig. 2 such that path *P*_1_ is shorter than *P*_2_, and there is no leakage. Consider the forward flow on edges (*d*_1_, *d*) and (*d*_2_, *d*). Since *P*_1_ is shorter, the forward flow on (*d*_1_, *d*) corresponds to the more recent forward flow that entered from *s* compared to the forward flow on (*d*_2_, *d*). Since the flow is increasing with time, more recent flow is larger, ensuring that relatively more pheromone accumulates on (*d*_1_, *d*) than (*d*_2_, *d*) as time progresses. Similarly, relatively more pheromone accumulates on (*s*, *s*_1_) than (*s*, *s*_2_) as time progresses due to the increasing backward flow. Thus, as time progresses, relatively more pheromone accumulates on *P*_1_. However, as in the case with leakage, an increase in relative pheromone levels on *P*_1_ is not monotone, and we require a more careful proof. We give a sketch of the proof in Section 3.

Previous studies have shown that ants are capable of finding the shortest path in certain simple graphs (43) and that factors such as detection of new food sources can positively reinforce the rate of flow of ants (38, 39). Our result shows a surprising connection between these two phenomena.

The main goal of our work is to demonstrate the intriguing connection between leakage and flow rate and the shortest path problem. We do this using simulations on general graph models and analysis on graphs with parallel paths. Based on our simulations, we conjecture that the above results showing provable convergence hold for general graphs.

Conjecture 1. *The provable convergence results in Theorems 1 and 2 hold for general graphs.*

We can view leakage and increasing flow as two possibly conflicting forces, leading to paths with minimum leakage and length, respectively. An interesting direction for future work would be to investigate the dynamics when both these forces are active simultaneously.

### B. General Rules and Fundamental Limits.

In the previous subsection, we show that bidirectional flow with the linear decision rule converges to the path with the minimum leakage when the flow is fixed and to the shortest path when the flow is increasing and there is no leakage. How crucial is the bidirectional nature of the flow? How does the dynamics behave when the decision rule is nonlinear? Next, we study these questions.

#### B.1. Necessity of bidirectional flow.

We show that bidirectional flow is necessary to find the shortest or the minimum leakage path. This result holds independent of the decision rule, leakage levels, and the change in the flow rate; we formally state this result next. Let 𝒢 be the set of all decision rules that distribute the forward (backward) flow at any vertex, onto the outgoing (incoming) edges only based on their pheromone levels.

Theorem 3 *Consider any graph G with two parallel paths between s and d. Let there be unidirectional flow from s to d. For any decision rule in 𝒢, any setting of leakage parameters and with arbitrary incoming flow levels, there exists a setting of initial pheromone levels such that the dynamics does not converge to the shortest or the minimum leakage path.*

Thus, bidirectional flow is necessary for all pheromone-based rules to guarantee convergence to the shortest or the minimum leakage path. The main idea behind this result is that with only unidirectional flow from *s* to *d*, pheromone on edges incident on *s* can not encode information about the rest of the graph.

#### B.2. The conflict between leakage, change in flow, and nonlinearity.

To understand the dynamics for other decision rules beyond the linear rule, we consider a family of decision rules ℱ satisfying certain assumptions. This family is a subset of the family 𝒢 discussed above. These rules distribute the forward flow among the outgoing edges of any vertex and backward flow among the incoming edges based on the normalized pheromone levels at these edges (as defined below). That is, allocation of flow does not depend on the absolute pheromone levels. We also assume that these rules are monotone in the sense that increasing the normalized pheromone level at any edge does not decrease the proportion of flow entering that edge. Most decision rules considered in the past (47) satisfy these conditions.

Here, the normalized pheromone level is defined as follows:

[6] $$\begin{array}{c} {\bar{p}}_{\vec{\mathrm{uv}}}\left(t\right)=\frac{p_{\mathrm{uv}}\left(t\right)}{\sum_{z:(u,z)\in E}p_{\mathrm{uz}}\left(t\right)}\;\text{,}\;{\bar{p}}_{\overset{\leftarrow }{\mathrm{uv}}}\left(t\right)=\frac{p_{\mathrm{uv}}\left(t\right)}{\sum_{z:(z,v)\in E}p_{\mathrm{zv}}\left(t\right)}. \end{array}$$

Note that while the pheromone level on an edge has no associated direction, there is a forward and backward normalized pheromone level for each edge depending on whether the normalization is done with respect to the incoming edges or the outgoing edges.

More formally, we consider the family of rules given by functions *g* : [0, 1/2]→[0, 1] ∈ ℱ. For any vertex with out-degree (in-degree) 2, a rule in this family uses a function *g*, which takes as input the minimum of the normalized pheromone levels on the two outgoing (incoming) edges and returns the fraction of the forward (backward) flow on this edge. In other words, *g*(*x*) is the fraction of flow sent on edge with normalized pheromone level *x*, for *x* < 0.5. We assume that *g* is monotonically increasing, that is, *g*(*x*) ≤ *g*(*x*′) for all *x* ≤ *x*′. Furthermore, *g* satisfies *g*(0) = 0 and *g*(1/2)=1/2. This condition says that if one of the edges has 0 normalized pheromone, there is no flow on it, and if both the edges have equal pheromone levels, there is equal flow on them. For any vertex with out-degree (in-degree) 1, the forward (backward) flow goes to the next (previous) vertex, just as in the case of the linear rule. For our results below, we need to define these rules for only degree 1 and 2 vertices.

Note that the linear decision rule studied in the previous subsection belongs to ℱ and corresponds to *g*(*x*) = *x*.

We study the dynamics when the decision rule belongs to the family of decision rules defined above and is nonlinear. We show that for every nonlinear rule *g* ∈ ℱ, there exists a setting of leakage parameters in which the dynamics fails to converge to the path with the minimum leakage.

Theorem 4. *Consider any graph G consisting of two parallel paths from s to d. When the incoming flow is fixed, for every nonlinear decision rule g ∈ ℱ, there exists a setting of leakage parameters and initial pheromone and flow levels dependent on g, such that the dynamics does not converge to the path with the minimum leakage.*

We show an analogous result for the increasing flow case with no leakage.

Theorem 5. *Consider any graph G consisting of two parallel paths from s to d with a unique shortest path. When the leakage is zero for all the vertices, for every nonlinear decision rule g ∈ ℱ, there exists a setting of initial pheromone and flow levels, with incoming flow increasing by a fixed multiplicative factor at each time step, such that the dynamics does not converge to the shortest path. The multiplicative factor and initial pheromone and flow levels are chosen as a function of g.*

For an intuitive explanation for these results, consider the quadratic decision rule that distributes the flow in proportion to the square of the pheromone levels. Due to the square in the quadratic decision rule, given two edges incident on a vertex, this rule sends more than linearly proportional flow on the edge with the higher pheromone. Thus, if a path—that is not necessarily the shortest or the minimum leakage path—has relatively high pheromone initially, even more pheromone accumulates on it as time progresses, and the dynamics may not converge to the shortest or the minimum leakage path. Theorems 4 and 5 formalize this intuition for any nonlinear decision rule belonging to the family ℱ.

From the last subsection, we can view increasing flow and leakage as two conflicting forces, preferring the shortest and the minimum leakage path, respectively. The above results suggest that nonlinearity in the decision rule can be viewed as another force, in conflict with the forces of leakage and increasing flow, preferring certain states that may not correspond to the shortest or the minimum leakage path.

#### B.3. Usefulness of increasing flow and leakage not limited to the linear decision rule.

Note that the above results only suggest that the linear decision rule is necessary for guaranteed convergence to the shortest or the minimum leakage path. Can it be the case that even with some nonlinear decision rules, the forces of increasing flow and leakage still help in finding shorter or smaller leakage paths, respectively, compared to the paths found in the absence of these forces? To understand this question, we ran simulations for various nonlinear decision rules, some of which have been previously used to model ant behavior (47, 61). We observe that within each graph family, for a large fraction of the graph instances, the path found in the presence of these forces has length (respectively leakage) smaller than or equal to the length (respectively leakage) of the path found in their absence.

**Fig. 4.**
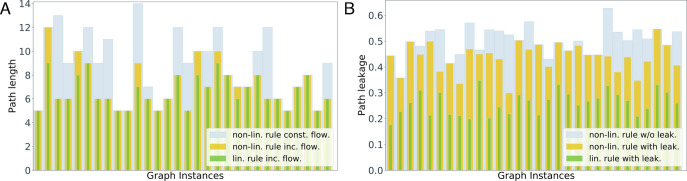
Effect of leakage and increasing flow with the quadratic decision rule which is nonlinear. (*A*) Path length obtained with the quadratic decision rule with and without increasing flow and the linear decision rule with increasing flow. (*B*) Path leakage obtained with the quadratic decision rule with and without leakage present at vertices, and the linear decision rule with leakage present.

Fig. 4 demonstrates this for the quadratic decision rule for *G*(*n*, *p*) graphs with the locality constraint. This rule divides the flow in proportion to the square of the pheromone levels.

In Fig. 4*A*, we show the path length obtained by the quadratic decision rule with and without an increase in flow and by the linear rule with an increase in flow, for 30 random graph instances. As discussed before, the linear rule finds the shortest path. But even with the quadratic rule, the path length obtained in the presence of increasing flow is smaller than or equal to the length obtained in its absence.

Similarly, Fig. 4*B* shows the path leakage obtained by the quadratic decision rule with and without leakage present at the vertices and by the linear decision rule with leakage present. There is a subtle distinction here between path leakage as an objective function and leakage as a process affecting the dynamics. For each graph instance, we assign leakage values to vertices (*SI Appendix*, Appendix C for details). This gives us a path leakage objective function which we measure in all the three cases. However, in the case of the quadratic rule without leakage, the leakage process is not applied at vertices during the dynamics. This gives a baseline to which we compare the path leakage objective when the leakage process is applied. We observe that the quadratic rule with leakage applied leads to path leakage objective smaller than or equal to the baseline, while the linear rule with leakage applied minimizes the objective as discussed in Section 2.A.

We observe similar results for other graph families and nonlinear decision rules. However, the extent to which the forces of increasing flow and leakage are effective varies with the nonlinear decision rule and graph family. For instance, we observe that compared to the quadratic rule, these forces are more effective for a nonlinear rule closer to the linear rule, dividing the flow in proportion to the 1.1th power of the pheromone levels. Also, for most graph families and nonlinear decision rules considered, there is a small fraction of instances where these forces end up increasing the path length (respectively path leakage). Nonetheless, for all the graph families and nonlinear decision rules considered, for most (> 80%) graph instances, the path found in the presence of these forces has length (respectively leakage) smaller than or equal to the length (respectively leakage) of the path found in their absence. Thus, the usefulness of the forces of leakage and increasing flow is not limited to the linear decision rule. We include more details and discussion of these simulations in *SI Appendix*, Appendix C.

## 3. Proof Ideas

### A. Linear Decision Rule with Increasing Flow.

For a graph consisting of parallel paths *P*_1_ and *P*_2_, with *len*_*P*_1__ < *len*_*P*_2__ (Fig. 2), Theorem 2 says that the dynamics converges to *P*_1_ when the incoming flow increases multiplicatively or additively with time. To prove this, we show that as time progresses, relatively more pheromone is accumulated on *P*_1_ than *P*_2_.

Let *s*_1_ and *s*_2_ be the neighboring vertices of *s*, and *d*_1_ and *d*_2_ be the neighboring vertices of *d*, on path *P*_1_ and *P*_2_, respectively. Note that only the pheromone level on edges (*s*, *s*_1_), (*s*, *s*_2_), (*d*_1_, *d*), (*d*_2_, *d*) affects the dynamics for this graph. Consider the ratio of pheromone levels on (*s*, *s*_1_) and (*s*, *s*_2_) at time (*t* + 1):


[7] $$\begin{array}{l} \begin{array}{l} \begin{array}{ll} \frac{p_{ss_{1}}(t+1)}{p_{ss_{2}}(t+1)} & =\frac{\delta (p_{ss_{1}}(t)+\;f_{\vec{ss_{1}}}(t)+\;f_{\overset{\leftarrow }{ss_{1}}}(t))}{\delta (p_{ss_{2}}(t)+\;f_{\vec{ss_{2}}}(t)+\;f_{\overset{\leftarrow }{ss_{2}}}(t))}. \end{array} \end{array} \end{array}$$

[8] $$\begin{array}{ll} & =\frac{p_{ss_{1}}(t)+\;f_{\vec{ss_{1}}}(t)+(1-l_{P_{1}})f_{\overset{\leftarrow }{d_{1}d}}(t-len_{P_{1}}+1)}{p_{ss_{2}}(t)+\;f_{\vec{ss_{2}}}(t)+(1-l_{P_{2}})f_{\overset{\leftarrow }{d_{2}d}}(t-len_{P_{2}}+1)}. \end{array}$$

[9] $$\begin{array}{ll} & =\frac{p_{ss_{1}}(t)+\;f_{\vec{ss_{1}}}(t)+\;f_{\overset{\leftarrow }{d}}(t-len_{P_{1}}+1)\;{\bar{p}}_{\overset{\leftarrow }{d_{1}d}}(t-len_{P_{1}}+1)}{p_{ss_{2}}(t)+\;f_{\vec{ss_{2}}}(t)+\;f_{\overset{\leftarrow }{d}}(t-len_{P_{2}}+1){\bar{p}}_{\overset{\leftarrow }{d_{2}d}}(t-len_{P_{2}}+1)}, \end{array}$$

where in the last equation, we set leakage *l*_*P*_1__ = *l*_*P*_2__ = 0, and write the backward flow in terms of the normalized pheromone level. For simplicity, let us assume $f_{\overset{\leftarrow }{d}}\left(t\right)=\alpha^{t}$, for some *α* > 1.

This gives


[10] $$\begin{array}{ll} \frac{p_{ss_{1}}(t+1)}{p_{ss_{2}}(t+1)} & =\frac{p_{ss_{1}}(t)+\;f_{\vec{ss_{1}}}(t)+\alpha^{\left(t-len_{P_{1}}+1\right)}\;{\bar{p}}_{\overset{\leftarrow }{d_{1}d}}(t-len_{P_{1}}+1)}{p_{ss_{2}}(t)+\;f_{\vec{ss_{2}}}(t)+\alpha^{\left(t-len_{P_{2}}+1\right)}{\bar{p}}_{\overset{\leftarrow }{d_{2}d}}(t-len_{P_{2}}+1)}. \end{array}$$

As *len*_*P*_1__ < *len*_*P*_2__, we know that *α*^ (*t* − *len*_*P*_1__ + 1)^ > *α*^ (*t* − *len*_*P*_2__ + 1)^. These backward flow terms, *α*^(*t* − *len*_*P*_1__ + 1)^ and *α*^ (*t* − *len*_*P*_2__ + 1)^, are the main reason why relatively more pheromone accumulates on *ss*_1_ compared to *ss*_2_ as time progresses. However, the ratio $\frac{p_{ss_{1}}\left(t\right)}{p_{ss_{2}}\left(t\right)}$ may not increase monotonically at each time step. To circumvent this issue, we carefully construct a potential function which increases monotonically with time. Our potential function is given by the minimum of the ratio of the pheromone levels $\frac{p_{ss_{1}}\left(t\right)}{p_{ss_{2}}\left(t\right)}$ and $\frac{p_{d_{1}d}\left(t\right)}{p_{d_{2}d}\left(t\right)}$ across the last *max*(*len*_*P*_1__, *len*_*P*_2__) time steps. Let $r_{ss_{1}}\left(t\right)\overset{\;\text{def}}{=}\frac{p_{ss_{1}}\left(t\right)}{p_{ss_{2}}\left(t\right)}$, $r_{d_{1}d}\left(t\right)\overset{\;\text{def}}{=}\frac{p_{d_{1}d}\left(t\right)}{p_{d_{2}d}\left(t\right)}$, and $L\overset{\;\text{def}}{=}max\left(len_{P_{1}},len_{P_{2}}\right)$. Our potential function is given by

$$\begin{array}{cc} r_{\min}\left(t\right) & \overset{\mathrm{def}}{=}min\{r_{ss_{1}}\left(t\right),r_{ss_{1}}\left(t-1\right),\cdots ,r_{ss_{1}}\left(t-L+1\right),\\ & \;r_{d_{1}d}\left(t\right),r_{d_{1}d}\left(t-1\right),\cdots ,r_{d_{1}d}\left(t-L+1\right)\}. \end{array}$$

Using the definition of the linear decision rule, we know that $\frac{f_{\vec{ss_{1}}}\left(t\right)}{f_{\vec{ss_{2}}}\left(t\right)}=\frac{p_{ss_{1}}\left(t\right)}{p_{ss_{2}}\left(t\right)}=r_{ss_{1}}\left(t\right)\geq r_{\min}\left(t\right)$. Furthermore, it can be shown that ${\bar{p}}_{\overset{\leftarrow }{d_{1}d}}\left(t-len_{P_{1}}+1\right)\geq \frac{r_{\min}\left(t\right)}{1+r_{\min}\left(t\right)}$, and ${\bar{p}}_{\overset{\leftarrow }{d_{2}d}}\left(t-len_{P_{2}}+1\right)\leq \frac{1}{1+r_{\min}\left(t\right)}$, which implies that $\frac{{\bar{p}}_{\overset{\leftarrow }{d_{1}d}}\left(t-len_{P_{1}}+1\right)}{{\bar{p}}_{\overset{\leftarrow }{d_{2}d}}\left(t-len_{P_{2}}+1\right)}\geq r_{\min}\left(t\right)$.

These inequalities give us

[11] $$\begin{array}{cc} & \frac{p_{ss_{1}}\left(t+1\right)}{p_{ss_{2}}\left(t+1\right)}\\ & \;\geq r_{\min}\left(t\right)\frac{p_{ss_{2}}\left(t\right)+f_{\vec{ss_{2}}}\left(t\right)+\alpha^{\left(t-len_{P_{1}}+1\right)}{\bar{p}}_{\overset{\leftarrow }{d_{2}d}}\left(t-len_{P_{2}}+1\right)}{p_{ss_{2}}\left(t\right)+f_{\vec{ss_{2}}}\left(t\right)+\alpha^{\left(t-len_{P_{2}}+1\right)}{\bar{p}}_{\overset{\leftarrow }{d_{2}d}}\left(t-len_{P_{2}}+1\right)}\\ & \;>r_{\min}\left(t\right), \end{array}$$

where we used *α*^ (*t* − *len*_*P*_1__ + 1)^ > *α*^ (*t* − *len*_*P*_2__ + 1)^ for the last inequality. This gives us $\frac{p_{ss_{1}}\left(t+1\right)}{p_{ss_{2}}\left(t+1\right)}=r_{ss_{1}}\left(t+1\right)>r_{\min}\left(t\right)$. Thus, the backward flow terms *α*^ (*t* − *len*_*P*_1__ + 1)^ and *α*^ (*t* − *len*_*P*_2__ + 1)^ ensure that the pheromone ratio at the edges incident on *s* at time *t* + 1 is greater than *r*_*min*_ (*t*), the minimum of the pheromone ratios at the edges incident on *s* and *d* across last *L* time steps. Similarly, the forward flow ensures *r*_*d*_1_*d*_(*t* + 1) > *r*_*min*_(*t*). This implies that *r*_*min*_(*t*) never decreases and strictly increases every *L* time steps. We use this to show convergence to the shortest path.

The proof for the case involving leakage with fixed flow (Theorem 1) is similar and uses the same potential function. The only difference is that in this case, the potential function goes up due to the leakage terms 1 − *l*_*P*_1__ and 1 − *l*_*P*_2__ (Eq. **8**), instead of the *α*^(*t* − *len*_*P*_1__ + 1)^ and *α*^(*t* − *len*_*P*_2__ + 1)^ terms.

### B. General Rules and Fundamental Limits.

In Theorem 3, we claim that for any pheromone-based rule, bidirectional flow is necessary for convergence to the shortest or the minimum leakage path. When there is unidirectional flow from *s* to *d*, the pheromone levels on the edges incident on *s* are only a function of their initial pheromone levels and forward flow at *s*. It does not depend on the flow and pheromone levels on the rest of the graph. Therefore, in the case of two parallel paths, for a given decision rule and initial setting of the pheromone levels, if the dynamics converges to a particular path, then they will converge to the other path if we swap the initial pheromone levels on the two edges incident on *s*. Hence, we can always set the initial pheromone levels on the edges incident on *s*, such that the dynamics does not converge to the shortest or the minimum leakage path.

Next, we provide a proof sketch for Theorem 4. The proof sketch for Theorem 5 is similar. Let *s*_1_ and *s*_2_ be the neighboring vertices of *s*, and *d*_1_ and *d*_2_ be the neighboring vertices of *d*, on path *P*_1_ and *P*_2_, respectively (Fig. 2). Let *P*_1_ be the minimum leakage path. We would show that the dynamics is not guaranteed to converge to path *P*_1_ for nonlinear *g* ∈ ℱ. For any nonlinear *g* ∈ ℱ, consider some *r* ∈ (0, 1/2) such that *g*(*r*)≠*r*. Such an *r* exists because *g* is nonlinear. Consider the two cases: 1) *g*(*r*) < *r*, 2) *g*(*r*) > *r*.

Suppose *g* is such that *g*(*r*) < *r*. Consider an instance where initial normalized pheromone levels ${\bar{p}}_{\vec{ss_{1}}}\left(0\right)$ and ${\bar{p}}_{\overset{\leftarrow }{d_{1}d}}\left(0\right)$ are at most *r*; the initial flow values on the edges of path *P*_1_ and *P*_2_ are at most *r* and at least 1 − *r*, respectively. Let the incoming forward and backward flow be equal to 1 at all times. The decision rule *g* sends at most *r* − *c*_*g*, *r*_ amount of forward and backward flow on path *P*_1_ (because *g*(*r*) < *r* and *g* is monotone) and at least 1 − *r* + *c*_*g*, *r*_ on path *P*_2_, where *c*_*g*, *r*_ is a positive constant dependent on *g* and *r*. The positive constant *c*_*g*, *r*_ ensures that we can set the leakage levels to be small enough, satisfying *l*_*P*_1__ < *l*_*P*_2__, such that even after leakage, the flow levels on *P*_1_ and *P*_2_ remain at most *r* and at least 1 − *r*, respectively, throughout the future. And normalized pheromone levels ${\bar{p}}_{\vec{ss_{1}}}$ and ${\bar{p}}_{\overset{\leftarrow }{d_{1}d}}$ remain at most *r*.

Using a similar idea, in the case when *g*(*r*) > *r*, we can set the initial flow and pheromone levels and the leakage parameters, such that the normalized pheromone levels ${\bar{p}}_{\vec{ss_{2}}}$, ${\bar{p}}_{\overset{\leftarrow }{d_{2}d}}$ and the flow levels on *P*_2_ never fall below *r* and the flow levels on *P*_1_ remain at most 1 − *r*. Thus, the dynamics never converges to the minimum leakage path *P*_1_.

## 4. Discussion

Like ant colonies, engineered systems such as molecular robots and swarm computing (62–66) involve a large population of individuals lacking central control and equipped with minimal computational resources. Searching for a target (67, 68), and in particular, finding the shortest path (69), is a basic task for such systems. Our algorithms based on leakage and increasing flow can also be applied to such swarms of robots equipped with the ability to release and detect pheromone (70–74), to solve the shortest path problem and its variants.

Our result on convergence to the shortest path suggests that an ant colony has the ability to discover the shortest path merely by increasing its flow rate. An interesting direction for future research would be to empirically investigate the relationship between the flow rate of ants and path length and understand whether ant colonies increase flow rates to find short paths.

Our results also open up avenues for further theoretical investigation. While the algorithms designed by humans are often set up so as to be amenable to analysis, nature is not constrained in this way. For seemingly simple models of biological systems, it has thus been notoriously difficult to devise mathematical guarantees on the quality of the solutions produced (6, 9, 75–81). In our model, analyzing the dynamics is challenging as it involves understanding the progression of pheromone level with time, which is affected by the actions of a large number of agents (modeled by flow), and can be highly nonmonotone even for the simple case of graphs with parallel paths. The behavior of this model in extensive simulations suggests that it should be possible to significantly generalize the results we prove here. In particular, we conjecture that provable convergence to the shortest or the minimum leakage path holds for general graphs (Conjecture 1). Another direction for future research is to extend our analysis to the case when multiple terminal vertices are present in the graph, as trail networks in nature usually include many nests and food sources.

In summary, our model for how ant trails change over time contributes to the synergistic exchange between biology and computer science, providing a plausible explanation for how turtle ant colonies can find paths that minimize the number of vertices, and suggesting a surprising algorithm for the shortest path discovery, by increasing the flow rate, applicable to distributed engineering systems.

## Supplementary Material

Appendix 01 (PDF)

## Data Availability

The code is available in Github (https://github.com/shivamg13/Arboreal-Ants).

## Footnotes

^*^For convenience, we overload notation, and use *v* ∈ *P* and (*u*, *v*) *P* to denote the vertices and edges present on path *P*, respectively.
