# Supplementary material for: Distributed algorithms from arboreal ants for the shortest path problem
Source: Proc Natl Acad Sci U S A. 2023 Jan 30;120(6):e2207959120. doi: 10.1073/pnas.2207959120 (PMC9963535; doi:10.1073/pnas.2207959120)
Supplement: Supplementary file 1 — Appendix 01 (PDF) [file pnas.2207959120.sapp.pdf]

Supplemental Material: Distributed Algorithms from Arboreal Ants for the Shortest Path Problem

Shivam Garg[†*1], Kirankumar Shiragur[†2], Deborah M. Gordon [3], Moses Charikar[1]

[1] Department of Computer Science, Stanford University, Stanford, CA 94305
[2] Department of Management Science and Engineering, Stanford University, Stanford, CA 94305
[3] Department of Biology, Stanford University, Stanford, CA 94305

[*]To whom correspondence should be addressed; Email: shivamgarg@stanford.edu
[†] These authors contributed equally

A Proof of Convergence

Here we provide proof for all the results stated in Section 3.1.

A.1 Fixed flow with different leakage on each path (Theorem 1)

Here, we provide a proof of Theorem 1. We restate it below.

Theorem 1. *Consider a graph G consisting of two parallel paths P_1 and P_2 from s to d. Let the flow and the pheromone levels be updated according to the model in Section 2, and let P_1 be the path with the minimum leakage. If (i) the incoming flow values $f_{\vec{s}}(t)$ and $f_{\overleftarrow{d}}(t)$ are non-zero and unchanging with time, and (ii) the initial pheromone level $p_{uv}(0)$ is positive for all edges $(u, v) \in P_1$, then the flow dynamics governed by the linear decision rule converges to a state where all the flow goes through P_1.*

Let s_1, s_2 be the neighboring vertices of s that belong to paths P_1 and P_2 respectively. Similarly let d_1 and d_2 be the corresponding neighbors for d. Let $f_{\vec{s}}, f_{\overleftarrow{d}} > 0$ be some fixed forward and backward flow values, such that $f_{\vec{s}}(t) = f_{\vec{s}}$ and $f_{\overleftarrow{d}}(t) = f_{\overleftarrow{d}}$ for all t. Define $r_{ss_1}(t) \stackrel{\text{def}}{=} \frac{p_{ss_1}(t)}{p_{ss_2}(t)}$ and $r_{d_1d}(t) \stackrel{\text{def}}{=} \frac{p_{d_1d}(t)}{p_{d_2d}(t)}$ to be the relative pheromone levels at (s, s_1) and (d_1, d) respectively. For notational simplicity, we define $m \stackrel{\text{def}}{=} len_{P_1}$ and $n \stackrel{\text{def}}{=} len_{P_2}$ to be the lengths of path P_1 and P_2, and $L \stackrel{\text{def}}{=} max(m, n)$. We also define $\alpha \stackrel{\text{def}}{=} 1 - l_{P_1}$ and $\beta \stackrel{\text{def}}{=} 1 - l_{P_2}$.

Our potential function at any time $t \geq L$ is given by the minimum of the relative pheromone levels $r_{ss_1}(t)$ and $r_{d_1d}(t)$ across the last L time steps:

$$r_{min}(t) \stackrel{\text{def}}{=} min\{r_{ss_1}(t), r_{ss_1}(t-1), \cdots, r_{ss_1}(t-L+1), r_{d_1d}(t), r_{d_1d}(t-1), \cdots, r_{d_1d}(t-L+1)\} \quad (12)$$

We divide our proof into 3 steps:

- **Step 1**: In this step, we show that $r_{min}(t)$ is non-decreasing at every time step and increases by a factor of $\gamma(t)$ every L time steps, for all $t \geq L$. Here, $\gamma(t)$ is some appropriately defined function which is greater than 1 for all $t \geq L$.
- **Step 2**: In this step, we will give a lower bound $\gamma_l > 1$, on $\gamma(t)$, to show that $r_{min}(t)$ increases sufficiently every L time steps.
- **Step 3**: We will find the rate of convergence based on the rate of increase shown in step 2.

Now, we give proofs for each of these three steps.

Step 1. We will use the following lemma for the proof.

Lemma 1. *For any time $t \geq L$, the following is true,*

$$\frac{f_{\overleftarrow{d_1d}}(t-m+1)}{f_{\overleftarrow{d_2d}}(t-n+1)} \geq \min(r_{d_1d}(t-m+1), r_{d_1d}(t-n+1)) \geq r_{min}(t) .$$

Proof. Under the linear decision rule we know that,

$$f_{\overleftarrow{d_1 d}}(t-m+1) = \frac{f_{\overleftarrow{d}}\, r_{d_1 d}(t-m+1)}{r_{d_1 d}(t-m+1)+1} \tag{13}$$

$$f_{\overleftarrow{d_2 d}}(t-n+1) = \frac{f_{\overleftarrow{d}}}{r_{d_1 d}(t-n+1)+1} \tag{14}$$

Let $r \stackrel{\text{def}}{=} \min(r_{d_1 d}(t-m+1), r_{d_1 d}(t-n+1))$, then note that $\frac{r_{d_1 d}(t-m+1)}{r_{d_1 d}(t-m+1)+1} \geq \frac{r}{r+1}$ and $\frac{1}{r_{d_1 d}(t-n+1)+1} \leq \frac{1}{r+1}$. Therefore,

$$\frac{f_{\overleftarrow{d_1 d}}(t-m+1)}{f_{\overleftarrow{d_2 d}}(t-n+1)} \geq r = \min(r_{d_1 d}(t-m+1), r_{d_1 d}(t-n+1)) \; .$$

and from the definition of $r_{min}(t)$, we know that $\min(r_{d_1 d}(t-m+1), r_{d_1 d}(t-n+1)) \geq r_{min}(t)$. □

Now we show that $r_{min}(t)$ is non-decreasing at every time step and increases by a factor of $\gamma(t)$ every L time steps, for all $t \geq L$. To show this, we would show that $r_{ss_1}(t+1) \geq r_{min}(t)\gamma_s(t)$, and $r_{d_1 d}(t+1) \geq r_{min}(t)\gamma_d(t)$. Here, $\gamma_s(t)$ and $\gamma_d(t)$ are appropriately defined functions such that $\gamma_s(t)) > 1$ and $\gamma_d(t)) > 1$ for all $t \geq L$.

This would give us $r_{min}(t+L) \geq r_{min}(t)\gamma(t)$ where $\gamma(t) = min\{\gamma_s(t+L-1), \gamma_s(t+L-2), \cdots, \gamma_s(t), \gamma_d(t+L-1), \gamma_d(t+L-2), \cdots, \gamma_d(t)\}$. Below, we give the proof for $r_{ss_1}(t+1) \geq r_{min}(t)\gamma_s(t)$. The proof for $r_{d_1 d}(t+1) \geq r_{min}(t)\gamma_d(t)$ is similar.

Lemma 2. *For all time $t \geq L$, $r_{ss_1}(t+1) \geq r_{min}(t)\gamma_s(t)$, for some appropriately defined function $\gamma_s(t)$, such that $\gamma_s(t) > 1$ for all $t \geq L$.*

Proof. The pheromone levels on edges (s, s_1) and (s, s_2) at time $t+1$ is provided by the following expressions.

$$p_{ss_1}(t+1) = \delta\left(p_{ss_1}(t) + f_{\overrightarrow{ss_1}}(t) + f_{\overleftarrow{ss_1}}(t)\right), \quad p_{ss_2}(t+1) = \delta\left(p_{ss_2}(t) + f_{\overrightarrow{ss_2}}(t) + f_{\overleftarrow{ss_2}}(t)\right) \; .$$

Therefore, for $t \geq L$, we get

$$\frac{p_{ss_1}(t+1)}{p_{ss_2}(t+1)} = \frac{p_{ss_1}(t) + f_{\overrightarrow{ss_1}}(t) + f_{\overleftarrow{ss_1}}(t)}{p_{ss_2}(t) + f_{\overrightarrow{ss_2}}(t) + f_{\overleftarrow{ss_2}}(t)} \tag{15}$$

$$= \frac{p_{ss_1}(t) + f_{\overrightarrow{ss_1}}(t) + (1-l_{P_1})\, f_{\overleftarrow{d_1 d}}(t-m+1)}{p_{ss_2}(t) + f_{\overrightarrow{ss_2}}(t) + (1-l_{P_2})\, f_{\overleftarrow{d_2 d}}(t-n+1)} \tag{16}$$

$$= \frac{p_{ss_1}(t) + f_{\overrightarrow{ss_1}}(t) + \alpha\, f_{\overleftarrow{d_1 d}}(t-m+1)}{p_{ss_2}(t) + f_{\overrightarrow{ss_2}}(t) + \beta\, f_{\overleftarrow{d_2 d}}(t-n+1)} \; . \tag{17}$$

By the definition of $r_{ss_1}(t)$ and linear decision rule we know that,

$$\frac{p_{ss_1}(t)}{p_{ss_2}(t)} = \frac{f_{\overrightarrow{ss_1}}(t)}{f_{\overrightarrow{ss_2}}(t)} = r_{ss_1}(t) \geq r_{min}(t) \; .$$

From Lemma 1, we know that

$$\frac{f_{\overleftarrow{d_1 d}}(t-m+1)}{f_{\overleftarrow{d_2 d}}(t-n+1)} \geq r_{min}(t) \; .$$

For $t \geq L$, define

$$a(t) \stackrel{\text{def}}{=} \frac{r_{ss_1(t)}}{r_{min}(t)}, \; b(t) \stackrel{\text{def}}{=} \frac{f_{\overleftarrow{d_1 d}}(t-m+1)}{f_{\overleftarrow{d_2 d}}(t-n+1)} \frac{1}{r_{min}(t)} \; . \tag{18}$$

Note that, from the definition of $r_{min}(t)$ and using Lemma 1, we know that $a(t) \geq 1$ and $b(t) \geq 1$, for all $t \geq L$. Now, we can write

$$\frac{p_{ss_1}(t)}{p_{ss_2}(t)} = \frac{f_{\overrightarrow{ss_1}}(t)}{f_{\overrightarrow{ss_2}}(t)} = a(t) r_{min}(t), \; \frac{f_{\overleftarrow{d_1 d}}(t-m+1)}{f_{\overleftarrow{d_2 d}}(t-n+1)} = b(t) r_{min}(t) \tag{19}$$

Substituting this in Equation (17), we get

$$\begin{aligned} \frac{p_{ss_1}(t+1)}{p_{ss_2}(t+1)} &= r_{min}(t) \frac{a(t) p_{ss_2}(t) + a(t) f_{\overrightarrow{ss_2}}(t) + \alpha b(t) \; f_{\overleftarrow{d_2 d}}(t-n+1)}{p_{ss_2}(t) + f_{\overrightarrow{ss_2}}(t) + \beta \; f_{\overleftarrow{d_2 d}}(t-n+1)} & (20) \\ &= r_{min}(t) \gamma_s(t) & (21) \end{aligned}$$

where we define

$$\gamma_s(t) \stackrel{\text{def}}{=} \frac{a(t) p_{ss_2}(t) + a(t) f_{\overrightarrow{ss_2}}(t) + \alpha b(t) \; f_{\overleftarrow{d_2 d}}(t-n+1)}{p_{ss_2}(t) + f_{\overrightarrow{ss_2}}(t) + \beta \; f_{\overleftarrow{d_2 d}}(t-n+1)}. \tag{22}$$

Since $a(t) \geq 1$, $b(t) \geq 1$ and $\alpha > \beta$, we get that $\gamma_s(t) > 1$ for all $t \geq L$.

This completes the proof of the Lemma. □

Step 2. In this step, we will give a lower bound $\gamma_l > 1$, on $\gamma(t)$, to show that $r_{min}(t)$ increases sufficiently every L time steps. If the pheromone levels on the edges are too high as compared to the flow, it will take more time for the relative pheromone levels to change. We will first show that there exists a time T_1, such that for $t \geq T_1$, the pheromone levels and flow are comparable. Our lower bound $\gamma_l \leq \gamma(t)$ will hold for all $t \geq T_1$.

Lemma 3. *In the flow dynamics governed by the linear decision rule, the pheromone level on any edge $e = (u, v)$ is always bounded as follows:*

$$p_{uv}(t) \leq \frac{2(f_{\overrightarrow{s}} + f_{\overleftarrow{d}})}{1-\delta},$$

for all

$$t \geq T_1 \stackrel{\text{def}}{=} \max_{(u,v) \in E} \left(\frac{log\left(\frac{p_{uv}(0)}{f_{\overrightarrow{s}} + f_{\overleftarrow{d}}}\right)}{log\left(\frac{1}{\delta}\right)} \right) \; .$$

Proof. Consider any edge $e = (u, v)$ and time $t > 0$,

$$p_{uv}(t) = \delta(p_{uv}(t-1) + f_{\overrightarrow{uv}}(t-1) + f_{\overleftarrow{uv}}(t-1)) \leq \delta\left(p_{uv}(t-1) + f_{\overrightarrow{s}} + f_{\overleftarrow{d}}\right),$$
$$\leq (f_{\overrightarrow{s}} + f_{\overleftarrow{d}}) \sum_{i=1}^{t} \delta^i + \delta^t p_{uv}(0)$$

For $t \geq \frac{log\left(\frac{p_{uv}(0)}{f_{\overrightarrow{s}}+f_{\overleftarrow{d}}}\right)}{log\left(\frac{1}{\delta}\right)}$, we get

$$p_{uv}(t) \leq \frac{(f_{\overrightarrow{s}} + f_{\overleftarrow{d}})}{1-\delta} + (f_{\overrightarrow{s}} + f_{\overleftarrow{d}}) \leq \frac{2(f_{\overrightarrow{s}} + f_{\overleftarrow{d}})}{1-\delta}. \tag{23}$$

Since $T_1 \geq \frac{log\left(\frac{p_{uv}(0)}{f_{\overrightarrow{s}}+f_{\overleftarrow{d}}}\right)}{log\left(\frac{1}{\delta}\right)}$, we get that for $t \geq T_1$,

$$p_{uv}(t) \leq \frac{2(f_{\overrightarrow{s}} + f_{\overleftarrow{d}})}{1-\delta}. \tag{24}$$

□

In Equation (19), we defined $a(t)$ and $b(t)$ such that

$$\frac{p_{ss_1}(t)}{p_{ss_2}(t)} = \frac{f_{\overrightarrow{ss_1}}(t)}{f_{\overrightarrow{ss_2}}(t)} = a(t)r_{min}(t), \quad \frac{f_{\overleftarrow{d_1d}}(t-m+1)}{f_{\overleftarrow{d_2d}}(t-n+1)} = b(t)r_{min}(t)$$

Similarly, for $t \geq L$, we define $c(t) \stackrel{\text{def}}{=} \frac{r_{d_1d}(t-n+1)}{r_{min}(t)}$. From the definition of $r_{min}(t)$, we know that $c(t) \geq 1$ for all $t \geq L$. We will show a upper bound on $c(t)$ in terms of $b(t)$ which will be useful later.

Lemma 4. *For all $t \geq L$, $1 + c(t)r_{min}(t) \leq b(t)(1 + r_{min}(t))$.*

Proof. We know

$$r_{d_1d}(t-n+1) = \frac{\overline{p}_{\overleftarrow{d_1d}}(t-n+1)}{\overline{p}_{\overleftarrow{d_2d}}(t-n+1)} = c(t)r_{min}(t) \tag{25}$$

which gives

$$\frac{1 - \overline{p}_{\overleftarrow{d_2d}}(t-n+1)}{\overline{p}_{\overleftarrow{d_2d}}(t-n+1)} = c(t)r_{min}(t) \tag{26}$$

which gives $\frac{1}{\overline{p}_{\overleftarrow{d_2d}}(t-n+1)} = 1 + c(t)r_{min}(t)$. We also know

$$\frac{\overline{p}_{\overleftarrow{d_1d}}(t-m+1)}{\overline{p}_{\overleftarrow{d_2d}}(t-n+1)} = b(t)r_{min}(t). \tag{27}$$

Substituting $\frac{1}{\overline{p}_{\overleftarrow{d_2 d}}(t-n+1)} = 1 + c(t)r_{min}(t)$, we get

$$\overline{p}_{\overleftarrow{d_1 d}}(t-m+1) = \frac{b(t)r_{min}(t)}{1+c(t)r_{min}(t)} \ . \tag{28}$$

Finally, from the definition of $r_{min}(t)$, we know

$$\frac{\overline{p}_{\overleftarrow{d_1 d}}(t-m+1)}{\overline{p}_{\overleftarrow{d_2 d}}(t-m+1)} = r_{d_1 d}(t-m+1) \geq r_{min}(t). \tag{29}$$

Since $\overline{p}_{\overleftarrow{d_2 d}}(t-m+1) = 1 - \overline{p}_{\overleftarrow{d_1 d}}(t-m+1)$, this gives

$$\overline{p}_{\overleftarrow{d_1 d}}(t-m+1) \geq \frac{r_{min}(t)}{r_{min}(t)+1}. \tag{30}$$

Substituting the value $\overline{p}_{\overleftarrow{d_1 d}}(t-m+1) = \frac{b(t)r_{min}(t)}{1+c(t)r_{min}(t)}$, we get

$$\frac{b(t)r_{min}(t)}{1+c(t)r_{min}(t)} \geq \frac{r_{min}(t)}{r_{min}(t)+1}. \tag{31}$$

which implies

$$b(t)(1+r_{min}(t)) \geq 1 + c(t)r_{min}(t) \ . \tag{32}$$

This finishes the proof of the Lemma. □

Now, we come to the main part of step 2 where we show $\gamma(t) > \gamma_l$ for all $t \geq T_1 + L$, for some $\gamma_l > 1$. From step 1, we know that $\gamma(t) = min\{\gamma_s(t+L-1), \gamma_s(t+L-2), \cdots, \gamma_s(t), \gamma_d(t+L-1), \gamma_d(t+L-2), \cdots, \gamma_d(t)\}$. Therefore if we show that $\gamma_s(t) \geq \gamma_{s_l}$ and $\gamma_d(t) \geq \gamma_{d_l}$ for all $t \geq T_1 + L$, for some $\gamma_{s_l}, \gamma_{d_l} > 1$, we can set $\gamma_l = min(\gamma_{s_l}, \gamma_{d_l})$, and we will be done.

Below we will prove $\gamma_s(t) \geq \gamma_{s_l}$. The proof for $\gamma_d(t) \geq \gamma_{d_l}$ is similar.

Lemma 5. *For all time $t \geq L + T_1$, $r_{ss_1}(t+1) \geq r_{min}(t)\gamma_s(t)$ where $\gamma_s(t) \geq \gamma_{s_l}$ for some fixed constant $\gamma_{s_l} > 1$.*

Proof. From Lemma 2, we know that

$$\frac{p_{ss_1}(t+1)}{p_{ss_2}(t+1)} = r_{min}(t)\gamma_s(t) \tag{33}$$

where

$$\gamma_s(t) \stackrel{\text{def}}{=} \frac{a(t)p_{ss_2}(t) + a(t)f_{\overrightarrow{ss_2}}(t) + \alpha b(t)\, f_{\overleftarrow{d_2 d}}(t-n+1)}{p_{ss_2}(t) + f_{\overrightarrow{ss_2}}(t) + \beta\, f_{\overleftarrow{d_2 d}}(t-n+1)}.$$

We want to show that $\gamma_s(t) \geq \gamma_{s_l}$ for some fixed constant $\gamma_{s_l} > 1$. Using $a(t) \geq 1$ and $b(t) \geq 1$, we get

$$\gamma_s(t) \geq \frac{p_{ss_2}(t) + f_{\overrightarrow{ss_2}}(t) + b(t)\alpha\, f_{\overleftarrow{d_2 d}}(t-n+1)}{p_{ss_2}(t) + f_{\overrightarrow{ss_2}}(t) + b(t)\beta\, f_{\overleftarrow{d_2 d}}(t-n+1)} \tag{34}$$

$$= 1 + \frac{\alpha - \beta}{\frac{p_{ss_2}(t)}{b(t)f_{\overleftarrow{d_2 d}}(t-n+1)} + \frac{f_{\overrightarrow{ss_2}}(t)}{b(t)f_{\overleftarrow{d_2 d}}(t-n+1)} + \beta} \tag{35}$$

Now, to lower bound $\gamma_s(t)$, we need to upper bound $\frac{p_{ss_2}(t)}{b(t)f_{\overleftarrow{d_2d}}(t-n+1)}$ and $\frac{f_{\overrightarrow{ss_2}}(t)}{b(t)f_{\overleftarrow{d_2d}}(t-n+1)}$. Using the definition of normalized pheromone level, we can write

$$\frac{p_{ss_2}(t)}{b(t)f_{\overleftarrow{d_2d}}(t-n+1)} = \left(\frac{p_{ss_1}(t)+p_{ss_2}(t)}{f_{\overleftarrow{d}}}\right)\left(\frac{\overline{p}_{\overrightarrow{ss_2}}(t)}{b(t)\overline{p}_{\overleftarrow{d_2d}}(t-n+1)}\right) \tag{36}$$

$$\leq \left(\frac{4(f_{\overrightarrow{s}}+f_{\overleftarrow{d}})}{f_{\overleftarrow{d}}(1-\delta)}\right)\left(\frac{\overline{p}_{\overrightarrow{ss_2}}(t)}{b(t)\overline{p}_{\overleftarrow{d_2d}}(t-n+1)}\right) \tag{37}$$

where we used the upper bound for pheromone level shown in Lemma 3. Similarly, we can write

$$\frac{f_{\overrightarrow{ss_2}}(t)}{b(t)f_{\overleftarrow{d_2d}}(t-n+1)} = \left(\frac{f_{\overrightarrow{s}}}{f_{\overleftarrow{d}}}\right)\left(\frac{\overline{p}_{\overrightarrow{ss_2}}(t)}{b(t)\overline{p}_{\overleftarrow{d_2d}}(t-n+1)}\right). \tag{38}$$

Combining Equation (37) and Equation (38), we get

$$\frac{p_{ss_2}(t)}{b(t)f_{\overleftarrow{d_2d}}(t-n+1)} + \frac{f_{\overrightarrow{ss_2}}(t)}{b(t)f_{\overleftarrow{d_2d}}(t-n+1)} \leq \left(\frac{4(f_{\overrightarrow{s}}+f_{\overleftarrow{d}})}{f_{\overleftarrow{d}}(1-\delta)} + \frac{f_{\overrightarrow{s}}}{f_{\overleftarrow{d}}}\right)\left(\frac{\overline{p}_{\overrightarrow{ss_2}}(t)}{b(t)\overline{p}_{\overleftarrow{d_2d}}(t-n+1)}\right) \tag{39}$$

$$\leq \left(\frac{5(f_{\overrightarrow{s}}+f_{\overleftarrow{d}})}{f_{\overleftarrow{d}}(1-\delta)}\right)\left(\frac{\overline{p}_{\overrightarrow{ss_2}}(t)}{b(t)\overline{p}_{\overleftarrow{d_2d}}(t-n+1)}\right) \tag{40}$$

$$= C_{f_{\overrightarrow{s}},f_{\overleftarrow{d}},\delta}\left(\frac{\overline{p}_{\overrightarrow{ss_2}}(t)}{b(t)\overline{p}_{\overleftarrow{d_2d}}(t-n+1)}\right) \tag{41}$$

where we define $C_{f_{\overrightarrow{s}},f_{\overleftarrow{d}},\delta} \stackrel{\text{def}}{=} \left(\frac{5(f_{\overrightarrow{s}}+f_{\overleftarrow{d}})}{f_{\overleftarrow{d}}(1-\delta)}\right)$. Now, we upper bound $\frac{\overline{p}_{\overrightarrow{ss_2}}(t)}{b(t)\overline{p}_{\overleftarrow{d_2d}}(t-n+1)}$. From the definition of normalized pheromone level, we get

$$\frac{\overline{p}_{\overrightarrow{ss_2}}(t)}{b(t)\overline{p}_{\overleftarrow{d_2d}}(t-n+1)} = \frac{1}{b(t)}\left(\frac{1+r_{d_1d}(t-n+1)}{1+r_{ss_1}(t)}\right)$$

We know $r_{d_1d}(t-n+1) = c(t)r_{min}(t)$ where $c(t) \geq 1$, and we earlier defined $r_{ss_1}(t) = a(t)r_{min}$ where $a(t) \geq 1$. This gives us

$$\frac{\overline{p}_{\overrightarrow{ss_2}}(t)}{b(t)\overline{p}_{\overleftarrow{d_2d}}(t-n+1)} = \frac{1}{b(t)}\left(\frac{1+c(t)r_{min}(t)}{1+a(t)r_{min}(t)}\right)$$

$$\leq \frac{1}{b(t)}\left(\frac{1+c(t)r_{min}(t)}{1+r_{min}(t)}\right)$$

In Lemma 4, we show that $(1+c(t)r_{min}(t)) \leq b(t)(1+r_{min}(t))$, which gives us

$$\frac{\overline{p}_{\overrightarrow{ss_2}}(t)}{b(t)\overline{p}_{\overleftarrow{d_2d}}(t-n+1)} \leq 1$$

Substituting this in Equation (41), we get

$$\frac{p_{ss_2}(t)}{b(t)f_{\overleftarrow{d_2d}}(t-n+1)} + \frac{f_{\overrightarrow{ss_2}}(t)}{b(t)f_{\overleftarrow{d_2d}}(t-n+1)} \leq C_{f_{\overrightarrow{s}},f_{\overleftarrow{d}},\delta} \tag{42}$$

Substituting this in Equation (35), we get

$$\gamma_s(t) \geq 1 + \frac{\alpha-\beta}{C_{f_{\overrightarrow{s}},f_{\overleftarrow{d}},\delta}+\beta} \tag{43}$$

Define $\gamma_{s_l} \stackrel{\text{def}}{=} 1 + \frac{\alpha-\beta}{C_{f_{\overrightarrow{s}},f_{\overleftarrow{d}},\delta}+\beta}$. Since $\gamma_{s_l} > 1$, this completes the proof of the lemma. □

Using a very similar proof, we can bound $\gamma_d(t)$ by γ_{d_l}. Finally, setting $\gamma_l = min(\gamma_{s_l}, \gamma_{d_l})$, we will get a lower bound $\gamma_l > 1$ on $\gamma(t)$, for all $t \geq T + L$. This finishes the proof for step 2.

Step 3. In step 1, we show that $r_{min}(t)$ is non-decreasing for $t \geq L$, therefore $r_{min}(L+T_1) \geq r_{min}(L)$. And from step 2, we know that $r_{min}(t)$ increases at least by a factor of γ_l every L time steps, for all $t \geq L + T_1$. This gives us $r_{min}(t) \geq r_{min}(L)\gamma_l^{\lfloor\frac{t-L-T_1}{L}\rfloor}$. Let $T_2 = L\frac{log\left(\frac{2}{\epsilon r_{min}(L)}\right)}{log(\gamma_l)}$. For $t \geq L + T_1 + T_2$ time steps, we would get that $r_{min} \geq \frac{2}{\epsilon}$, which implies $\overline{p}_{\overrightarrow{ss_1}}(t) \geq 1-\epsilon$ and $\overline{p}_{\overleftarrow{d_1d}}(t) \geq 1-\epsilon$. Since the pheromone level on all edges on P_1 is always non-zero, we trivially know that $\overline{p}_{\overrightarrow{uv}}(t) = 1$ for $(u,v) \in P_1 \setminus (s, s_1)$ and $\overline{p}_{\overleftarrow{uv}}(t) = 1$ for $(u,v) \in P_1 \setminus (d_1, d)$. This gives us that for $t \geq L + T_1 + T_2$, normalized pheromone level on all edges on P_1 are at least $1-\epsilon$, where

$$T_1 = \max_{(u,v)\in E}\left(\frac{log\left(\frac{p_{uv}(0)}{f_{\overrightarrow{s}}+f_{\overleftarrow{d}}}\right)}{log\left(\frac{1}{\delta}\right)}\right)$$

$$T_2 = L\frac{log\left(\frac{2}{\epsilon r_{min}(L)}\right)}{log(\gamma_l)}$$

This completes the proof of Theorem 1.

A.2 Increasing flow with no leakage on both paths (Theorem 2)

Here, we provide a proof of Theorem 2. We restate it below.

Theorem 2. *Consider a graph G consisting of two parallel paths P_1 and P_2 from s to d. Let the flow and the pheromone levels be updated according to the model in Section 2, and let P_1 be the shorter path. If (i) the initial pheromone level $p_{uv}(0)$ is positive for all edges $(u,v) \in P_1$, (ii) leakage $l_{P_1} = l_{P_2} = 0$, and (iii) the incoming flow increases as follows:*

1. ***Multiplicative increase:*** *$f_{\overrightarrow{s}}(t) = \alpha^t f_{\overrightarrow{s}}(0)$ and $f_{\overrightarrow{d}}(t) = \alpha^t f_{\overrightarrow{d}}(0)$, for any $\alpha > 1$, or*
2. ***Additive increase:*** *$f_{\overrightarrow{s}}(t) = f_{\overrightarrow{s}}(0) + \alpha t$ and $f_{\overrightarrow{d}}(t) = f_{\overrightarrow{d}}(0) + \alpha t$, for any $\alpha > 0$,*

then the flow dynamics governed by the linear decision rule converges to a state where all the flow goes through P_1.

Let s_1, s_2 be the neighboring vertices of s that belong to paths P_1 and P_2 respectively. Similarly let d_1 and d_2 be the corresponding neighbors for d. Define $r_{ss_1}(t) \stackrel{\text{def}}{=} \frac{p_{ss_1}(t)}{p_{ss_2}(t)}$ and $r_{d_1d}(t) \stackrel{\text{def}}{=} \frac{p_{d_1d}(t)}{p_{d_2d}(t)}$ to be the relative pheromone levels at (s, s_1) and (d_1, d) respectively. For notational simplicity, we define $m \stackrel{\text{def}}{=} len_{P_1}$ and $n \stackrel{\text{def}}{=} len_{P_2}$ to be the lengths of path P_1 and P_2, and $L \stackrel{\text{def}}{=} max(m, n)$.

Our potential function at any time $t \geq L$ is given by the minimum of the relative pheromone levels $r_{ss_1}(t)$ and $r_{d_1d}(t)$ across the last L time steps:

$$r_{min}(t) \stackrel{\text{def}}{=} min\{r_{ss_1}(t), r_{ss_1}(t-1), \cdots, r_{ss_1}(t-L+1), r_{d_1d}(t), r_{d_1d}(t-1), \cdots, r_{d_1d}(t-L+1)\} \tag{44}$$

The proof is similar to the proof for the case of leakage with constant flow. We divide our proof into 3 steps:

- **Step 1**: In this step, we show that $r_{min}(t)$ is non-decreasing at every time step and increases by a factor of $\gamma(t)$ every L time steps, for all $t \geq L$. Here, $\gamma(t)$ is some appropriately defined function which is greater than 1 for all $t \geq L$.
- **Step 2**: In this step, we will lower bound $\gamma(t)$ to show that $r_{min}(t)$ increases sufficiently every L time steps.
- **Step 3**: We will find the rate of convergence based on the rate of increase shown in step 2.

Now, we give proofs for each of these three steps.

Step 1. We will use the following lemma for the proof.

Lemma 6. *For any time $t \geq L$, the following is true,*

$$\frac{\overline{p}_{\overleftarrow{d_1d}}(t-m+1)}{\overline{p}_{\overleftarrow{d_2d}}(t-n+1)} \geq \min(r_{d_1d}(t-m+1), r_{d_1d}(t-n+1)) \geq r_{min}(t) \ .$$

Proof. Under the linear decision rule we know that,

$$\overline{p}_{\overleftarrow{d_1d}}(t-m+1) = \frac{r_{d_1d}(t-m+1)}{r_{d_1d}(t-m+1)+1} \tag{45}$$

$$\overline{p}_{\overleftarrow{d_2d}}(t-n+1) = \frac{1}{r_{d_1d}(t-n+1)+1} \tag{46}$$

$$\tag{47}$$

Let $r \stackrel{\text{def}}{=} \min(r_{d_1d}(t-m+1), r_{d_1d}(t-n+1))$, then note that $\frac{r_{d_1d}(t-m+1)}{r_{dd_1}(t-m+1)+1} \geq \frac{r}{r+1}$ and $\frac{1}{r_{d_1d}(t-n+1)+1} \leq \frac{1}{r+1}$. Therefore,

$$\frac{\overline{p}_{\overleftarrow{d_1d}}(t-m+1)}{\overline{p}_{\overleftarrow{d_2d}}(t-n+1)} \geq r = \min(r_{d_1d}(t-m+1), r_{d_1d}(t-n+1)) \ .$$

and from the definition of $r_{min}(t)$, we know that $\min(r_{d_1d}(t-m+1), r_{d_1d}(t-n+1)) \geq r_{min}(t)$. □

Now we show that $r_{min}(t)$ is non-decreasing at every time step and increases by a factor of $\gamma(t)$ every L time steps, for all $t \geq L$. To show this, we would show that $r_{ss_1}(t+1) \geq r_{min}(t)\gamma_s(t)$, and $r_{d_1d}(t+1) \geq r_{min}(t)\gamma_d(t)$. Here, $\gamma_s(t)$ and $\gamma_d(t)$ are appropriately defined functions such that $\gamma_s(t)) > 1$ and $\gamma_d(t)) > 1$ for all $t \geq L$.

This would give us $r_{min}(t+L) \geq r_{min}(t)\gamma(t)$ where $\gamma(t) = min\{\gamma_s(t+L-1), \gamma_s(t+L-2), \cdots, \gamma_s(t), \gamma_d(t+L-1), \gamma_d(t+L-2), \cdots, \gamma_d(t)\}$. Below, we give the proof for $r_{ss_1}(t+1) \geq r_{min}(t)\gamma_s(t)$. The proof for $r_{d_1d}(t+1) \geq r_{min}(t)\gamma_d(t)$ is similar.

Lemma 7. *For all time $t \geq L$, $r_{ss_1}(t+1) \geq r_{min}(t)\gamma_s(t)$, for some appropriately defined function $\gamma_s(t)$, such that $\gamma_s(t) > 1$ for all $t \geq L$.*

Proof. The pheromone levels on edges (s, s_1) and (s, s_2) at time $t+1$ is provided by the following expressions.

$$p_{ss_1}(t+1) = \delta\left(p_{ss_1}(t) + f_{\overrightarrow{ss_1}}(t) + f_{\overleftarrow{ss_1}}(t)\right) \quad p_{ss_2}(t+1) = \delta\left(p_{ss_2}(t) + f_{\overrightarrow{ss_2}}(t) + f_{\overleftarrow{ss_2}}(t)\right) .$$

Therefore, for $t \geq L$, we get

$$\frac{p_{ss_1}(t+1)}{p_{ss_2}(t+1)} = \frac{p_{ss_1}(t) + f_{\overrightarrow{ss_1}}(t) + f_{\overleftarrow{ss_1}}(t)}{p_{ss_2}(t) + f_{\overrightarrow{ss_2}}(t) + f_{\overleftarrow{ss_2}}(t)} \tag{48}$$

$$= \frac{p_{ss_1}(t) + f_{\overrightarrow{ss_1}}(t) + (1 - l_{P_1})\, f_{\overleftarrow{d_1d}}(t-m+1)}{p_{ss_2}(t) + f_{\overrightarrow{ss_2}}(t) + (1 - l_{P_2})\, f_{\overleftarrow{d_2d}}(t-n+1)} \tag{49}$$

$$= \frac{p_{ss_1}(t) + f_{\overrightarrow{ss_1}}(t) + f_{\overleftarrow{d_1d}}(t-m+1)}{p_{ss_2}(t) + f_{\overrightarrow{ss_2}}(t) + f_{\overleftarrow{d_2d}}(t-n+1)} \tag{50}$$

$$= \frac{p_{ss_1}(t) + f_{\overrightarrow{ss_1}}(t) + f_{\overleftarrow{d}}(t-m+1)\overline{p}_{\overleftarrow{d_1d}}(t-m+1)}{p_{ss_2}(t) + f_{\overrightarrow{ss_2}}(t) + f_{\overleftarrow{d}}(t-n+1)\overline{p}_{\overleftarrow{d_2d}}(t-n+1)} . \tag{51}$$

where we used $l_{P_1} = l_{P_2} = 0$ in Equation (50) and express the flow in terms of normalized pheromone level in Equation (51). By the definition of $r_{ss_1}(t)$ and linear decision rule we know that,

$$\frac{p_{ss_1}(t)}{p_{ss_2}(t)} = \frac{f_{\overrightarrow{ss_1}}(t)}{f_{\overrightarrow{ss_2}}(t)} = r_{ss_1}(t) \geq r_{min}(t) .$$

From Lemma 6, we know that

$$\frac{\overline{p}_{\overleftarrow{d_1d}}(t-m+1)}{\overline{p}_{\overleftarrow{d_2d}}(t-n+1)} \geq r_{min}(t) .$$

For $t \geq L$, define

$$a(t) \stackrel{\text{def}}{=} \frac{r_{ss_1(t)}}{r_{min}(t)},\ b(t) \stackrel{\text{def}}{=} \frac{\overline{p}_{\overleftarrow{d_1d}}(t-m+1)}{\overline{p}_{\overleftarrow{d_2d}}(t-n+1)} \frac{1}{r_{min}(t)} . \tag{52}$$

Note that, from the definition of $r_{min}(t)$ and using Lemma 6, we know that $a(t) \geq 1$ and $b(t) \geq 1$, for all $t \geq L$. Now, we can write

$$\frac{p_{ss_1}(t)}{p_{ss_2}(t)} = \frac{f_{\overrightarrow{ss_1}}(t)}{f_{\overrightarrow{ss_2}}(t)} = a(t)r_{min}(t),\ \frac{\overline{p}_{\overleftarrow{d_1d}}(t-m+1)}{\overline{p}_{\overleftarrow{d_2d}}(t-n+1)} = b(t)r_{min}(t) \tag{53}$$

Substituting this in Equation (51), we get

$$\frac{p_{ss_1}(t+1)}{p_{ss_2}(t+1)} = r_{min}(t)\frac{a(t)p_{ss_2}(t) + a(t)f_{\overrightarrow{ss_2}}(t) + b(t)\ \ f_{\overleftarrow{d}}(t-m+1)\overline{p}_{\overleftarrow{d_2 d}}(t-n+1)}{p_{ss_2}(t) + f_{\overrightarrow{ss_2}}(t) +\ f_{\overleftarrow{d}}(t-n+1)\overline{p}_{\overleftarrow{d_2 d}}(t-n+1)} \tag{54}$$

$$= r_{min}(t)\gamma_s(t) \tag{55}$$

where we define

$$\gamma_s(t) \stackrel{\text{def}}{=} \frac{a(t)p_{ss_2}(t) + a(t)f_{\overrightarrow{ss_2}}(t) + b(t)\ \ f_{\overleftarrow{d}}(t-m+1)\overline{p}_{\overleftarrow{d_2 d}}(t-n+1)}{p_{ss_2}(t) + f_{\overrightarrow{ss_2}}(t) +\ f_{\overleftarrow{d}}(t-n+1)\overline{p}_{\overleftarrow{d_2 d}}(t-n+1)}. \tag{56}$$

Since the flow is flow is increasing with time, $f_{\overleftarrow{d}}(t-m+1) > f_{\overleftarrow{d}}(t-n+1)$, as $n > m$. Also, $a(t) \geq 1$, $b(t) \geq 1$. Therefore, we get that $\gamma_s(t) > 1$ for all $t \geq L$.

This completes the proof of the Lemma. □

Step 2. In this step, we will lower bound $\gamma(t)$ to show that $r_{min}(t)$ increases sufficiently every L time steps. For the multiplicative increase case, we will show that $\gamma(t) \geq \left(1 + \frac{c_1}{\alpha^{len_{P_2}}}\right)$ for some constant $c_1 > 0$. For the additive increase case, we will show that $\gamma(t) \geq \left(1 + \frac{c_2}{t}\right)$ for some constant $c_2 > 0$. If the pheromone levels on the edges are too high as compared to the flow, it will take more time for the relative pheromone levels to change. So we will first show that there exists a time T_1, such that for $t \geq T_1$, the pheromone levels and flow are comparable. Our lower bounds for $\gamma(t)$ will hold for all $t \geq T_1$.

Lemma 8. *In the flow dynamics governed by the linear decision rule, the pheromone level on any edge $e = (u, v)$ is always bounded as follows:*

$$p_{uv}(t) \leq \frac{2(f_{\overrightarrow{s}}(t) + f_{\overleftarrow{d}}(t))}{1-\delta}\ .$$

for all

$$t \geq T_1 \stackrel{\text{def}}{=} \max_{(u,v)\in E}\left(\frac{log\left(\frac{p_{uv}(0)}{f_{\overrightarrow{s}}(0)+f_{\overleftarrow{d}}(0)}\right)}{log\left(\frac{1}{\delta}\right)}\right)$$

Proof. Consider any edge $e = (u, v)$ and time $t > 0$,

$$\begin{aligned} p_{uv}(t) &= \delta(p_{uv}(t-1) + f_{\overrightarrow{uv}}(t-1) + f_{\overleftarrow{uv}}(t-1)) \leq \delta\left(p_{uv}(t-1) + f_{\overrightarrow{s}}(t-1) + f_{\overleftarrow{d}}(t-1)\right)\ , \\ &\leq \sum_{i=1}^{t}\delta^i(f_{\overrightarrow{s}}(i-1) + f_{\overleftarrow{d}}(i-1)) + \delta^t p_{uv}(0) \\ &\leq (f_{\overrightarrow{s}}(t) + f_{\overleftarrow{d}}(t))\sum_{i=1}^{t}\delta^i + \delta^t p_{uv}(0) \end{aligned}$$

where we used that $f_{\overrightarrow{s}}(t)$ and $f_{\overleftarrow{d}}(t)$ are monotonically increasing in the last inequality. For $t \geq \frac{log\left(\frac{p_{uv}(0)}{f_{\overrightarrow{s}}(0)+f_{\overleftarrow{d}}(0)}\right)}{log\left(\frac{1}{\delta}\right)}$, we get

$$p_{uv}(t) \leq \frac{(f_{\overrightarrow{s}}(t) + f_{\overleftarrow{d}}(t))}{1-\delta} + (f_{\overrightarrow{s}}(0) + f_{\overleftarrow{d}}(0)) \leq \frac{2(f_{\overrightarrow{s}}(t) + f_{\overleftarrow{d}}(t))}{1-\delta}. \tag{57}$$

where we used that $f_{\overrightarrow{s}}(t)$ and $f_{\overleftarrow{d}}(t)$ are monotonically increasing. Since $T_1 \geq \frac{log\left(\frac{p_{uv}(0)}{f_{\overrightarrow{s}}(0)+f_{\overleftarrow{d}}(0)}\right)}{log\left(\frac{1}{\delta}\right)}$, we get that for $t \geq T_1$,

$$p_{uv}(t) \leq \frac{2(f_{\overrightarrow{s}}(t) + f_{\overleftarrow{d}}(t))}{1-\delta}. \tag{58}$$

□

In Equation (53), we defined $a(t)$ and $b(t)$ such that

$$\frac{p_{ss_1}(t)}{p_{ss_2}(t)} = \frac{f_{\overrightarrow{ss_1}}(t)}{f_{\overrightarrow{ss_2}}(t)} = a(t)r_{min}(t), \ \frac{\overline{p}_{\overleftarrow{d_1d}}(t-m+1)}{\overline{p}_{\overleftarrow{d_2d}}(t-n+1)} = b(t)r_{min}(t)$$

Similarly, for $t \geq L$, we define $c(t) \overset{\text{def}}{=} \frac{r_{d_1d}(t-n+1)}{r_{min}(t)}$. From the definition of $r_{min}(t)$, we know that $c(t) \geq 1$ for all $t \geq L$. We will show a relationship between $b(t)$ and $c(t)$ which will be useful later.

Lemma 9. *For all $t \geq L$, $1 + c(t)r_{min}(t) \leq b(t)(1 + r_{min}(t))$.*

Proof. We know

$$r_{d_1d}(t-n+1) = \frac{\overline{p}_{\overleftarrow{d_1d}}(t-n+1)}{\overline{p}_{\overleftarrow{d_2d}}(t-n+1)} = c(t)r_{min}(t) \tag{59}$$

which gives

$$\frac{1-\overline{p}_{\overleftarrow{d_2d}}(t-n+1)}{\overline{p}_{\overleftarrow{d_2d}}(t-n+1)} = c(t)r_{min}(t) \tag{60}$$

which gives $\frac{1}{\overline{p}_{\overleftarrow{d_2d}}(t-n+1)} = 1 + c(t)r_{min}(t)$. We also know

$$\frac{\overline{p}_{\overleftarrow{d_1d}}(t-m+1)}{\overline{p}_{\overleftarrow{d_2d}}(t-n+1)} = b(t)r_{min}(t)\ . \tag{61}$$

Substituting $\frac{1}{\overline{p}_{\overleftarrow{d_2d}}(t-n+1)} = 1 + c(t)r_{min}(t)$, we get

$$\overline{p}_{\overleftarrow{d_1d}}(t-m+1) = \frac{b(t)r_{min}(t)}{1+c(t)r_{min}(t)}\ . \tag{62}$$

Finally, from the definition of $r_{min}(t)$, we know

$$\frac{\overline{p}_{\overleftarrow{d_1d}}(t-m+1)}{\overline{p}_{\overleftarrow{d_2d}}(t-m+1)} = r_{d_1d}(t-m+1) \geq r_{min}(t). \tag{63}$$

Since $\overline{p}_{\overleftarrow{d_2d}}(t-m+1) = 1 - \overline{p}_{\overleftarrow{d_1d}}(t-m+1)$, this gives

$$\overline{p}_{\overleftarrow{d_1d}}(t-m+1) \geq \frac{r_{min}(t)}{r_{min}(t)+1}. \tag{64}$$

Substituting the value $\overline{p}_{\overleftarrow{d_1 d}}(t-m+1) = \frac{b(t) r_{min}(t)}{1+c(t) r_{min}(t)}$, we get

$$\frac{b(t) r_{min}(t)}{1+c(t) r_{min}(t)} \geq \frac{r_{min}(t)}{r_{min}(t)+1}. \tag{65}$$

which implies

$$b(t)(1+r_{min}(t)) \geq 1 + c(t) r_{min}(t)\ . \tag{66}$$

This finishes the proof of the Lemma.

□

Now, we come to the main part of step 2 where we lower bound $\gamma(t)$ for all $t \geq T_1 + L$. From step 1, we know that $\gamma(t) = min\{\gamma_s(t+L-1), \gamma_s(t+L-2), \cdots, \gamma_s(t), \gamma_d(t+L-1), \gamma_d(t+L-2), \cdots, \gamma_d(t)\}$. Therefore, to lower bound $\gamma(t)$, we need to lower bound $\gamma_s(t)$ and $\gamma_d(t)$.

Below we will prove a lower bound for $\gamma_s(t)$.

Lemma 10. *For all time $t \geq L + T_1$, $r_{ss_1}(t+1) \geq r_{min}(t)\gamma_s(t)$ where*

$$\gamma_s(t) \geq\ 1 + \frac{(1-\delta)\left(f_{\overleftarrow{d}}(t-m+1) - f_{\overleftarrow{d}}(t-n+1)\right)}{6(f_{\overrightarrow{s}}(t) + f_{\overleftarrow{d}}(t))}$$

Proof. From Lemma 7, we know that

$$\frac{p_{ss_1}(t+1)}{p_{ss_2}(t+1)} = r_{min}(t)\gamma_s(t) \tag{67}$$

where

$$\gamma_s(t) \stackrel{\text{def}}{=} \frac{a(t) p_{ss_2}(t) + a(t) f_{\overrightarrow{ss_2}}(t) + b(t)\ \ f_{\overleftarrow{d}}(t-m+1)\overline{p}_{\overleftarrow{d_2 d}}(t-n+1)}{p_{ss_2}(t) + f_{\overrightarrow{ss_2}}(t) +\ f_{\overleftarrow{d}}(t-n+1)\overline{p}_{\overleftarrow{d_2 d}}(t-n+1)}$$

Using $a(t) \geq 1$ and $b(t) \geq 1$, we get

$$\begin{aligned} \gamma_s(t) &\geq \frac{p_{ss_2}(t) + f_{\overrightarrow{ss_2}}(t) + b(t)\ \ f_{\overleftarrow{d}}(t-m+1)\overline{p}_{\overleftarrow{d_2 d}}(t-n+1)}{a(t) p_{ss_2}(t) + a(t) f_{\overrightarrow{ss_2}}(t) +\ b(t) f_{\overleftarrow{d}}(t-n+1)\overline{p}_{\overleftarrow{d_2 d}}(t-n+1)} & (68) \\ &= 1 + \frac{f_{\overleftarrow{d}}(t-m+1) - f_{\overleftarrow{d}}(t-n+1)}{\frac{p_{ss_2}(t)}{b(t)\overline{p}_{\overleftarrow{d_2 d}}(t-n+1)} + \frac{f_{\overrightarrow{ss_2}}(t)}{b(t)\overline{p}_{\overleftarrow{d_2 d}}(t-n+1)} + f_{\overleftarrow{d}}(t-n+1)} & (69) \end{aligned}$$

Now, to lower bound $\gamma_s(t)$, we need to upper bound $\frac{p_{ss_2}(t)}{b(t)\overline{p}_{\overleftarrow{d_2 d}}(t-n+1)}$ and $\frac{f_{\overrightarrow{ss_2}}(t)}{b(t)\overline{p}_{\overleftarrow{d_2 d}}(t-n+1)}$. Using the definition of normalized pheromone level, we can write

$$\begin{aligned} \frac{p_{ss_2}(t)}{b(t)\overline{p}_{\overleftarrow{d_2 d}}(t-n+1)} &= (p_{ss_1}(t) + p_{ss_2}(t))\left(\frac{\overline{p}_{\overrightarrow{ss_2}}(t)}{b(t)\overline{p}_{\overleftarrow{d_2 d}}(t-n+1)}\right) & (70) \\ &\leq \left(\frac{4(f_{\overrightarrow{s}}(t) + f_{\overleftarrow{d}}(t))}{1-\delta}\right)\left(\frac{\overline{p}_{\overrightarrow{ss_2}}(t)}{b(t)\overline{p}_{\overleftarrow{d_2 d}}(t-n+1)}\right) & (71) \end{aligned}$$

where we used the upper bound for pheromone level shown in Lemma 8. Similarly, we can write

$$\frac{f_{\overrightarrow{ss_2}}(t)}{b(t)\overline{p}_{\overleftarrow{d_2d}}(t-n+1)} = f_{\overrightarrow{s}}(t)\left(\frac{\overline{p}_{\overrightarrow{ss_2}}(t)}{b(t)\overline{p}_{\overleftarrow{d_2d}}(t-n+1)}\right). \tag{72}$$

Combining Equation (71) and Equation (72), we get

$$\frac{p_{ss_2}(t)}{b(t)\overline{p}_{\overleftarrow{d_2d}}(t-n+1)} + \frac{f_{\overrightarrow{ss_2}}(t)}{b(t)\overline{p}_{\overleftarrow{d_2d}}(t-n+1)} \leq \left(\frac{4(f_{\overrightarrow{s}}(t)+f_{\overleftarrow{d}}(t))}{(1-\delta)} + f_{\overrightarrow{s}}(t)\right)\left(\frac{\overline{p}_{\overrightarrow{ss_2}}(t)}{b(t)\overline{p}_{\overleftarrow{d_2d}}(t-n+1)}\right) \tag{73}$$

$$\leq \left(\frac{5(f_{\overrightarrow{s}}(t)+f_{\overleftarrow{d}}(t))}{(1-\delta)}\right)\left(\frac{\overline{p}_{\overrightarrow{ss_2}}(t)}{b(t)\overline{p}_{\overleftarrow{d_2d}}(t-n+1)}\right) \tag{74}$$

Now, we upper bound $\left(\frac{\overline{p}_{\overrightarrow{ss_2}}(t)}{b(t)\overline{p}_{\overleftarrow{d_2d}}(t-n+1)}\right)$. From the definition of normalized pheromone level, we get

$$\frac{\overline{p}_{\overrightarrow{ss_2}}(t)}{b(t)\overline{p}_{\overleftarrow{d_2d}}(t-n+1)} = \frac{1}{b(t)}\left(\frac{1+r_{d_1d}(t-n+1)}{1+r_{ss_1}(t)}\right)$$

We know $r_{d_1d}(t-n+1) = c(t)r_{min}(t)$ where $c(t) \geq 1$, and we earlier defined $r_{ss_1}(t) = a(t)r_{min}$ where $a(t) \geq 1$. This gives us

$$\frac{\overline{p}_{\overrightarrow{ss_2}}(t)}{b(t)\overline{p}_{\overleftarrow{d_2d}}(t-n+1)} = \frac{1}{b(t)}\left(\frac{1+c(t)r_{min}(t)}{1+a(t)r_{min}(t)}\right)$$

$$\leq \frac{1}{b(t)}\left(\frac{1+c(t)r_{min}(t)}{1+r_{min}(t)}\right)$$

In Lemma 9, we show that $(1+c(t)r_{min}(t)) \leq b(t)(1+r_{min}(t))$, which gives

$$\frac{\overline{p}_{\overrightarrow{ss_2}}(t)}{b(t)\overline{p}_{\overleftarrow{d_2d}}(t-n+1)} \leq 1$$

Substituting this in Equation (74), we get

$$\frac{p_{ss_2}(t)}{bf_{\overleftarrow{d_2d}}(t-n+1)} + \frac{f_{\overrightarrow{ss_2}}(t)}{bf_{\overleftarrow{d_2d}}(t-n+1)} \leq \frac{5(f_{\overrightarrow{s}}(t)+f_{\overleftarrow{d}}(t))}{(1-\delta)} \tag{75}$$

Substituting this in Equation (69), we get

$$\gamma_s(t) \geq 1 + \frac{f_{\overleftarrow{d}}(t-m+1) - f_{\overleftarrow{d}}(t-n+1)}{\left(\frac{5(f_{\overrightarrow{s}}(t)+f_{\overleftarrow{d}}(t))}{(1-\delta)}\right) + f_{\overleftarrow{d}}(t-n+1)} \tag{76}$$

Since the flow is monotonically increasing, we can further simplify this to get

$$\gamma_s(t) \geq\ 1 + \frac{f_{\overleftarrow{d}}(t-m+1) - f_{\overleftarrow{d}}(t-n+1)}{\left(\frac{6(f_{\overrightarrow{s}}(t)+f_{\overleftarrow{d}}(t))}{(1-\delta)}\right)} \tag{77}$$

$$=\ 1 + \frac{(1-\delta)\left(f_{\overleftarrow{d}}(t-m+1) - f_{\overleftarrow{d}}(t-n+1)\right)}{6(f_{\overrightarrow{s}}(t)+f_{\overleftarrow{d}}(t))} \tag{78}$$

□

Using a similar argument, we can bound $\gamma_d(t)$ as

$$\gamma_d(t) \geq \; 1 + \frac{(1-\delta)\left(f_{\overrightarrow{s}}(t-m+1) - f_{\overrightarrow{s}}(t-n+1)\right)}{6(f_{\overrightarrow{s}}(t) + f_{\overleftarrow{d}}(t))}.$$

Now, using these lower bounds on $\gamma_s(t)$ and $\gamma_l(t)$, we can lower bound $\gamma(t)$ for the multiplicative increase and additive increase case.

Lemma 11. *Consider the multiplicative increase case, that is, when $f_{\overrightarrow{s}}(t) = \alpha^t f_{\overrightarrow{s}}(0)$ and $f_{\overleftarrow{d}}(t) = \alpha^t f_{\overrightarrow{d}}(0)$ for some $\alpha > 1$. For all $t \geq L + T_1$.*

$$\gamma(t) \geq C_{f_{\overrightarrow{s}}(0), f_{\overleftarrow{d}}(0), \delta, \alpha, m} > 1$$

where $C_{f_{\overrightarrow{s}}(0), f_{\overleftarrow{d}}(0), \delta, \alpha, m}$ is some constant dependent on $f_{\overrightarrow{s}}(0), f_{\overleftarrow{d}}(0), \delta, \alpha$ and m.

Proof. From Lemma 10, we know that

$$\begin{aligned}
\gamma_s(t) \geq \; & 1 + \frac{(1-\delta)\left(f_{\overleftarrow{d}}(t-m+1) - f_{\overleftarrow{d}}(t-n+1)\right)}{6(f_{\overrightarrow{s}}(t) + f_{\overleftarrow{d}}(t))} \\
&= 1 + \frac{(1-\delta)\left(f_{\overleftarrow{d}}(0)\alpha^{t-m+1} - f_{\overleftarrow{d}}(0)\alpha^{t-n+1}\right)}{6(f_{\overrightarrow{s}}(0) + f_{\overleftarrow{d}}(0))\alpha^t} \\
&= 1 + \frac{(1-\delta)\left(f_{\overleftarrow{d}}(0)\alpha^{t-m+1} - f_{\overleftarrow{d}}(0)\alpha^{t-n+1}\right)}{6(f_{\overrightarrow{s}}(0) + f_{\overleftarrow{d}}(0))\alpha^t} \\
&= 1 + \frac{f_{\overleftarrow{d}}(0)(1-\delta)\left(\alpha^{n-m} - 1\right)}{6(f_{\overrightarrow{s}}(0) + f_{\overleftarrow{d}}(0))\alpha^{n-1}} \\
&= 1 + \frac{f_{\overleftarrow{d}}(0)(1-\delta)}{6(f_{\overrightarrow{s}}(0) + f_{\overleftarrow{d}}(0))}\left(\frac{1}{\alpha^{m-1}} - \frac{1}{\alpha^{n-1}}\right)
\end{aligned}$$

Using $n > m$, this gives us

$$\gamma_s(t) \geq 1 + \frac{f_{\overleftarrow{d}}(0)(1-\delta)\left(\alpha - 1\right)}{6(f_{\overrightarrow{s}}(0) + f_{\overleftarrow{d}}(0))\alpha^m}$$

Similarly, we get

$$\gamma_d(t) \geq 1 + \frac{f_{\overrightarrow{s}}(0)(1-\delta)\left(\alpha - 1\right)}{6(f_{\overrightarrow{s}}(0) + f_{\overleftarrow{d}}(0))\alpha^m}$$

We define

$$C_{f_{\overrightarrow{s}}(0), f_{\overleftarrow{d}}(0), \delta, \alpha, m} \stackrel{\text{def}}{=} 1 + \frac{min(f_{\overrightarrow{s}}(0), f_{\overleftarrow{d}}(0))(1-\delta)\left(\alpha - 1\right)}{6(f_{\overrightarrow{s}}(0) + f_{\overleftarrow{d}}(0))\alpha^m}$$

It is easy to see that $\gamma_d(t) \geq C_{f_{\overrightarrow{s}}(0), f_{\overleftarrow{d}}(0), \delta, \alpha, m}$, $\gamma_s(t) \geq C_{f_{\overrightarrow{s}}(0), f_{\overleftarrow{d}}(0), \delta, \alpha, m}$, and $C_{f_{\overrightarrow{s}}(0), f_{\overleftarrow{d}}(0), \delta, \alpha, m} > 1$, for all $t \geq L + T_1$. We know that $\gamma(t) = min\{\gamma_s(t+L-1), \gamma_s(t+L-2), \cdots, \gamma_s(t), \gamma_d(t+L-1), \gamma_d(t+L-2), \cdots, \gamma_d(t)\}$. This implies $\gamma(t) \geq C_{f_{\overrightarrow{s}}(0), f_{\overleftarrow{d}}(0), \delta, \alpha, n, m}$ which completes the proof. □

Lemma 12. *In the additive increase case, that is, when $f_{\underset{s}{\rightarrow}}(t) = f_{\underset{s}{\rightarrow}}(0) + \alpha t$ and $f_{\underset{d}{\leftarrow}}(t) = f_{\underset{d}{\rightarrow}}(0) + \alpha t$ for some $\alpha > 0$,*

$$\gamma(t) \geq 1 + \frac{\alpha(1-\delta)}{6(f_{\underset{s}{\rightarrow}}(0) + f_{\underset{d}{\leftarrow}}(0) + 2\alpha L + 2\alpha t)}$$

for all $t \geq L + T_1$.

Proof. From Lemma 10, we know that

$$\begin{aligned}
\gamma_s(t) \geq & \ 1 + \frac{(1-\delta)\left(f_{\underset{d}{\leftarrow}}(t-m+1) - f_{\underset{d}{\leftarrow}}(t-n+1)\right)}{6(f_{\underset{s}{\rightarrow}}(t) + f_{\underset{d}{\leftarrow}}(t))} \\
&= 1 + \frac{(1-\delta)\left(\alpha(n-m)\right)}{6(f_{\underset{s}{\rightarrow}}(0) + f_{\underset{d}{\leftarrow}}(0) + 2\alpha t)} \\
&\geq 1 + \frac{\alpha(1-\delta)}{6(f_{\underset{s}{\rightarrow}}(0) + f_{\underset{d}{\leftarrow}}(0) + 2\alpha t)}
\end{aligned}$$

Similarly, we get

$$\gamma_d(t) \geq 1 + \frac{\alpha(1-\delta)}{6(f_{\underset{s}{\rightarrow}}(0) + f_{\underset{d}{\leftarrow}}(0) + 2\alpha t)}$$

We know that $\gamma(t) = min\{\gamma_s(t+L-1), \gamma_s(t+L-2), \cdots, \gamma_s(t), \gamma_d(t+L-1), \gamma_d(t+L-2), \cdots, \gamma_d(t)\}$. This gives us

$$\gamma(t) \geq 1 + \frac{\alpha(1-\delta)}{6(f_{\underset{s}{\rightarrow}}(0) + f_{\underset{d}{\leftarrow}}(0) + 2\alpha L + 2\alpha t)}$$

□

Step 3. In this step, we use the lower bounds on $\gamma(t)$ shown in step 2, to find the rate of convergence.

Lemma 13. *Consider the multiplicative increase case, that is, when $f_{\underset{s}{\rightarrow}}(t) = \alpha^t f_{\underset{s}{\rightarrow}}(0)$ and $f_{\underset{d}{\leftarrow}}(t) = \alpha^t f_{\underset{d}{\rightarrow}}(0)$ for some $\alpha > 1$. For all $t \geq L + T_1 + T_2$, $\overline{p}_{\underset{uv}{\rightarrow}}(t) \geq 1 - \epsilon$ and $\overline{p}_{\underset{uv}{\leftarrow}}(t) \geq 1 - \epsilon$, for all $(u, v) \in P_1$. Here,*

$$T_1 = \max_{(u,v)\in E}\left(\frac{log\left(\frac{p_{uv}(0)}{f_{\underset{s}{\rightarrow}}(0)+f_{\underset{d}{\leftarrow}}(0)}\right)}{log\left(\frac{1}{\delta}\right)}\right)$$

$$T_2 = L\frac{log\left(\frac{2}{\epsilon r_{min}(L)}\right)}{log\left(C_{f_{\underset{s}{\rightarrow}}(1), f_{\underset{d}{\leftarrow}}(1), \delta, \alpha, m}\right)} \leq \frac{12(f_{\underset{s}{\rightarrow}}(0) + f_{\underset{d}{\leftarrow}}(0))}{(1-\delta)min(f_{\underset{s}{\rightarrow}}(0), f_{\underset{d}{\leftarrow}}(0))}\frac{\alpha^m L}{\alpha - 1}log\left(\frac{2}{\epsilon r_{min}(L)}\right)$$

Proof. In step 1, we show that $r_{min}(t)$ is non-decreasing for $t \geq L$, we know that $r_{min}(L + T_1) \geq r_{min}(L)$. And from step 2, we know that $r_{min}(t)$ increases at least by a factor of $C_{f_{\underset{s}{\rightarrow}}(0), f_{\underset{d}{\leftarrow}}(0), \delta, \alpha, n, m}$ every L time steps, for all $t \geq L + T_1$. For clarity of writing, we use C to denote $C_{f_{\underset{s}{\rightarrow}}(0), f_{\underset{d}{\leftarrow}}(0), \delta, \alpha, m}$ in the rest of this proof. This gives us

$$r_{min}(t) \geq r_{min}(L)C^{\lfloor \frac{t-L-T_1}{L} \rfloor}$$

Let $T_2 = L\frac{log\left(\frac{2}{\epsilon r_{min}(L)}\right)}{log(C)}$. For $t \geq L + T_1 + T_2$ time steps, we would get that $r_{min} \geq \frac{2}{\epsilon}$, which implies $\overline{p}_{\overrightarrow{ss_1}}(t) \geq 1-\epsilon$ and $\overline{p}_{\overleftarrow{d_1 d}}(t) \geq 1-\epsilon$. Since the pheromone level on all edges on P_1 is always non-zero, we trivially know that $\overline{p}_{\overrightarrow{uv}}(t) = 1$ for $(u,v) \in P_1 \setminus (s, s_1)$ and $\overline{p}_{\overleftarrow{uv}}(t) = 1$ for $(u,v) \in P_1 \setminus (d_1, d)$. This gives us that for $t \geq L + T_1 + T_2$, normalized pheromone level on all edges on P_1 are at least $1-\epsilon$.

To get the desired upper bound on T_2, we use $\frac{x}{1+x} \leq log(1+x)$ for all $x \geq -1$. Using this we can write

$$\begin{aligned}\frac{1}{log(C)} &= \frac{1}{log\left(1+\frac{min(f_{\overrightarrow{s}}(0), f_{\overleftarrow{d}}(0))(1-\delta)(\alpha-1)}{6(f_{\overrightarrow{s}}(0)+f_{\overleftarrow{d}}(0))\alpha^m}\right)} \\ &\leq 1 + \frac{6(f_{\overrightarrow{s}}(0)+f_{\overleftarrow{d}}(0))\alpha^m}{min(f_{\overrightarrow{s}}(0), f_{\overleftarrow{d}}(0))(1-\delta)\,(\alpha-1)} \\ &\leq \frac{12(f_{\overrightarrow{s}}(0)+f_{\overleftarrow{d}}(0))\alpha^m}{min(f_{\overrightarrow{s}}(0), f_{\overleftarrow{d}}(0))(1-\delta)\,(\alpha-1)}\end{aligned}$$

Substituting this in the expression for T_2, we get the desired bound. □

Note that the expression for T_2 involves a $\frac{\alpha^m}{\alpha-1}$ term. So if α is a constant, this would give exponential dependence on the length of the shortest path m. But by setting $\alpha \approx 1 + \frac{1}{m}$, we would get $\frac{\alpha^m}{\alpha-1} \approx m$ (for m large enough), making the dependence on the length of the shortest path linear.

Lemma 14. *Consider the additive increase case, that is, when $f_{\overrightarrow{s}}(t) = f_{\overrightarrow{s}}(0) + \alpha t$ and $f_{\overleftarrow{d}}(t) = f_{\overrightarrow{d}}(0) + \alpha t$ for some $\alpha > 0$. For all $t \geq (L + T_1 + T_2)\left(\frac{2}{r_{min}(L)\epsilon}\right)^{C_{\delta,L}}$, $\overline{p}_{\overrightarrow{uv}}(t) \geq 1-\epsilon$ and $\overline{p}_{\overleftarrow{uv}}(t) \geq 1-\epsilon$, for all $(u,v) \in P_1$. Here,*

$$T_1 = \max_{(u,v)\in E}\left(\frac{log\left(\frac{p_{uv}(0)}{f_{\overrightarrow{s}}(0)+f_{\overleftarrow{d}}(0)}\right)}{log\left(\frac{1}{\delta}\right)}\right)$$

$$T_2 = \frac{2\alpha L + f_{\overrightarrow{s}}(0) + f_{\overleftarrow{d}}(0)}{2\alpha}$$

Proof. From step 2, we know that $r_{min}(t)$ increases at least by a factor of $\gamma(t)$ every L time steps for $t \geq L + T_1$, where

$$\gamma(t) \geq 1 + \frac{\alpha(1-\delta)}{6(f_{\overrightarrow{s}}(0) + f_{\overleftarrow{d}}(0) + 2\alpha L + 2\alpha t)}$$

Note that for $t \geq T_2$,

$$\begin{aligned}\gamma(t) &\geq 1 + \frac{\alpha(1-\delta)}{6(4\alpha t)} \\ &= 1 + \frac{C_\delta}{t}\end{aligned}$$

where we define $C_\delta \stackrel{\text{def}}{=} \frac{(1-\delta)}{24}$. Using $1 + x \geq exp(\frac{x}{2})$ for $0 < x < 1$, we get

$$\gamma(t) \geq exp\left(\frac{C_\delta}{2t}\right)$$

This gives us that $r_{min}(t)$ increases at least by a factor of $exp\left(\frac{C_\delta}{2t}\right)$ every L time steps for $t \geq L + T_1 + T_2$. In step 1, we show that $r_{min}(t)$ is non-decreasing for $t \geq L$, so we know that $r_{min}(L + T_1 + T_2) \geq r_{min}(L)$. Define $C_{\delta,L} = \frac{2L}{C_\delta}$ and $T \stackrel{\text{def}}{=} L + T_1 + T_2$. Also, suppose t is of the form $T + kL$ for some positive integer k. Then this gives us

$$
\begin{aligned}
r_{min}(t) &\geq r_{min}(L)\left(exp\left(\frac{C_\delta}{2T}\right)exp\left(\frac{C_\delta}{2(T+L)}\right)\cdots exp\left(\frac{C_\delta}{2(t-L)}\right)\right)\\
&= r_{min}(L)exp\left(\frac{C_\delta}{2L}\sum_{i=0}^{\frac{t-T}{L}-1}\frac{1}{\frac{T}{L}+i}\right)\\
&\geq r_{min}(L)exp\left(\frac{C_\delta}{2L}log\left(\frac{t}{T}\right)\right)\\
&= r_{min}(L)exp\left(\frac{1}{C_{\delta,L}}log\left(\frac{t}{T}\right)\right)
\end{aligned}
$$

where we used $\sum_{i=0}^{x-1}\frac{1}{c+i} \geq log(\frac{x+c}{c})$ for the last inequality. From here, we get that for $t \geq T\left(\frac{2}{r_{min}(L)\epsilon}\right)^{C_{\delta,L}}$, $r_{min}(t) \geq \frac{2}{\epsilon}$. In the calculations above, we also assumed that t is of the form $T + kL$ for some positive integer k. But as we know that $r_{min}(t)$ is monotonically increasing for all $t \geq L$, $r_{min}(t) \geq \frac{2}{\epsilon}$ holds for all $t \geq T\left(\frac{2}{r_{min}(L)\epsilon}\right)^{C_{\delta,L}}$. This implies $\overline{p}_{\overrightarrow{ss_1}}(t) \geq 1-\epsilon$ and $\overline{p}_{\overleftarrow{d_1d}}(t) \geq 1-\epsilon$. Since the pheromone level on all edges on P_1 is always non-zero, we trivially know that $\overline{p}_{\overrightarrow{uv}}(t) = 1$ for $(u,v) \in P_1 \setminus (s,s_1)$ and $\overline{p}_{\overleftarrow{uv}}(t) = 1$ for $(u,v) \in P_1 \setminus (d_1,d)$. This gives us that for $t \geq T\left(\frac{2}{r_{min}(L)\epsilon}\right)^{C_{\delta,L}}$, normalized pheromone level on all edges on P_1 are at least $1-\epsilon$. □

This completes the proof of Theorem 2.

A.3 Connecting leakage and number of vertices

Here, we show that for any graph (not necessarily the one with parallel paths), the path with minimum leakage also has approximately the minimum number of vertices as long as variation in leakage between different vertices is not too large. (The leakage we consider in the lemma below are only for non-terminal vertices. By convention, we do not have leakage at terminal vertices in our model.)

Lemma 15. *Suppose for all pairs of vertices u and v, $\log(1-l_u)$ and $log(1-l_v)$ are within a $(1+\epsilon)$ factor of each other, where $l_v \in (0,1)$ for all v. Then the path with the minimum leakage has number of vertices at most $(1+\epsilon)$ times the path with the minimum number of vertices.*

Proof. Let $\max_v\ (1-l_v) = \alpha$ for some $\alpha \in (0,1)$. Then, since $\log(1-l_u)$ and $\log(1-l_v)$ are within a factor of $(1+\epsilon)$ for all pairs of vertices u and v, we get

$$\min_v\ (1-l_v) \geq \alpha^{1+\epsilon}.$$

Let P_1 be the path with the minimum number of vertices between s and d. And let the number of vertices on P_1 between equal to m (not counting s and d). Let P_2 be the path with the minimum leakage among all paths between s and d, and let the number of vertices on P_2 be equal to n (not counting s and d).

Then, by the bounds on leakage, we get bounds on path leakage for P_1 and P_2,

$$l_{P_1} \leq 1 - \alpha^{m(1+\epsilon)},$$
$$l_{P_2} \geq 1 - \alpha^{n}.$$

Since $l_{P_2} \leq l_{P_1}$ (P_2 is the path with the minimum leakage), we get

$$\alpha^n \geq \alpha^{m(1+\epsilon)},$$

which implies

$$m(1+\epsilon)\log\left(\frac{1}{\alpha}\right) \geq n\log\left(\frac{1}{\alpha}\right),$$

which gives

$$n \leq m(1+\epsilon).$$

Therefore, the path with the minimum leakage has number of vertices at most $(1+\epsilon)$ times the path with the minimum number of vertices. □

We do not consider the degenerate case of $l_v = 0$ or $l_v = 1$ in the above lemma as in that case, the condition on leakage would imply that either all vertices have leakage 0 in which case all paths have leakage 0, or all vertices have leakage 1 in which case all paths have leakage 1. Our claims on the effect of leakage are only relevant and interesting when we are not in these degenerate cases.

B Characterization of other rules

B.1 Necessity of bidirectional flow (Theorem 3)

Theorem 3. *Consider any graph G with two parallel paths between s and d. Let there be unidirectional flow from s to d. For any decision rule in $\mathcal{G}$, any setting of leakage parameters and with arbitrary incoming flow levels, there exists a setting of initial pheromone levels, such that the dynamics does not converge to the shortest or the minimum leakage path.*

Proof of Theorem 3. Let P_1 and P_2 denote be two parallel paths between s and d, and s_1 and s_2 be neighboring vertices of s on P_1 and P_2 respectively. Without loss of generality, let P_1 be the minimum leakage or the shortest path, and let the flow be unidirectional from s to d.

As the flow is unidirectional, we get that the pheromone levels on the edges incident on s is only a function of their initial pheromone levels and forward flow at s. That is, for some function F, $p_{ss_1}(t)$ and $p_{ss_2}(t)$ can be written as

$$p_{ss_1}(t) = F(p_{ss_1}(0), p_{ss_2}(0), f_{\vec{s}}(0), f_{\vec{s}}(1), \ldots f_{\vec{s}}(t-1))$$
$$p_{ss_2}(t) = F(p_{ss_2}(0), p_{ss_1}(0), f_{\vec{s}}(0), f_{\vec{s}}(1), \ldots f_{\vec{s}}(t-1))$$

Given $(p_{ss_1}(0), p_{ss_2}(0))$, and $f_{\vec{s}}(t)$ for all $t \geq 0$, suppose the dynamics converges to P_1. Now if we swap the initial pheromone level on the two edges incident to s, then the dynamics would converge to P_2. Therefore, for one of these two initial pheromone settings, the dynamics does not converge to the minimum leakage or the shortest path. □

B.2 Necessity of the linear rule for convergence to the minimum leakage path (Theorem 4)

Theorem 4. *Consider any graph G consisting of two parallel paths from s to d. When the incoming flow is fixed, for every non-linear decision rule $g \in \mathcal{F}$, there exists a setting of leakage parameters and initial pheromone and flow levels dependent on g, such that the dynamics does not converge to the path with the minimum leakage.*

Proof of Theorem 4. For notational convenience, we define $\alpha = 1 - l_{P_1}$ and $\beta = 1 - l_{P_2}$. Without loss of generality we assume P_1 to be the path of minimum leakage and therefore we let $\alpha \geq \beta$. Let n and m be the number of vertices between s and d on paths P_1 and P_2 respectively. We name the vertices from left to right on path P_1 by v_0 to v_{n+1} and P_2 by u_0 to u_{m+1}, with the convention $v_0 = u_0 = s$ and $v_{n+1} = u_{m+1} = d$. We also let $s_1 = v_1, s_2 = u_1, d_1 = v_n$ and $d_2 = u_m$. Let $f_{\overrightarrow{s}}$ and $f_{\overleftarrow{d}}$ denote the fixed incoming forward and backward flow. We divide the analysis into two cases. For any non-linear $g \in \mathcal{F}$, there exists an $r \in (0, 1/2)$ such that $g(r) \neq r$. For such an r, one of the following two conditions holds:

- $g(r) < r$.
- $g(r) > r$.

Case 1: Suppose $g(r) < r$, then pick the following initial configuration:

- Assign pheromone value on the edges $(s, s_1), (s, s_2), (d_1, d), (d_2, d)$ such that the normalized pheromone level on $(s, s_1), (d_1, d)$ is $\leq r$, that is $\overline{p}_{\overrightarrow{ss_1}}(0), \overline{p}_{\overleftarrow{d_1 d}}(0) \leq r$. Further assign the flow values on the edges such that they satisfy the following,

$$f_{\overrightarrow{v_i v_{i+1}}}(0) \leq f_{\overrightarrow{s}} \cdot r \cdot \prod_{j \leq i}(1 - l_{v_j}) \text{ and } f_{\overleftarrow{v_i v_{i+1}}}(0) \leq f_{\overleftarrow{d}} \cdot r \cdot \prod_{j \geq i+1}(1 - l_{v_j}) \tag{79}$$

$$f_{\overrightarrow{u_i u_{i+1}}}(0) \geq f_{\overrightarrow{s}} \cdot (1 - r) \cdot \prod_{j \leq i}(1 - l_{u_j}) \text{ and } f_{\overleftarrow{u_i u_{i+1}}}(0) \geq f_{\overleftarrow{d}} \cdot (1 - r) \cdot \prod_{j \geq i+1}(1 - l_{u_j}) \,. \tag{80}$$

  (Since we do not assume any leakage at the terminal vertices while defining the path leakage (see definition 1), we set $l_{v_0} = l_{u_0} = l_{v_{n+1}} = l_{u_{m+1}} = 0$ in the expressions above.)

- Let the leakage value at each vertex be such that the parameters α and β satisfy the following inequality, $\frac{\alpha}{\beta} \leq 1 + \frac{c_{g,r}}{4 \cdot r} \cdot \min\left(\frac{f_{\overrightarrow{s}}}{f_{\overleftarrow{d}}}, \frac{f_{\overleftarrow{d}}}{f_{\overrightarrow{s}}}\right)$, where $c_{g,r} \stackrel{\text{def}}{=} r - g(r) > 0$. As the forward and backward flow is fixed, the above constraint on the leakage parameters only depend on g and therefore satisfy the conditions of the theorem.

We show by induction that the above inequalities on flow and pheromone levels hold for all time $t \geq 0$ and therefore the system does not converge to the minimum leakage path.

Hypothesis: At time $t \geq 0$, pheromone values on the edges $(s, s_1), (s, s_2), (d_1, d), (d_2, d)$ are such that the normalized pheromone level on $(s, s_1), (d_1, d)$ is $\leq r$, that is $\overline{p}_{\overrightarrow{ss_1}}(t), \overline{p}_{\overleftarrow{d_1 d}}(t) \leq r$. Further the flow values on the edges satisfy the following,

$$f_{\overrightarrow{v_i v_{i+1}}}(t) \leq f_{\overrightarrow{s}} \cdot r \cdot \prod_{j \leq i}(1 - l_{v_j}) \text{ and } f_{\overleftarrow{v_i v_{i+1}}}(t) \leq f_{\overleftarrow{d}} \cdot r \cdot \prod_{j \geq i+1}(1 - l_{v_j}) \tag{81}$$

$$f_{\overrightarrow{u_i u_{i+1}}}(t) \geq f_{\overrightarrow{s}} \cdot (1 - r) \cdot \prod_{j \leq i}(1 - l_{u_j}) \text{ and } f_{\overleftarrow{u_i u_{i+1}}}(t) \geq f_{\overleftarrow{d}} \cdot (1 - r) \cdot \prod_{j \geq i+1}(1 - l_{u_j}) \,. \tag{82}$$

Base case: The conditions trivially hold at time 0 because of the initial setting of flow and pheromone levels described above.

Induction Step: To prove the hypothesis for time $t+1$, all we need to show is that, $\overline{p}_{\overrightarrow{ss_1}}(t+1), \overline{p}_{\overleftarrow{d_1 d}}(t+1) \leq r$, $f_{\overrightarrow{v_0 v_1}}(t+1) = f_{\overrightarrow{ss_1}}(t+1) \leq f_{\overrightarrow{s}} \cdot r$ and $f_{\overleftarrow{v_n v_{n+1}}}(t+1) = f_{\overleftarrow{d_1 d}}(t+1) \leq f_{\overleftarrow{d}} \cdot r$; all the remaining inequalities follow from these basic inequalities. Also note that from the definition of our case, that is $g(r) < r$, we get that the inequalities $\overline{p}_{\overrightarrow{ss_1}}(t+1), \overline{p}_{\overleftarrow{d_1 d}}(t+1) \leq r$ further imply the following,

$$f_{\overrightarrow{ss_1}}(t+1) = f_{\overrightarrow{s}} \cdot g(\overline{p}_{\overrightarrow{ss_1}}(t+1)) \leq f_{\overrightarrow{s}} \cdot g(r) = f_{\overrightarrow{s}} \cdot r \ ,$$

and similarly,

$$f_{\overleftarrow{d_1 d}}(t+1) < f_{\overleftarrow{d}} \cdot r \ .$$

In the above we used monotonically non-decreasing property of decision rule g. Therefore it is enough to show that $\overline{p}_{\overrightarrow{ss_1}}(t+1), \overline{p}_{\overleftarrow{d_1 d}}(t+1) \leq r$. As the proof for the bound on $\overline{p}_{\overleftarrow{d_1 d}}(t+1)$ is analogous to that of $\overline{p}_{\overrightarrow{ss_1}}(t+1)$, in the remainder we focus our attention towards the proof for $\overline{p}_{\overrightarrow{ss_1}}(t+1)$. Recall the definition of $\overline{p}_{\overrightarrow{ss_1}}(t+1)$,

$$\overline{p}_{\overrightarrow{ss_1}}(t+1) = \frac{p_{ss_1}(t+1)}{p_{ss_1}(t+1) + p_{ss_2}(t+1)} = \frac{p_{ss_1}(t) + f_{\overrightarrow{ss_1}}(t) + f_{\overleftarrow{ss_1}}(t)}{p_{ss_2}(t) + p_{ss_1}(t) + f_{\overrightarrow{ss_2}}(t) + f_{\overrightarrow{ss_1}}(t) + f_{\overleftarrow{ss_2}}(t) + f_{\overleftarrow{ss_1}}(t)} \tag{83}$$

We know by the induction step that,

$$\frac{p_{ss_1}(t)}{p_{ss_2}(t) + p_{ss_1}(t)} < r \ . \tag{84}$$

Also note that,

$$\frac{f_{\overleftarrow{ss_1}}(t)}{f_{\overleftarrow{ss_2}}(t) + f_{\overleftarrow{ss_1}}(t)} = \frac{\frac{f_{\overleftarrow{ss_1}}(t)}{f_{\overleftarrow{ss_2}}(t)}}{1 + \frac{f_{\overleftarrow{ss_1}}(t)}{f_{\overleftarrow{ss_2}}(t)}} \leq \frac{\frac{r\alpha}{(1-r)\beta}}{1 + \frac{r\alpha}{(1-r)\beta}} = \frac{r\alpha}{\beta + r(\alpha - \beta)} \leq \frac{\alpha}{\beta} \cdot r \ . \tag{85}$$

In the above we used the monotonically non-decreasing property of $x/(1+x)$ and the conditions provided by the induction step at time t, that is $f_{\overleftarrow{ss_1}}(t) \leq f_{\overleftarrow{d}} \cdot r \cdot \alpha$ and $f_{\overleftarrow{ss_2}}(t) \geq f_{\overleftarrow{d}} \cdot (1-r) \cdot \beta$; which implies $\frac{f_{\overleftarrow{ss_1}}(t)}{f_{\overleftarrow{ss_2}}(t)} \leq \frac{r\alpha}{(1-r)\beta}$. In the fourth inequality, we used $\alpha - \beta \geq 0$ and $r \geq 0$.

Let $c_{g,r} \stackrel{\text{def}}{=} r - g(r) > 0$ and note that we have the following upper bound on the flow value,

$$f_{\overrightarrow{ss_1}}(t) = f_{\overrightarrow{s}} \cdot g(\overline{p}_{\overrightarrow{ss_1}}(t)) \leq f_{\overrightarrow{s}} \cdot g(r) = f_{\overrightarrow{s}} \cdot (r - c_{g,r}) \ . \tag{86}$$

In the above we used $\overline{p}_{\overrightarrow{ss_1}}(t) \leq r$ and monotonicity of decision rule g. Using these bounds, we provide an

upper bound for the normalized pheromone level.

$$\overline{p}_{\overrightarrow{ss_1}}(t+1) = \frac{p_{ss_1}(t) + f_{\overrightarrow{ss_1}}(t) + f_{\overleftarrow{ss_1}}(t)}{p_{ss_2}(t) + p_{ss_1}(t) + f_{\overrightarrow{ss_2}}(t) + f_{\overrightarrow{ss_1}}(t) + f_{\overleftarrow{ss_2}}(t) + f_{\overleftarrow{ss_1}}(t)}, \tag{87}$$

$$\leq \frac{r \cdot (p_{ss_2}(t) + p_{ss_1}(t)) + f_{\overrightarrow{ss_1}}(t) + r \cdot \frac{\alpha}{\beta} \cdot (f_{\overleftarrow{ss_2}}(t) + f_{\overleftarrow{ss_1}}(t))}{p_{ss_2}(t) + p_{ss_1}(t) + f_{\overrightarrow{ss_2}}(t) + f_{\overrightarrow{ss_1}}(t) + f_{\overleftarrow{ss_2}}(t) + f_{\overleftarrow{ss_1}}(t)}, \tag{88}$$

$$= r + \frac{r \cdot \left(\frac{\alpha}{\beta} - 1\right) \cdot (f_{\overleftarrow{ss_2}}(t) + f_{\overleftarrow{ss_1}}(t)) + f_{\overrightarrow{ss_1}}(t) - r(f_{\overrightarrow{ss_1}}(t) + f_{\overrightarrow{ss_2}}(t))}{p_{ss_2}(t) + p_{ss_1}(t) + f_{\overrightarrow{ss_2}}(t) + f_{\overrightarrow{ss_1}}(t) + f_{\overleftarrow{ss_2}}(t) + f_{\overleftarrow{ss_1}}(t)}, \tag{89}$$

$$\leq r + \frac{2rf_{\overleftarrow{d}} \cdot \left(\frac{\alpha}{\beta} - 1\right) + f_{\overrightarrow{s}} \cdot (r - c_{g,r}) - r \cdot f_{\overrightarrow{s}}}{p_{ss_2}(t) + p_{ss_1}(t) + f_{\overrightarrow{ss_2}}(t) + f_{\overrightarrow{ss_1}}(t) + f_{\overleftarrow{ss_2}}(t) + f_{\overleftarrow{ss_1}}(t)}, \tag{90}$$

$$\leq r + \frac{2rf_{\overleftarrow{d}} \cdot \left(\frac{\alpha}{\beta} - 1\right) - c_{g,r}f_{\overrightarrow{s}}}{p_{ss_2}(t) + p_{ss_1}(t) + f_{\overrightarrow{ss_2}}(t) + f_{\overrightarrow{ss_1}}(t) + f_{\overleftarrow{ss_2}}(t) + f_{\overleftarrow{ss_1}}(t)}, \tag{91}$$

$$\leq r - \frac{\frac{1}{2}c_{g,r} \cdot f_{\overrightarrow{s}}}{p_{ss_2}(t) + p_{ss_1}(t) + f_{\overrightarrow{ss_2}}(t) + f_{\overrightarrow{ss_1}}(t) + f_{\overleftarrow{ss_2}}(t) + f_{\overleftarrow{ss_1}}(t)}, \tag{92}$$

$$\leq r \,. \tag{93}$$

In the second inequality we used Equations (84) and (85). In the third equality, we rearranged the terms. In the fourth inequality, we used $f_{\overleftarrow{ss_2}}(t), f_{\overleftarrow{ss_1}}(t) \leq f_{\overleftarrow{d}}$, $f_{\overrightarrow{ss_1}}(t) + f_{\overrightarrow{ss_2}}(t) = f_{\overrightarrow{s}}$ and Equation (86). In the fifth inequality, we simplified the expression. The sixth inequality follows because α, β, $f_{\overrightarrow{s}}$ and $f_{\overleftarrow{d}}$ satisfy $\frac{\alpha}{\beta} \leq 1 + \frac{c_{g,r}}{4 \cdot r} \cdot \min\left(\frac{f_{\overrightarrow{s}}}{f_{\overleftarrow{d}}}, \frac{f_{\overleftarrow{d}}}{f_{\overrightarrow{s}}}\right)$. Therefore, the previous derivation gives us,

$$\overline{p}_{\overrightarrow{ss_1}}(t+1) \leq r \,,$$

and we conclude the first case.

Case 2: Suppose $g(r) > r$, then pick the following initial configuration:

- Assign pheromone value on the edges $(s, s_1), (s, s_2), (d_1, d), (d_2, d)$ such that the normalized pheromone level on $(s, s_2), (d_2, d)$ is $\geq r$, that is $\overline{p}_{\overrightarrow{ss_2}}(0), \overline{p}_{\overleftarrow{d_2 d}}(0) \geq r$. Further assign the flow values on the edges such that they satisfy the following,

$$f_{\overrightarrow{v_i v_{i+1}}}(0) \leq f_{\overrightarrow{s}} \cdot (1-r) \cdot \prod_{j \leq i}(1 - l_{v_j}) \text{ and } f_{\overleftarrow{v_i v_{i+1}}}(0) \leq f_{\overleftarrow{d}} \cdot (1-r) \cdot \prod_{j \geq i+1}(1 - l_{v_j}) \tag{94}$$

$$f_{\overrightarrow{u_i u_{i+1}}}(0) \geq f_{\overrightarrow{s}} \cdot r \cdot \prod_{j \leq i}(1 - l_{u_j}) \text{ and } f_{\overleftarrow{u_i u_{i+1}}}(0) \geq f_{\overleftarrow{d}} \cdot r \cdot \prod_{j \geq i+1}(1 - l_{u_j}) \,. \tag{95}$$

  (Since we do not assume any leakage at the terminal vertices while defining the path leakage (see definition 1), we set $l_{v_0} = l_{u_0} = l_{v_{n+1}} = l_{u_{m+1}} = 0$ in the expressions above.)

- Let the leakage value at each vertex be such that the parameters α and β satisfy the following inequality, $1 \geq \frac{\beta}{\alpha} \geq 1 - \frac{c_{g,r}}{4 \cdot r} \cdot \min\left(\frac{f_{\overrightarrow{s}}}{f_{\overleftarrow{d}}}, \frac{f_{\overleftarrow{d}}}{f_{\overleftarrow{s}}}\right)$, where $c_{g,r} \stackrel{\text{def}}{=} g(r) - r > 0$. As the forward and backward flow is fixed, the above constraint on the leakage parameters only depend on g and therefore satisfy the conditions of the theorem.

We show by induction that the above inequalities on flow and pheromone levels hold for all time $t \geq 0$ and therefore the system does not converge to the minimum leakage path.

Hypothesis: At time $t \geq 0$, pheromone values on the edges $(s,s_1),(s,s_2),(d_1,d),(d_2,d)$ are such that the normalized pheromone level on $(s,s_2),(d_2,d)$ is $\geq r$, that is $\overline{p}_{\overrightarrow{ss_2}}(t), \overline{p}_{\overleftarrow{d_2d}}(t) \geq r$. Further the flow values on the edges satisfy the following,

$$f_{\overrightarrow{v_iv_{i+1}}}(t) \leq f_{\overrightarrow{s}} \cdot (1-r) \cdot \prod_{j\leq i}(1-l_{v_j}) \text{ and } f_{\overleftarrow{v_iv_{i+1}}}(t) \leq f_{\overleftarrow{d}} \cdot (1-r) \cdot \prod_{j\geq i+1}(1-l_{v_j}) \tag{96}$$

$$f_{\overrightarrow{u_iu_{i+1}}}(t) \geq f_{\overrightarrow{s}} \cdot r \cdot \prod_{j\leq i}(1-l_{u_j}) \text{ and } f_{\overleftarrow{u_iu_{i+1}}}(t) \geq f_{\overleftarrow{d}} \cdot r \cdot \prod_{j\geq i+1}(1-l_{u_j}) \; . \tag{97}$$

Base case: The conditions trivially hold at time 0 because of the initial setting of flow and pheromone levels described above.

Induction Step: To prove the hypothesis for time $t+1$, all we need to show is that, $\overline{p}_{\overrightarrow{ss_2}}(t+1), \overline{p}_{\overleftarrow{d_2d}}(t+1) \geq r$, $f_{\overrightarrow{u_0u_1}}(t+1) = f_{\overrightarrow{ss_2}}(t+1) \geq f_{\overrightarrow{s}} \cdot r$ and $f_{\overleftarrow{u_mu_{m+1}}}(t+1) = f_{\overleftarrow{d_2d}}(t+1) \geq f_{\overleftarrow{d}} \cdot r$; all the remaining inequalities follows from these basic inequalities. Also, from the definition of our case, that is $g(r) > r$, we get that the inequalities $\overline{p}_{\overrightarrow{ss_2}}(t+1), \overline{p}_{\overleftarrow{d_2d}}(t+1) \geq r$ further imply $f_{\overrightarrow{ss_2}}(t+1) \geq f_{\overrightarrow{s}} \cdot r$ and $f_{\overleftarrow{d_2d}}(t+1) \geq f_{\overleftarrow{d}} \cdot r$.

To see this, note that

$$f_{\overrightarrow{ss_2}}(t+1) = f_{\overrightarrow{s}} \cdot g(\overline{p}_{\overrightarrow{ss_2}}(t+1)) \geq f_{\overrightarrow{s}} \cdot g(r) \geq f_{\overrightarrow{s}} \cdot r \; ,$$

when $\overline{p}_{\overrightarrow{ss_2}}(t+1) \leq 1/2$ and,

$$f_{\overrightarrow{ss_2}}(t+1) = f_{\overrightarrow{s}} \cdot (1 - g(1 - \overline{p}_{\overrightarrow{ss_2}}(t+1))) \geq f_{\overrightarrow{s}} \cdot (1 - g(1/2)) = f_{\overrightarrow{s}} \cdot \frac{1}{2} \geq f_{\overrightarrow{s}} \cdot r \; ,$$

when $\overline{p}_{\overrightarrow{ss_2}}(t+1) \geq 1/2$, where we used monotonically non-decreasing property of g for the above inequalities. Also, we used $g(1/2) = 1/2$ and $r \leq 1/2$ for the last inequality. Here, we had to consider two cases because function g takes as input the minimum of the two normalized pheromone levels. Similarly we can show $f_{\overleftarrow{d_2d}}(t+1) \geq f_{\overleftarrow{d}} \cdot r$.

Therefore it is enough to show that $\overline{p}_{\overrightarrow{ss_2}}(t+1), \overline{p}_{\overleftarrow{d_2d}}(t+1) \geq r$. As the proof for the bound on $\overline{p}_{\overleftarrow{d_2d}}(t+1)$ is analogous to that of $\overline{p}_{\overrightarrow{ss_2}}(t+1)$, in the remainder we focus our attention on the proof for $\overline{p}_{\overrightarrow{ss_2}}(t+1)$. Recall the definition of $\overline{p}_{\overrightarrow{ss_2}}(t+1)$,

$$\overline{p}_{\overrightarrow{ss_2}}(t+1) = \frac{p_{ss_2}(t+1)}{p_{ss_1}(t+1) + p_{ss_2}(t+1)} = \frac{p_{ss_2}(t) + f_{\overrightarrow{ss_2}}(t) + f_{\overleftarrow{ss_2}}(t)}{p_{ss_1}(t) + p_{ss_2}(t) + f_{\overrightarrow{ss_1}}(t) + f_{\overrightarrow{ss_2}}(t) + f_{\overleftarrow{ss_1}}(t) + f_{\overleftarrow{ss_2}}(t)} \tag{98}$$

We know by the induction step that,

$$\frac{p_{ss_2}(t)}{p_{ss_1}(t) + p_{ss_2}(t)} \geq r \; . \tag{99}$$

Also note that,

$$\frac{f_{\overleftarrow{ss_2}}(t)}{f_{\overleftarrow{ss_1}}(t) + f_{\overleftarrow{ss_2}}(t)} = \frac{\frac{f_{\overleftarrow{ss_2}}(t)}{f_{\overleftarrow{ss_1}}(t)}}{1 + \frac{f_{\overleftarrow{ss_2}}(t)}{f_{\overleftarrow{ss_1}}(t)}} \geq \frac{\frac{r\beta}{(1-r)\alpha}}{1 + \frac{r\beta}{(1-r)\alpha}} = \frac{r\beta}{\alpha - r(\alpha - \beta)} \geq \frac{\beta}{\alpha} \cdot r \; . \tag{100}$$

In the above we used the monotonically non-decreasing property of $x/(1+x)$ and the conditions provided by the induction step at time t, that is $f_{\overleftarrow{ss_2}}(t) \geq f_{\overleftarrow{d}} \cdot r \cdot \beta$ and $f_{\overleftarrow{ss_1}}(t) \leq f_{\overleftarrow{d}} \cdot (1-r) \cdot \alpha$; which implies $\frac{f_{\overleftarrow{ss_2}}(t)}{f_{\overleftarrow{ss_1}}(t)} \geq \frac{r\beta}{(1-r)\alpha}$. In the fourth inequality, we used $\alpha - \beta \geq 0$ and $1 \geq r \geq 0$.

Let $c_{g,r} \stackrel{\text{def}}{=} g(r) - r > 0$. We have the following upper bound on the flow value,

$$f_{\overrightarrow{ss_2}}(t) \geq f_{\overrightarrow{s}} \cdot (r + c_{g,r}) \;, \tag{101}$$

To see this, note that

$$f_{\overrightarrow{ss_2}}(t) = f_{\overrightarrow{s}} \cdot g(\overline{p}_{\overrightarrow{ss_2}}(t)) \geq f_{\overrightarrow{s}} \cdot g(r) = f_{\overrightarrow{s}} \cdot (r + c_{g,r}) \;,$$

when $\overline{p}_{\overrightarrow{ss_2}}(t) \leq 1/2$. In the above we used $\overline{p}_{\overrightarrow{ss_2}}(t) \geq r$ and monotonicity of decision rule g. And

$$f_{\overrightarrow{ss_2}}(t) = f_{\overrightarrow{s}} \cdot (1 - g(1 - \overline{p}_{\overrightarrow{ss_2}}(t))) \geq f_{\overrightarrow{s}} \cdot (1 - g(1/2)) = f_{\overrightarrow{s}} \cdot \frac{1}{2} = f_{\overrightarrow{s}} \cdot g(1/2) \geq f_{\overrightarrow{s}} \cdot g(r) = f_{\overrightarrow{s}} \cdot (r + c_{g,r}) \;,$$

when $\overline{p}_{\overrightarrow{ss_2}}(t) > 1/2$. In the above, we used $\overline{p}_{\overrightarrow{ss_2}}(t) > 1/2$, monotonicity of decision rule g, $g(1/2) = 1/2$ and $r \leq 1/2$.

Using these bounds, we provide an upper bound for the normalized pheromone level.

$$\begin{aligned}
\overline{p}_{\overrightarrow{ss_2}}(t+1) &= \frac{p_{ss_2}(t) + f_{\overrightarrow{ss_2}}(t) + f_{\overleftarrow{ss_2}}(t)}{p_{ss_1}(t) + p_{ss_2}(t) + f_{\overrightarrow{ss_1}}(t) + f_{\overrightarrow{ss_2}}(t) + f_{\overleftarrow{ss_1}}(t) + f_{\overleftarrow{ss_2}}(t)}, &(102)\\
&\geq \frac{r \cdot (p_{ss_1}(t) + p_{ss_2}(t)) + f_{\overrightarrow{ss_2}}(t) + \frac{\beta}{\alpha} \cdot r \cdot (f_{\overleftarrow{ss_1}}(t) + f_{\overleftarrow{ss_2}}(t))}{p_{ss_1}(t) + p_{ss_2}(t) + f_{\overrightarrow{ss_1}}(t) + f_{\overrightarrow{ss_2}}(t) + f_{\overleftarrow{ss_1}}(t) + f_{\overleftarrow{ss_2}}(t)} &(103)\\
&= r + \frac{(\frac{\beta}{\alpha} - 1) \cdot r \cdot (f_{\overleftarrow{ss_2}}(t) + f_{\overleftarrow{ss_1}}(t)) + \left(f_{\overrightarrow{ss_2}}(t) - r \cdot (f_{\overrightarrow{ss_1}}(t) + f_{\overrightarrow{ss_2}}(t))\right)}{p_{ss_1}(t) + p_{ss_2}(t) + f_{\overrightarrow{ss_1}}(t) + f_{\overrightarrow{ss_2}}(t) + f_{\overleftarrow{ss_1}}(t) + f_{\overleftarrow{ss_2}}(t)}, &(104)\\
&\geq r + \frac{(\frac{\beta}{\alpha} - 1) \cdot r \cdot 2f_{\overleftarrow{d}} + \left(f_{\overrightarrow{ss_2}}(t) - r \cdot f_{\overrightarrow{s}}\right)}{p_{ss_1}(t) + p_{ss_2}(t) + f_{\overrightarrow{ss_1}}(t) + f_{\overrightarrow{ss_2}}(t) + f_{\overleftarrow{ss_1}}(t) + f_{\overleftarrow{ss_2}}(t)}, &(105)\\
&\geq r + \frac{(\frac{\beta}{\alpha} - 1) \cdot r \cdot 2f_{\overleftarrow{d}} + f_{\overrightarrow{s}}\left(r + c_{g,r} - r\right)}{p_{ss_1}(t) + p_{ss_2}(t) + f_{\overrightarrow{ss_1}}(t) + f_{\overrightarrow{ss_2}}(t) + f_{\overleftarrow{ss_1}}(t) + f_{\overleftarrow{ss_2}}(t)}, &(106)\\
&= r + \frac{(\frac{\beta}{\alpha} - 1) \cdot r \cdot 2f_{\overleftarrow{d}} + c_{g,r} f_{\overrightarrow{s}}}{p_{ss_1}(t) + p_{ss_2}(t) + f_{\overrightarrow{ss_1}}(t) + f_{\overrightarrow{ss_2}}(t) + f_{\overleftarrow{ss_1}}(t) + f_{\overleftarrow{ss_2}}(t)}, &(107)\\
&\geq r + \frac{\frac{1}{2} c_{g,r} f_{\overrightarrow{s}}}{p_{ss_1}(t) + p_{ss_2}(t) + f_{\overrightarrow{ss_1}}(t) + f_{\overrightarrow{ss_2}}(t) + f_{\overleftarrow{ss_1}}(t) + f_{\overleftarrow{ss_2}}(t)} \;, &(108)\\
&\geq r \;. &(109)
\end{aligned}$$

In the second inequality we used Equations (99) and (100). In the third equality, we rearranged terms. In the fourth inequality, we used $\frac{\beta}{\alpha} \leq 1$ and $f_{\overleftarrow{ss_2}}(t), f_{\overleftarrow{ss_1}}(t) \leq f_{\overleftarrow{d}}$, $f_{\overrightarrow{ss_1}}(t) + f_{\overrightarrow{ss_2}}(t) \leq f_{\overrightarrow{s}}$ inequalities. In the fifth inequality, we used Equation (101). The seventh inequality holds because $(\frac{\beta}{\alpha} - 1) \cdot r \cdot 2f_{\overleftarrow{d}} \geq -\frac{1}{2} c_{g,r} f_{\overrightarrow{s}}$, which is equivalent to $\frac{\beta}{\alpha} \geq 1 - \frac{c_{g,r}}{4 \cdot r} \frac{f_{\overrightarrow{s}}}{f_{\overleftarrow{d}}}$. Note that this constraint is satisfied by our choice of values for leakage parameters. Further the previous derivation gives us,

$$\overline{p}_{\overrightarrow{ss_2}}(t+1) \geq r \;,$$

and we conclude the second case. □

B.3 Necessity of the linear rule for convergence to the shortest path (Theorem 5)

Theorem 5. *Consider any graph G consisting of two parallel paths from s to d with a unique shortest path. When the leakage is zero for all the vertices, for every non-linear decision rule $g \in \mathcal{F}$, there exists a setting of initial pheromone and flow levels, with incoming flow increasing by a fixed multiplicative factor at each time step, such that the dynamics does not converge to the shortest path. The multiplicative factor and initial pheromone and flow levels are chosen as a function of g.*

Proof of Theorem 5. Let n and m be the number of vertices between s and d on paths P_1 and P_2 respectively. Without loss of generality we let P_1 to be the shortest path (that is, $n < m$). We name the vertices from left to right on path P_1 by v_0 to v_{n+1} and P_2 by u_0 to u_{m+1}, with the convention $v_0 = u_0 = s$ and $v_{n+1} = u_{m+1} = d$. We also let $s_1 = v_1, s_2 = u_1, d_1 = v_n$ and $d_2 = u_m$. We divide the analysis into two cases.

For any non-linear $g \in \mathcal{F}$, there exists an $r \in (0, 1/2)$ such that $g(r) \neq r$. For such an r, one of the following two conditions holds: $g(r) < r$ or $g(r) > r$.

Case 1: Suppose $g(r) < r$, then we pick the following initial configuration and flow values:

- Assign pheromone values on the edges $(s, s_1), (s, s_2), (d_1, d), (d_2, d)$ such that the normalized pheromone level on $(s, s_1), (d_1, d)$ is $\leq r$, that is $\overline{p}_{\overrightarrow{ss_1}}(0), \overline{p}_{\overleftarrow{d_1 d}}(0) \leq r$. Further assign the flow values on the edges such that they satisfy the following,

$$f_{\overrightarrow{v_i v_{i+1}}}(0) \leq f_{\overrightarrow{s}}(0) \cdot r \text{ and } f_{\overleftarrow{v_i v_{i+1}}}(0) \leq f_{\overleftarrow{d}}(0) \cdot r \tag{110}$$

$$f_{\overrightarrow{u_i u_{i+1}}}(0) \geq f_{\overrightarrow{s}}(0) \cdot (1 - r) \text{ and } f_{\overleftarrow{u_i u_{i+1}}}(0) \geq f_{\overleftarrow{d}}(0) \cdot (1 - r) \ . \tag{111}$$

- Let $f_{\overrightarrow{s}}(0) = f_{\overleftarrow{d}}(0) = 1$. We specify the values of flow $f_{\overrightarrow{s}}(t), f_{\overleftarrow{d}}(t)$ for times $t > 0$ later in the proof.

We show by induction that the above inequalities on flow and pheromone levels hold for all time $t \geq 0$ and therefore the system does not converge to the shortest path.

Hypothesis: At time $t \geq 0$, pheromone value on the edges $(s, s_1), (s, s_2), (d_1, d), (d_2, d)$ are such that the normalized pheromone level on $(s, s_1), (d_1, d)$ is $\leq r$, that is $\overline{p}_{\overrightarrow{ss_1}}(t), \overline{p}_{\overleftarrow{d_1 d}}(t) \leq r$. Further the flow values on the edges satisfy the following,

$$f_{\overrightarrow{v_i v_{i+1}}}(t) \leq f_{\overrightarrow{s}}(t - i) \cdot r \text{ and } f_{\overleftarrow{v_i v_{i+1}}}(t) \leq f_{\overleftarrow{d}}(t - (n - i)) \cdot r \tag{112}$$

$$f_{\overrightarrow{u_i u_{i+1}}}(t) \geq f_{\overrightarrow{s}}(t - i) \cdot (1 - r) \text{ and } f_{\overleftarrow{u_i u_{i+1}}}(t) \geq f_{\overleftarrow{d}}(t - (m - i)) \cdot (1 - r) \ . \tag{113}$$

In the above equations, $f_{\overrightarrow{s}}(t') \stackrel{\text{def}}{=} f_{\overrightarrow{s}}(0)$ and $f_{\overleftarrow{d}}(t') \stackrel{\text{def}}{=} f_{\overleftarrow{d}}(0)$ for all $t' < 0$.

Base case: The conditions trivially hold at time 0 because of the initial setting of flow and pheromone levels described above.

Induction Step: To prove the hypothesis for time $t+1$, all we need to show is that, $\overline{p}_{\overrightarrow{ss_1}}(t+1), \overline{p}_{\overleftarrow{d_1d}}(t+1) \leq r$, $f_{\overrightarrow{v_0v_1}}(t+1) = f_{\overrightarrow{ss_1}}(t+1) \leq f_{\overrightarrow{s}}(t+1) \cdot r$ and $f_{\overleftarrow{v_nv_{n+1}}}(t+1) = f_{\overleftarrow{d_1d}}(t+1) \leq f_{\overleftarrow{d}}(t+1) \cdot r$; all the remaining inequalities follow from these basic inequalities. Also note that from the definition of our case, that is $g(r) < r$, we get that the inequalities $\overline{p}_{\overrightarrow{ss_1}}(t+1), \overline{p}_{\overleftarrow{d_1d}}(t+1) \leq r$ further imply the following,

$$f_{\overrightarrow{ss_1}}(t+1) = f_{\overrightarrow{s}}(t+1) \cdot g(\overline{p}_{\overrightarrow{ss_1}}(t+1)) \leq f_{\overrightarrow{s}}(t+1) \cdot g(r) < f_{\overrightarrow{s}}(t+1) \cdot r \ ,$$

and similarly,

$$f_{\overleftarrow{d_1d}}(t+1) < f_{\overleftarrow{d}}(t+1) \cdot r \ .$$

In the above we used monotonically non-decreasing property of decision rule g. Therefore it is enough to show that $\overline{p}_{\overrightarrow{ss_1}}(t+1), \overline{p}_{\overleftarrow{d_1d}}(t+1) \leq r$. As the proof for the bound on $\overline{p}_{\overleftarrow{d_1d}}(t+1)$ is analogous to that of $\overline{p}_{\overrightarrow{ss_1}}(t+1)$, in the remainder we focus our attention on the proof for $\overline{p}_{\overrightarrow{ss_1}}(t+1)$. Recall the definition of $\overline{p}_{\overrightarrow{ss_1}}(t+1)$,

$$\overline{p}_{\overrightarrow{ss_1}}(t+1) = \frac{p_{ss_1}(t+1)}{p_{ss_1}(t+1) + p_{ss_2}(t+1)} = \frac{p_{ss_1}(t) + f_{\overrightarrow{ss_1}}(t) + f_{\overleftarrow{ss_1}}(t)}{p_{ss_2}(t) + p_{ss_1}(t) + f_{\overrightarrow{ss_2}}(t) + f_{\overrightarrow{ss_1}}(t) + f_{\overleftarrow{ss_2}}(t) + f_{\overleftarrow{ss_1}}(t)} \tag{114}$$

We know by the induction step that,

$$\frac{p_{ss_1}(t)}{p_{ss_2}(t) + p_{ss_1}(t)} \leq r \ . \tag{115}$$

Also note that,

$$\frac{f_{\overleftarrow{ss_1}}(t)}{f_{\overleftarrow{ss_2}}(t) + f_{\overleftarrow{ss_1}}(t)} = \frac{\frac{f_{\overleftarrow{ss_1}}(t)}{f_{\overleftarrow{ss_2}}(t)}}{1 + \frac{f_{\overleftarrow{ss_1}}(t)}{f_{\overleftarrow{ss_2}}(t)}} \leq \frac{\frac{r \cdot f_{\overleftarrow{d}}(t-n)}{(1-r) \cdot f_{\overleftarrow{d}}(t-m)}}{1 + \frac{r \cdot f_{\overleftarrow{d}}(t-n)}{(1-r) \cdot f_{\overleftarrow{d}}(t-m)}} = \frac{r \cdot f_{\overleftarrow{d}}(t-n)}{f_{\overleftarrow{d}}(t-m) + r \cdot (f_{\overleftarrow{d}}(t-n) - f_{\overleftarrow{d}}(t-m))} \leq \frac{f_{\overleftarrow{d}}(t-n)}{f_{\overleftarrow{d}}(t-m)} \cdot r \ . \tag{116}$$

In the above we used the monotonically non-decreasing property of $x/(1+x)$ and the conditions provided by the induction step at time t, that is $f_{\overleftarrow{ss_1}}(t) \leq r \cdot f_{\overleftarrow{d}}(t-n)$ and $f_{\overleftarrow{ss_2}}(t) \geq (1-r) \cdot f_{\overleftarrow{d}}(t-m)$; which implies that $\frac{f_{\overleftarrow{ss_1}}(t)}{f_{\overleftarrow{ss_2}}(t)} \leq \frac{r \cdot f_{\overleftarrow{d}}(t-n)}{(1-r) \cdot f_{\overleftarrow{d}}(t-m)}$. In the final inequality, we used $0 \leq r \leq 1$ and $f_{\overleftarrow{d}}(t-n) - f_{\overleftarrow{d}}(t-m) \geq 0$. Note that $f_{\overleftarrow{d}}(t-n) - f_{\overleftarrow{d}}(t-m) \geq 0$ follows because $n \leq m$ and the fact that the incoming flow is non-decreasing.

Let $c_{g,r} \stackrel{\text{def}}{=} r - g(r) > 0$, then we have following upper bound on the flow value,

$$f_{\overrightarrow{ss_1}}(t) = f_{\overrightarrow{s}}(t) \cdot g(\overline{p}_{\overrightarrow{ss_1}}(t)) \leq f_{\overrightarrow{s}}(t) \cdot g(r) = f_{\overrightarrow{s}}(t) \cdot (r - c_{g,r}) \ , \tag{117}$$

In the above we used $\overline{p}_{\overrightarrow{ss_1}}(t) \leq r$ and monotonicity of decision rule g. Using these bounds, we provide an

upper bound for the normalized pheromone level.

$$\overline{p}_{\overrightarrow{ss_1}}(t+1) = \frac{p_{ss_1}(t) + f_{\overrightarrow{ss_1}}(t) + f_{\overleftarrow{ss_1}}(t)}{p_{ss_2}(t) + p_{ss_1}(t) + f_{\overrightarrow{ss_2}}(t) + f_{\overrightarrow{ss_1}}(t) + f_{\overleftarrow{ss_2}}(t) + f_{\overleftarrow{ss_1}}(t)}, \tag{118}$$

$$\leq \frac{r \cdot (p_{ss_2}(t) + p_{ss_1}(t)) + f_{\overrightarrow{ss_1}}(t) + \frac{f_{\overleftarrow{d}}(t-n)}{f_{\overleftarrow{d}}(t-m)} \cdot r \cdot (f_{\overleftarrow{ss_2}}(t) + f_{\overleftarrow{ss_1}}(t))}{p_{ss_2}(t) + p_{ss_1}(t) + f_{\overrightarrow{ss_2}}(t) + f_{\overrightarrow{ss_1}}(t) + f_{\overleftarrow{ss_2}}(t) + f_{\overleftarrow{ss_1}}(t)}, \tag{119}$$

$$= r + \frac{(\frac{f_{\overleftarrow{d}}(t-n)}{f_{\overleftarrow{d}}(t-m)} - 1) \cdot r \cdot (f_{\overleftarrow{ss_2}}(t) + f_{\overleftarrow{ss_1}}(t)) + \Big(f_{\overrightarrow{ss_1}}(t) - r(f_{\overrightarrow{ss_1}}(t) + f_{\overrightarrow{ss_2}}(t))\Big)}{p_{ss_2}(t) + p_{ss_1}(t) + f_{\overrightarrow{ss_2}}(t) + f_{\overrightarrow{ss_1}}(t) + f_{\overleftarrow{ss_2}}(t) + f_{\overleftarrow{ss_1}}(t)}, \tag{120}$$

$$\leq r + \frac{(\frac{f_{\overleftarrow{d}}(t-n)}{f_{\overleftarrow{d}}(t-m)} - 1) \cdot r \cdot (f_{\overleftarrow{d}}(t-m) + f_{\overleftarrow{d}}(t-n)) + f_{\overrightarrow{s}}(t)\left(r - c_{g,r} - r\right)}{p_{ss_2}(t) + p_{ss_1}(t) + f_{\overrightarrow{ss_2}}(t) + f_{\overrightarrow{ss_1}}(t) + f_{\overleftarrow{ss_2}}(t) + f_{\overleftarrow{ss_1}}(t)}, \tag{121}$$

$$\leq r + \frac{(\frac{f_{\overleftarrow{d}}(t-n)}{f_{\overleftarrow{d}}(t-m)} - 1) \cdot r \cdot (f_{\overleftarrow{d}}(t-m) + f_{\overleftarrow{d}}(t-n)) - c_{g,r} f_{\overrightarrow{s}}(t)}{p_{ss_2}(t) + p_{ss_1}(t) + f_{\overrightarrow{ss_2}}(t) + f_{\overrightarrow{ss_1}}(t) + f_{\overleftarrow{ss_2}}(t) + f_{\overleftarrow{ss_1}}(t)}, \tag{122}$$

$$\leq r - \frac{\frac{1}{2} c_{g,r} f_{\overrightarrow{s}}(t)}{p_{ss_2}(t) + p_{ss_1}(t) + f_{\overrightarrow{ss_2}}(t) + f_{\overrightarrow{ss_1}}(t) + f_{\overleftarrow{ss_2}}(t) + f_{\overleftarrow{ss_1}}(t)}, \tag{123}$$

$$\leq r\,. \tag{124}$$

In the second inequality we used Equations (115) and (116). In the third equality, we rearranged terms. In the fourth inequality, we used the following inequalities: $\frac{f_{\overleftarrow{d}}(t-n)}{f_{\overleftarrow{d}}(t-m)} - 1 \geq 0$, $f_{\overleftarrow{ss_2}}(t) \leq f_{\overleftarrow{d}}(t-m)$, $f_{\overleftarrow{ss_1}}(t) \leq f_{\overleftarrow{d}}(t-n)$, $f_{\overrightarrow{ss_1}}(t) + f_{\overrightarrow{ss_2}}(t) \leq f_{\overrightarrow{s}}(t)$ and Equation (117). In the fifth inequality, we simplified the expression. The sixth inequality holds if $(\frac{f_{\overleftarrow{d}}(t-n)}{f_{\overleftarrow{d}}(t-m)} - 1) \cdot r \cdot (f_{\overleftarrow{d}}(t-m) + f_{\overleftarrow{d}}(t-n)) \leq \frac{1}{2} c_{g,r} f_{\overrightarrow{s}}(t)$.

We will set functions $f_{\overrightarrow{s}}(t)$ and $f_{\overleftarrow{d}}(t)$ such that the this condition is satisfied. Suppose we set $f_{\overrightarrow{s}}(t) = f_{\overleftarrow{d}}(t) = (1+\alpha)^t$ for some $\alpha \geq 0$. Then the condition above is satisfied if α satisfies

$$(1+\alpha)^{m-n} \leq 1 + \frac{c_{g,r}}{2 \cdot r} \frac{1}{(1+\alpha)^{-n} + (1+\alpha)^{-m}} = 1 + \frac{c_{g,r}}{2 \cdot r} \frac{(1+\alpha)^m}{1 + (1+\alpha)^{m-n}} \tag{125}$$

Suppose we set α small enough such that $(1+\alpha)^{m-n} < 1 + \frac{1}{3}\min(\frac{c_{g,r}}{2 \cdot r}, 1)$, then α satisfies

$$(1+\alpha)^{m-n} \leq 1 + \frac{c_{g,r}}{2 \cdot r} \cdot \frac{1}{3} \leq 1 + \frac{c_{g,r}}{2 \cdot r} \cdot \frac{1}{1 + (1+\alpha)^{m-n}} \leq 1 + \frac{c_{g,r}}{2 \cdot r} \cdot \frac{(1+\alpha)^m}{1 + (1+\alpha)^{m-n}}\,. \tag{126}$$

In the first inequality we used $(1+\alpha)^{m-n} < 1 + \frac{1}{3} \cdot \frac{c_{g,r}}{2 \cdot r}$. In the second inequality we used $(1+\alpha)^{m-n} < 1 + \frac{1}{3} \leq 2$. In the third inequality we used $\alpha \geq 0$, which implies $(1+\alpha)^m \geq 1$. Therefore there exists a setting of α which depends only on g, n and m such that when the incoming flow increases by a factor of $1+\alpha$ at each time step, the previous analysis goes through and we get

$$\overline{p}_{\overrightarrow{ss_1}}(t+1) \leq r\,.$$

Therefore, by induction, the dynamics never converges to the shortest path P_1. We conclude the first case.

Case 2: Suppose $g(r) > r$, then we pick the following initial configuration:

- Assign pheromone value on the edges $(s, s_1), (s, s_2), (d_1, d), (d_2, d)$ such that the normalized pheromone level on $(s, s_2), (d_2, d)$ is $\geq r$, that is $\overline{p}_{\overrightarrow{ss_2}}(0), \overline{p}_{\overleftarrow{d_2d}}(0) \geq r$. Further assign the flow values on the edges such that they satisfy the following,

$$f_{\overrightarrow{v_iv_{i+1}}}(0) \leq f_{\vec{s}}(0) \cdot (1-r) \text{ and } f_{\overleftarrow{v_iv_{i+1}}}(0) \leq f_{\overleftarrow{d}}(0) \cdot (1-r) \tag{127}$$

$$f_{\overrightarrow{u_iu_{i+1}}}(0) \geq f_{\vec{s}}(0) \cdot r \text{ and } f_{\overleftarrow{v_iv_{i+1}}}(0) \geq f_{\overleftarrow{d}}(0) \cdot r\ . \tag{128}$$

- Let $f_{\vec{s}}(0) = f_{\overleftarrow{d}}(0) = 1$. We specify the values of flow $f_{\vec{s}}(t), f_{\overleftarrow{d}}(t)$ for times $t > 0$ later in the proof.

We show by induction that the above inequalities on flow and pheromone levels hold for all time $t \geq 0$ and therefore the system does not converge to the shortest path.

Hypothesis: At time $t \geq 0$, pheromone value on the edges $(s, s_1), (s, s_2), (d_1, d), (d_2, d)$ are such that the normalized pheromone level on $(s, s_2), (d_2, d)$ is $\geq r$, that is $\overline{p}_{\overrightarrow{ss_2}}(t), \overline{p}_{\overleftarrow{d_2d}}(t) \geq r$. Further the flow values on the edges satisfy the following,

$$f_{\overrightarrow{v_iv_{i+1}}}(t) \leq f_{\vec{s}}(t-i) \cdot (1-r) \text{ and } f_{\overleftarrow{v_iv_{i+1}}}(t) \leq f_{\overleftarrow{d}}(t-(n-i)) \cdot (1-r) \tag{129}$$

$$f_{\overrightarrow{u_iu_{i+1}}}(t) \geq f_{\vec{s}}(t-i) \cdot r \text{ and } f_{\overleftarrow{v_iv_{i+1}}}(t) \geq f_{\overleftarrow{d}}(t-(m-i)) \cdot r\ . \tag{130}$$

In the above equations, $f_{\vec{s}}(t') = f_{\vec{s}}(0)$ and $f_{\overleftarrow{d}}(t') = f_{\overleftarrow{d}}(0)$ for all $t' < 0$.

Base case: The conditions trivially hold at time 0 because of the initial setting of flow and pheromone levels described above.

Induction Step: To prove the hypothesis for time $t+1$, all we need to show is that, $\overline{p}_{\overrightarrow{ss_2}}(t+1), \overline{p}_{\overleftarrow{d_2d}}(t+1) \geq r$, $f_{\overrightarrow{u_0u_1}}(t+1) = f_{\overrightarrow{ss_2}}(t+1) \geq f_{\vec{s}}(t+1) \cdot r$ and $f_{\overleftarrow{u_mu_{m+1}}}(t+1) = f_{\overleftarrow{d_2d}}(t+1) \geq f_{\overleftarrow{d}}(t+1) \cdot r$; all the remaining inequalities follows from these basic inequalities. Also note that from the definition of our case, that is $g(r) > r$, we get that the inequalities $\overline{p}_{\overrightarrow{ss_2}}(t+1), \overline{p}_{\overleftarrow{d_2d}}(t+1) \geq r$ further imply $f_{\overrightarrow{ss_2}}(t+1) \geq f_{\vec{s}}(t+1) \cdot r$ and $f_{\overleftarrow{d_2d}}(t+1) \geq f_{\overleftarrow{d}}(t+1) \cdot r$.

To see this, note that

$$f_{\overrightarrow{ss_2}}(t+1) = f_{\vec{s}}(t+1) \cdot g(\overline{p}_{\overrightarrow{ss_2}}(t+1)) \geq f_{\vec{s}}(t+1) \cdot g(r) \geq f_{\vec{s}}(t+1) \cdot r\ ,$$

when $\overline{p}_{\overrightarrow{ss_2}}(t+1) \leq 1/2$ and,

$$f_{\overrightarrow{ss_2}}(t+1) = f_{\vec{s}}(t+1) \cdot (1 - g(1 - \overline{p}_{\overrightarrow{ss_2}}(t+1))) \geq f_{\vec{s}}(t+1) \cdot (1 - g(1/2)) = f_{\vec{s}}(t+1) \cdot \frac{1}{2} \geq f_{\vec{s}}(t+1) \cdot r\ ,$$

when $\overline{p}_{\overrightarrow{ss_2}}(t+1) \geq 1/2$, where we used monotonically non-decreasing property of g for the above inequalities. Also, we used $g(1/2) = 1/2$ and $r \leq 1/2$ for the last inequality. Here, we had to consider two cases because function g takes as input the minimum of the two normalized pheromone levels. Similarly we can show $f_{\overleftarrow{d_2d}}(t+1) \geq f_{\overleftarrow{d}}(t+1) \cdot r$.

Therefore it is enough to show that $\overline{p}_{\overrightarrow{ss_2}}(t+1), \overline{p}_{\overleftarrow{d_2d}}(t+1) \geq r$. As the proof for the bound on $\overline{p}_{\overleftarrow{d_2d}}(t+1)$ is analogous to that of $\overline{p}_{\overrightarrow{ss_2}}(t+1)$, in the remainder we focus our attention on the proof for $\overline{p}_{\overrightarrow{ss_2}}(t+1)$. Recall the definition of $\overline{p}_{\overrightarrow{ss_2}}(t+1)$,

$$\overline{p}_{\overrightarrow{ss_2}}(t+1) = \frac{p_{ss_2}(t+1)}{p_{ss_1}(t+1) + p_{ss_2}(t+1)} = \frac{p_{ss_2}(t) + f_{\overrightarrow{ss_2}}(t) + f_{\overleftarrow{ss_2}}(t)}{p_{ss_1}(t) + p_{ss_2}(t) + f_{\overrightarrow{ss_1}}(t) + f_{\overrightarrow{ss_2}}(t) + f_{\overleftarrow{ss_1}}(t) + f_{\overleftarrow{ss_2}}(t)} \tag{131}$$

We know by the induction step that,

$$\frac{p_{ss_2}(t)}{p_{ss_1}(t) + p_{ss_2}(t)} \geq r \ . \tag{132}$$

Also note that,

$$\frac{f_{\overleftarrow{ss_2}}(t)}{f_{\overleftarrow{ss_1}}(t) + f_{\overleftarrow{ss_2}}(t)} = \frac{\frac{f_{\overleftarrow{ss_2}}(t)}{f_{\overleftarrow{ss_1}}(t)}}{1 + \frac{f_{\overleftarrow{ss_2}}(t)}{f_{\overleftarrow{ss_1}}(t)}} \geq \frac{\frac{r \cdot f_{\overleftarrow{d}}(t-m)}{(1-r)\cdot f_{\overleftarrow{d}}(t-n)}}{1 + \frac{r \cdot f_{\overleftarrow{d}}(t-m)}{(1-r)\cdot f_{\overleftarrow{d}}(t-n)}} = \frac{r \cdot f_{\overleftarrow{d}}(t-m)}{f_{\overleftarrow{d}}(t-n) - r \cdot (f_{\overleftarrow{d}}(t-n) - f_{\overleftarrow{d}}(t-m))} \geq \frac{f_{\overleftarrow{d}}(t-m)}{f_{\overleftarrow{d}}(t-n)} \cdot r \ . \tag{133}$$

In the above we used the monotonically non-decreasing property of $x/(1+x)$ and the conditions provided by the induction step at time t, that is $f_{\overleftarrow{ss_2}}(t) \geq r \cdot f_{\overleftarrow{d}}(t-m)$ and $f_{\overleftarrow{ss_1}}(t) \leq (1-r) \cdot f_{\overleftarrow{d}}(t-n)$; which implies $\frac{f_{\overleftarrow{ss_2}}(t)}{f_{\overleftarrow{ss_1}}(t)} \geq \frac{r \cdot f_{\overleftarrow{d}}(t-m)}{(1-r)\cdot f_{\overleftarrow{d}}(t-n)}$. In the final inequality we used $0 \leq r \leq 1$ and $f_{\overleftarrow{d}}(t-n) - f_{\overleftarrow{d}}(t-m)) \geq 0$, which follows because $n \leq m$ and the incoming flow is non-decreasing.

Let $c_{g,r} \stackrel{\text{def}}{=} g(r) - r > 0$. We have the following upper bound on the flow value,

$$f_{\overrightarrow{ss_2}}(t) \geq f_{\overrightarrow{s}}(t) \cdot (r + c_{g,r}) \ , \tag{134}$$

To see this, note that

$$f_{\overrightarrow{ss_2}}(t) = f_{\overrightarrow{s}}(t) \cdot g(\overline{p}_{\overrightarrow{ss_2}}(t)) \geq f_{\overrightarrow{s}}(t) \cdot g(r) = f_{\overrightarrow{s}}(t) \cdot (r + c_{g,r}) \ ,$$

when $\overline{p}_{\overrightarrow{ss_2}}(t) \leq 1/2$. In the above we used $\overline{p}_{\overrightarrow{ss_2}}(t) \geq r$ and monotonicity of decision rule g. And

$$\begin{aligned} f_{\overrightarrow{ss_2}}(t) &= f_{\overrightarrow{s}}(t) \cdot (1 - g(1 - \overline{p}_{\overrightarrow{ss_2}}(t))) \geq f_{\overrightarrow{s}}(t) \cdot (1 - g(1/2)) \\ &= f_{\overrightarrow{s}}(t) \cdot \frac{1}{2} = f_{\overrightarrow{s}}(t) \cdot g(1/2) \geq f_{\overrightarrow{s}}(t) \cdot g(r) = f_{\overrightarrow{s}}(t) \cdot (r + c_{g,r}) \ , \end{aligned}$$

when $\overline{p}_{\overrightarrow{ss_2}}(t) > 1/2$. In the above, we used $\overline{p}_{\overrightarrow{ss_2}}(t) > 1/2$, monotonicity of decision rule g, $g(1/2) = 1/2$ and $r \leq 1/2$.

Using these bounds, we provide an upper bound for the normalized pheromone level.

$$\overline{p}_{\overrightarrow{ss_2}}(t+1) = \frac{p_{ss_2}(t) + f_{\overrightarrow{ss_2}}(t) + f_{\overleftarrow{ss_2}}(t)}{p_{ss_1}(t) + p_{ss_2}(t) + f_{\overrightarrow{ss_1}}(t) + f_{\overrightarrow{ss_2}}(t) + f_{\overleftarrow{ss_1}}(t) + f_{\overleftarrow{ss_2}}(t)}, \tag{135}$$

$$\geq \frac{r \cdot (p_{ss_1}(t) + p_{ss_2}(t)) + f_{\overrightarrow{ss_2}}(t) + \frac{f_{\overleftarrow{d}}(t-m)}{f_{\overleftarrow{d}}(t-n)} \cdot r \cdot (f_{\overleftarrow{ss_2}}(t) + f_{\overleftarrow{ss_1}}(t))}{p_{ss_1}(t) + p_{ss_2}(t) + f_{\overrightarrow{ss_1}}(t) + f_{\overrightarrow{ss_2}}(t) + f_{\overleftarrow{ss_1}}(t) + f_{\overleftarrow{ss_2}}(t)}, \tag{136}$$

$$= r + \frac{(\frac{f_{\overleftarrow{d}}(t-m)}{f_{\overleftarrow{d}}(t-n)} - 1) \cdot r \cdot (f_{\overleftarrow{ss_2}}(t) + f_{\overleftarrow{ss_1}}(t)) + \left(f_{\overrightarrow{ss_2}}(t) - r \cdot (f_{\overrightarrow{ss_1}}(t) + f_{\overrightarrow{ss_2}}(t))\right)}{p_{ss_1}(t) + p_{ss_2}(t) + f_{\overrightarrow{ss_1}}(t) + f_{\overrightarrow{ss_2}}(t) + f_{\overleftarrow{ss_1}}(t) + f_{\overleftarrow{ss_2}}(t)}, \tag{137}$$

$$\geq r + \frac{(\frac{f_{\overleftarrow{d}}(t-m)}{f_{\overleftarrow{d}}(t-n)} - 1) \cdot r \cdot (f_{\overleftarrow{d}}(t-m) + f_{\overleftarrow{d}}(t-n)) + f_{\overrightarrow{s}}(t) \cdot \left(r + c_{g,r} - r\right)}{p_{ss_1}(t) + p_{ss_2}(t) + f_{\overrightarrow{ss_1}}(t) + f_{\overrightarrow{ss_2}}(t) + f_{\overleftarrow{ss_1}}(t) + f_{\overleftarrow{ss_2}}(t)}, \tag{138}$$

$$\geq r + \frac{(\frac{f_{\overleftarrow{d}}(t-m)}{f_{\overleftarrow{d}}(t-n)} - 1) \cdot r \cdot (f_{\overleftarrow{d}}(t-m) + f_{\overleftarrow{d}}(t-n)) + c_{g,r} f_{\overrightarrow{s}}(t)}{p_{ss_1}(t) + p_{ss_2}(t) + f_{\overrightarrow{ss_1}}(t) + f_{\overrightarrow{ss_2}}(t) + f_{\overleftarrow{ss_1}}(t) + f_{\overleftarrow{ss_2}}(t)}, \tag{139}$$

$$\geq r + \frac{\frac{1}{2} c_{g,r} f_{\overrightarrow{s}}(t)}{p_{ss_1}(t) + p_{ss_2}(t) + f_{\overrightarrow{ss_1}}(t) + f_{\overrightarrow{ss_2}}(t) + f_{\overleftarrow{ss_1}}(t) + f_{\overleftarrow{ss_2}}(t)}, \tag{140}$$

$$\geq r\,. \tag{141}$$

In the second inequality we used Equations (132) and (133). In the third equality, we rearranged terms. In the fourth inequality, we used the following inequalities: $\frac{f_{\overleftarrow{d}}(t-m)}{f_{\overleftarrow{d}}(t-n)} - 1 \leq 0$, $f_{\overleftarrow{ss_2}}(t) \leq f_{\overleftarrow{d}}(t-m)$, $f_{\overleftarrow{ss_1}}(t) \leq f_{\overleftarrow{d}}(t-n)$, $f_{\overrightarrow{ss_1}}(t) + f_{\overrightarrow{ss_2}}(t) \leq f_{\overrightarrow{s}}(t)$ and Equation (134). In the fifth inequality, we simplified the expression. The sixth inequality holds if $(\frac{f_{\overleftarrow{d}}(t-m)}{f_{\overleftarrow{d}}(t-n)} - 1) \cdot r \cdot (f_{\overleftarrow{d}}(t-m) + f_{\overleftarrow{d}}(t-n)) \geq -\frac{1}{2} c_{g,r} f_{\overrightarrow{s}}(t)$.

We will set functions $f_{\overrightarrow{s}}(t)$ and $f_{\overleftarrow{d}}(t)$ such that the this condition is satisfied. Suppose we set $f_{\overrightarrow{s}}(t) = f_{\overleftarrow{d}}(t) = (1+\alpha)^t$ for some $\alpha \geq 0$. Then the condition above is satisfied if α satisfies

$$(1+\alpha)^{n-m} \geq 1 - \frac{c_{g,r}}{2 \cdot r} \frac{1}{(1+\alpha)^{-m} + (1+\alpha)^{-n}} = 1 - \frac{c_{g,r}}{2 \cdot r} \frac{(1+\alpha)^n}{(1+\alpha)^{n-m} + 1}. \tag{142}$$

Recall that $n \leq m$. Suppose we set α small enough such that $(1+\alpha)^{n-m} \geq 1 - \frac{1}{2} \min(\frac{c_{g,r}}{2 \cdot r}, 1)$, then note that,

$$(1+\alpha)^{n-m} \geq 1 - \frac{1}{2} \cdot \frac{c_{g,r}}{2 \cdot r} \geq 1 - \frac{c_{g,r}}{2 \cdot r} \cdot \frac{1}{1 + (1+\alpha)^{n-m}} \geq 1 - \frac{c_{g,r}}{2 \cdot r} \cdot \frac{(1+\alpha)^n}{1 + (1+\alpha)^{n-m}} \tag{143}$$

In the first inequality, we used $(1+\alpha)^{n-m} \geq 1 - \frac{1}{2} \min(\frac{c_{g,r}}{2 \cdot r}, 1) \geq 1 - \frac{1}{2} \frac{c_{g,r}}{2 \cdot r}$. In the second inequality we used, $\alpha \geq 0$, $n \leq m$; which implies $(1+\alpha)^{n-m} \leq 1$. In the final inequality, we used $\alpha \geq 0$, which implies $(1+\alpha)^n \geq 1$. Therefore there exists a setting of α which depends only on g, n and m such that when the incoming flow increases by a factor of $1+\alpha$ at each time step, the previous analysis goes through and we get

$$\overline{p}_{\overrightarrow{ss_2}}(t+1) \geq r\,.$$

Therefore, by induction, the dynamics never converges to the shortest path P_1. We conclude the second case. □

C Simulation Details

In Section 3, we discussed various simulation results for linear and non-linear decision rules. We give more details of these simulations here.

Graph families considered. We ran the simulations for three kinds of directed graphs: graphs sampled from the $G(n, p)$ model, graphs sampled from the $G(n, p)$ model with the additional locality constraint that an edge can exist between vertex i and j only if $|i - j| \leq k$ for some parameter k (with the source vertex and the destination the vertex numbered 1 and n respectively), and the grid graph. $G(n, p)$ model with the additional constraint ensures that the graph has long paths between the source and the destination. We only considered instances where there was at least one path from the source to the destination.

For the $G(n, p)$ model and its locally constrained version, we consider two kinds of graph families: one where an edge is allowed from i to j only if $i < j$ (resulting in a DAG), and the other where the edges can go both ways. We will use $G(n, p)$ and $G(n, p)$ local to denote the $G(n, p)$ graph and it's locally constrained version where edges can go both ways, and $G(n, p)$ DAG and $G(n, p)$ local DAG to denote their acyclic versions respectively.

C.1 Linear Decision Rule

In Section 3.1, we discussed that with linear decision rule, in the fixed incoming flow setting, the dynamics converges to the path with the minimum leakage. With increasing flow, and no leakage, the dynamics converges to the shortest path. We generated a large number of instances with different values for n, p, k and other parameters and observed the desired convergence in all the simulated instances. Below, we describe the details of the parameter settings we considered.

For all the simulations, the decay parameter δ was set to 0.9. The initial forward flow level at s and backward flow level at d was chosen uniformly at random from $(0.5, 1)$, and the initial flow at all other vertices was set to 0. We ran each simulation instance with two settings for initial pheromone level: (i) setting initial pheromone levels at all edges to 1, (ii) choosing initial pheromone level on the edges uniformly at random from $(0, 1)$.

C.1.1 Leakage with fixed flow

For all the randomly generated instance, we chose the leakage value of all the vertices uniformly at random from $(0, 0.1)$. For all these instances, the dynamics converged to the path with the minimum leakage. Below, we describe the parameter values for different graph families.

1. $G(n, p)$ and $G(n, p)$ DAG: We ran 1000 instances each with (n, p) set to $(100, 0.05)$, $(100, 0.1)$ and $(100, 0.5)$. We ran 100 instances each with (n, p) set to $(1000, 0.01)$ and $(1000, 0.005)$.
2. $G(n, p)$ local and $G(n, p)$ local DAG: We ran 1000 instances with $(n, p) = (100, 0.5)$ and window size of 10, and 10 instances with $(n, p) = (1000, 0.5)$ and window size of 40.
3. Grid graph: We ran the simulations on a $10X10$ grid graph with source and destination at the diagonally opposite extreme vertices. We ran 100 instances. The graph structure was same across all these instances, but other parameter values such as leakage levels, initial pheromone levels etc. were chosen randomly as described above.

C.1.2 Increasing flow with no leakage

In this case, the leakage was set to 0 for all the vertices. We increase the forward flow level at s and backward flow level at d by a factor of 1.1 every time step. To avoid floating point overflow, instead of multiplying the forward and backward flow values at s and d by 1.1, we keep these two values the same,

and divide all other flow values and pheromone levels in the graph by a factor of 1.1. For any decision rule that only depends on the normalized pheromone levels (which holds for the linear rule), it is not difficult to see that only the relative values of flow and pheromone level matter, and this gives rise to exactly the same dynamics (up to scaling) as when we multiply the forward flow at s and backward flow at d by 1.1. When the value of pheromone level or flow at any edge became too small (smaller than the minimum allowed value for a 64 bit floating point number which is $\approx 10^{-323}$), we rounded it to zero.

For all the graphs, we planted a short path in the graph so as to ensure that the shortest path is unique. In all the simulations, we observed that the dynamics converges to this shortest path. Below, we describe the details for different graph types.

1. $G(n,p)$ and $G(n,p)$ DAG: We ran 1000 instances each with (n,p) set to $(100,0.05),(100,0.1)$ and $(100,0.5)$. We ran 100 instances each with (n,p) set to $(1000,0.01)$ and $(1000,0.005)$. For all the instances, we plant a randomly generated shortest path of length 1 smaller than the previous shortest path, so that there is unique shortest path in the graph so obtained.
2. $G(n,p)$ local and $G(n,p)$ local DAG: We ran 1000 instances with $(n,p)=(100,0.5)$ and window size of 10, and 100 instances with $(n,p)=(1000,0.5)$ and window size of 40. We planted the shortest path having edges $(1,k+r),(k+r,2(k+r)),(2(k+r),3(k+r))$ and so on. Here, 1 is the source vertex and n is the destination vertex, and r is a random integer in $[1,\text{window size}]$.
3. Grid graph: We ran the simulations on a $10X10$ grid graph with source and destination at the diagonally opposite extreme vertices. We ran 100 instances. For each instance, we planted a randomly generated shortest path of length randomly chosen from $\{9,10,11\}$.

C.2 Nonlinear Decision Rules

In Section 3.2, we showed that for a reasonably general family of decision rules, the linear decision rule is its unique member with guaranteed convergence to the path with minimum leakage or the shortest path. Note that these results only suggest that linear decision rule is necessary for *guaranteed convergence* to the shortest or the minimum leakage path. Can it be the case that even with non-linear decision rules, the forces of increasing flow and leakage still help in finding shorter or smaller leakage paths respectively, compared to the paths found in the absence of these forces? To understand this question, we ran simulations for various non-linear decision rules. We observe that within each graph family, for a large fraction of graph instances, the path found in the presence of these forces has length (respectively leakage) smaller than or equal to the length (respectively leakage) of the path found in their absence. These simulations suggest that the usefulness of the forces of leakage and increasing flow is not limited to the linear decision rule. Below, we discuss more details of these simulations.

C.2.1 Decision rules details

We consider the following non-linear decision rules:

- **Quadratic**: This rule divides the flow in proportion to the square of the pheromone levels.
- **1.1 power**: This rule divides the flow in proportion to the 1.1^{th} power of the pheromone levels. We study this rule to understand the effect of strength of non-linearity. Since this rule is closer to the linear rule compared to the quadratic rule, we would expect that the forces of leakage and increasing flow are more effective with this rule compared to the quadratic rule.
- **Quadratic-with-offset**: This rule is a slight variant of the quadratic rule and has been used previously [1] to model ant behaviour. This rule adds a fixed positive constant c to the pheromone levels, and divides the flow in proportion to the square of the offsetted pheromone levels. The parameter c was added to encourage exploration.

- **Rank-edge**: This rule was introduced in [2]. This rule ranks the edges from highest to lowest pheromone levels. If multiple edges have the same pheromone level, they get the same rank. Let $q \in (0,1)$ be some parameter. This rule sends $(1-q)$ fraction of flow to the first ranked edge, $q(1-q)$ fraction of flow to the second ranked edge, $q^2(1-q)$ fraction of flow to the third ranked edge and so on. In general, the ith ranked edge gets $q^{i-1}(1-q)$ fraction of flow. If there are k edges at the ith rank, then each of them gets $\frac{q^{i-1}(1-q)}{k}$ fraction of flow. One exception to this rule is the lowest ranked edge which gets all the remaining flow that has not been assigned to any other edge. If there are multiple edges at the lowest rank, then this remaining flow gets divided equally among them.

C.2.2 Results

As in the linear decision rule case, we considered the $G(n,p)$, $G(n,p)$ local, $G(n,p)$ DAG, $G(n,p)$ local DAG and the grid graph for our simulations with non-linear decision rules. In the increasing flow case, similar to the linear decision rule setting, we add a random shortest path to these graphs so that the shortest path is unique. For each non-linear decision rule and each graph family, we ran simulations with at least 100 random graph instances.

In Table 1, we show the effect of leakage with non-linear decision rules. There is a subtle distinction here between path leakage as an objective function and leakage as a process affecting the dynamics. For each graph instance, we assign leakage values to vertices. This gives us a path leakage objective function for each graph instance. For each graph instance, we compare two settings, the baseline setting in which the leakage process is not applied at vertices during the dynamics and the main setting in which the leakage process is applied. Now for each graph instance, we compare the path leakage objective function of the path found in the baseline setting and the main setting. We report the fraction of instances where the path leakage objective in the main setting is unchanged or smaller as compared to the baseline setting. We also report the average percentage difference in the path leakage objective between the baseline and the main setting, that is

$$\frac{1}{n}\sum_{i=1}^{n} 100 * \frac{\text{path\_leakage}(\text{main}_i) - \text{path\_leakage}(\text{baseline}_i)}{\text{path\_leakage}(\text{baseline}_i)}.$$

Here, $\text{path\_leakage}(\text{main}_i)$ and $\text{path\_leakage}(\text{baseline}_i)$ denote the path leakage objective obtained in the main setting and the baseline setting respectively, for the ith graph instance. In the last column, we report the average percentage difference in path leakage for the baseline compared to the optimum (minimum) path leakage, that is

$$\frac{1}{n}\sum_{i=1}^{n} 100 * \frac{\text{path\_leakage}(\text{opt}_i) - \text{path\_leakage}(\text{baseline}_i)}{\text{path\_leakage}(\text{baseline}_i)}.$$

Here, $\text{path\_leakage}(\text{opt}_i)$ denotes the optimum path leakage for the ith graph instance. Due to symmetry in the grid graph, the dynamics does not converge to a path in the baseline setting, therefore we do not consider grid graphs while studying the effect of leakage with non-linear decision rules.

In Table 2, we show the effect of increasing flow with non-linear decision rules. Again, we consider the baseline setting where there is no increasing flow, and the main setting with increasing flow. We report the fraction of instances where the path length in the main setting is unchanged or smaller as compared to the baseline setting. We also report the average percentage difference in the length of path found between the baseline and the main setting, that is

$$\frac{1}{n}\sum_{i=1}^{n} 100 * \frac{\text{path\_length}(\text{main}_i) - \text{path\_length}(\text{baseline}_i)}{\text{path\_length}(\text{baseline}_i)}.$$

Here, $\text{path\_length}(\text{main}_i)$ and $\text{path\_length}(\text{baseline}_i)$ denote the path length obtained in the main setting and the baseline setting respectively, for the ith graph instance. In the last column, we report the average

Decision rule	Graph family	% instances with unchanged path leak.	% instances with smaller path leak.	Avg. % change	Avg. % change opt.
1.1 power	$G(n,p)$ local	1	98	-27.5	-43.9
	$G(n,p)$ local DAG	0	100	-38.1	-47.7
	$G(n,p)$	70	30	-12.3	-26.1
	$G(n,p)$ DAG	88	12	-3.9	-17.7
Quadratic	$G(n,p)$ local	27	67	-8.2	-44.3
	$G(n,p)$ local DAG	24	73	-12.5	-47.2
	$G(n,p)$	83	16	-4.5	-24.8
	$G(n,p)$ DAG	94	6	-2.4	-22.3
Quadratic -with- offset	$G(n,p)$ local	2	79	-10.5	-43.1
	$G(n,p)$ local DAG	1	93	-17.6	-47.6
	$G(n,p)$	84	14	-5.2	-25.4
	$G(n,p)$ DAG	93	6	-2.7	-22.6
Rank-edge	$G(n,p)$ local	67	32	-2.8	-40.6
	$G(n,p)$ local DAG	63	31	-2.5	-40.2
	$G(n,p)$	78	22	-4.1	-21.2
	$G(n,p)$ DAG	81	19	-4.1	-15.2

Table 1: Effect of leakage with non-linear decision rules.

percentage difference in path length for the baseline compared to the shortest length path, that is

$$\frac{1}{n}\sum_{i=1}^{n} 100 * \frac{\text{path\_length}(\text{opt}_i) - \text{path\_length}(\text{baseline}_i)}{\text{path\_length}(\text{baseline}_i)}.$$

Here, $\text{path\_length}(\text{opt}_i)$ denotes the shortest path length for the ith graph instance.

We discuss our major observations below:

- **Usefulness of forces of leakage and increasing flow not limited to the linear rule**: For a large fraction of graph instances in each graph family, the path found in the presence of these forces has length (respectively leakage) smaller than or equal to the length (respectively leakage) of the path found in their absence. This holds for all non-linear decision rules considered.
- **Forces of leakage and increasing flow more effective with weaker non-linearity**: One would intuitively hope that the closer a non-linear decision rule is to the linear rule, the more effective the forces of leakage and increasing flow would be. To study this, we compare the quadratic decision rule with 1.1 power rule. For all graph families, we observe that the percentage of instances with strictly smaller path leakage (path length resp.) is higher for 1.1 power rule. We also observe that the average percentage change in path leakage (path length resp.) is more negative for 1.1 power rule. This shows that the forces of leakage and increasing flow are more effective for 1.1 power rule (which is closer to the linear rule) compared to the quadratic rule.
- **Effectiveness of the forces of leakage and increasing flow varies across graph families**: We observe that the effectiveness of leakage and increasing flow varies across graph types. For instance, across all non-linear rules considered, we observe that the percentage of instances with strictly smaller path leakage (path length resp.) is higher for $G(n,p)$ local and $G(n,p)$ local DAG graphs compared to $G(n,p)$ and $G(n,p)$ DAG graphs. We also observe that there are a few instances where these forces end up increasing the path leakage (path length resp.). For instance, with 1.1 power rule and for $G(n,p)$ local graph family, 1% of instances end up with increased leakage compared to the baseline. It is an interesting direction for future research to understand what graph properties affect the effectiveness of leakage and increasing flow with non-linear decision rules.

Decision rule	Graph family	% instances with unchanged path len.	% instances with smaller path len.	Avg. % change	Avg. % change opt.
1.1 power	$G(n,p)$ local	77	23	-4.6	-4.6
	$G(n,p)$ local DAG	22	78	-29	-29
	Grid	56	44	-8.3	-16.4
	$G(n,p)$	100	0	0	0
	$G(n,p)$ DAG	96	4	-1.3	-1.3
Quadratic	$G(n,p)$ local	83	17	-3.3	-4.4
	$G(n,p)$ local DAG	55	45	-13.8	-19.6
	Grid	85	12	-1.7	-15
	$G(n,p)$	100	0	0	0
	$G(n,p)$ DAG	99	1	-0.3	-2.4
Quadratic -with- offset	$G(n,p)$ local	72	28	-5.7	-6.5
	$G(n,p)$ local DAG	14	85	-38.8	-42
	Grid	27	71	-29.2	-39.6
	$G(n,p)$	100	0	0	0
	$G(n,p)$ DAG	92	8	-3.9	-5.4
Rank-edge	$G(n,p)$ local	99	1	-0.1	-0.1
	$G(n,p)$ local DAG	95	5	-0.9	-0.9
	Grid	83	17	-2.7	-2.7
	$G(n,p)$	100	0	0	0
	$G(n,p)$ DAG	100	0	0	0

Table 2: Effect of increasing flow with non-linear decision rules.

- **Non-linear decision rules can prefer short paths even in the absence of increasing flow**: We observe that non-linear decision rules considered end up finding relatively short paths even without increasing flow. Such a behaviour is consistent with past observations [3, 2] where various non-linear decision rules have been shown to find relatively short paths . For instance, with $G(n,p)$ graphs, we observe that all the non-linear rules considered end up finding the shortest path for all graph instances. However, increasing flow does help in nudging the dynamics towards shorter paths (when the dynamics does not already converge to the shortest path).
- **Faster convergence with non-linear decision rules compared to linear decision rules**: For the non-linear decision rules we considered, we observe that simulations take significantly fewer iterations to converge compared to linear decision rules. This is true for settings involving leakage as well as increasing flow. Moreover, we observe that convergence is faster with stronger non-linearity. For instance, we observe that convergence with quadratic rule is faster compared to 1.1 power rule. Recall that we also observed that the forces of leakage and increasing flow were more effective with weaker non-linearity. In this sense, the strength of non-linearity can be thought of as a useful knob to balance the trade-off between convergence time and effectiveness of forces of leakage and increasing flow.

C.2.3 Parameter setting and implementation details

In this section, we describe how we set various parameters for the non-linear decision rule simulations:

- **Graph parameter setting**: For the $G(n,p)$ and $G(n,p)$ DAG graphs, we set $n = 100$ and $p = 0.1$. For $G(n,p)$ local and $G(n,p)$ local DAG, we set $n = 100,\ p = 0.5$ and window size $k = 10$. For the grid graph, we consider a $10X10$ grid. Similar to the linear decision rule case, we plant a random shortest path to the graphs in increasing flow case so that the shortest path is unique.
- **Decay parameter and initial flow**: The decay parameter δ was set to 0.9. The initial forward flow

level at s and backward flow level at d was chosen uniformly at random from $(0.5, 1)$, and the initial flow at all other vertices was set to 0.

- **Leakage**: For the case when there is leakage at vertices, the leakage at each vertex is chosen uniformly at random from $(0, 0.1)$.
- **Increasing flow rate**: In the case of increasing flow, we increase the incoming forward and backward flow by a factor of 1.1 at each time step. Similar to the linear decision rule case, to avoid floating point overflow, we implement this by decreasing all other flow and pheromone levels by a factor of 1.1 except the incoming forward and backward flow. For any decision rule that only depends on the normalized pheromone levels , it is not difficult to see that only the relative values of flow and pheromone level matter, and our implementation gives rise to exactly the same dynamics (up to scaling) as when we increased the incoming forward and backward flow by a factor of 1.1. All the non-linear decision rules we consider here only depend on the normalized pheromone levels except the quadratic-with-offset rule. For the quadratic-with-offset rule, we additionally also scale down parameter c by 1.1 in each step, which makes the dynamics equivalent (up to scaling) to the case where incoming flow is multiplied by 1.1. When the value of pheromone level or flow at any edge becomes too small (smaller than the minimum allowed value for a 64 bit floating point number $\approx 10^{-323}$), we round it to zero.
- **Decision rule parameters**: The quadratic and 1.1 power rules do not have any parameters to be set. Quadratic-with-offset rule has a parameter c. Note that when c is large, the dynamics would not converge to a single path. We set c to be small enough such that the dynamics for most instances end up converging to a single path. Here, we consider the dynamics to have converged when 95% of the flow is going through a single path. For the leakage simulations, we set the value of $c = 0.25$ for all graph instances . For the increasing flow simulations, we set the value of $c = 1$ for the grid graph instances and $c = 0.5$ for all other graph instances. Rank-edge decision rule also has a parameter q. We set $q = 0.01$ for all our simulations.
- **Convergence criterion**: For all simulations (including linear rule simulations) except the quadratic-with-offset rule simulations, we consider the dynamics to have converged when 99% of the flow goes through a single path. With quadratic-with-offset rule, we keep this threshold slightly lower to 95%. If the threshold is too high for this rule, most instances would only converge to a path when c is fairly small. However, with small c, this rule would be similar to the quadratic rule. To differentiate the dynamics with this rule from the quadratic rule, we keep the threshold slightly lower to 95% which allows us to choose a slightly larger value of c.
- **Initial Pheromone levels**: We set initial pheromone level on all edges equal to one. Recall that with the linear rule, our simulations worked as expected even when the pheromone level at each edge is chosen uniformly at random from $(0, 1)$. However, for non-linear rules, we observed that for most instances, the simulations do not converge to a path when pheromone level is randomly initialized. In that sense, the linear rule seems more robust to pheromone initialization. Therefore, we run our simulations with non-linear rules with pheromone levels uniformly set to one so that the dynamics converges to a path.
- **Ignoring instances that do not converge to a path**: We discussed that we set the initial pheromone level uniformly to one so that the dynamics converges to a path. But even in this case, with non-linear decision rules, we observe that for a few instances the dynamics does not converge to a path. In our simulations, we ignored such instances, and only considered the instances where the dynamics converges to a path in both the baseline setting and the setting with leakage or increasing flow.

References

[1] J-L Deneubourg, Serge Aron, Simon Goss, and Jacques M Pasteels. "The self-organizing exploratory pattern of the argentine ant". In: *Journal of insect behavior* 3.2 (1990), pp. 159–168.

[2] Arjun Chandrasekhar, Deborah M Gordon, and Saket Navlakha. "A distributed algorithm to maintain and repair the trail networks of arboreal ants". In: *Scientific reports* 8.1 (2018), pp. 1–19.

[3] Simon Goss, Serge Aron, Jean-Louis Deneubourg, and Jacques Marie Pasteels. "Self-organized shortcuts in the Argentine ant". In: *Naturwissenschaften* 76.12 (1989), pp. 579–581.
